# Multiple UBXN family members inhibit retrovirus and lentivirus production and canonical NFκΒ signaling by stabilizing IκBα

**DOI:** 10.1371/journal.ppat.1006187

**Published:** 2017-02-02

**Authors:** Yani Hu, Kaitlin O’Boyle, Jim Auer, Sagar Raju, Fuping You, Penghua Wang, Erol Fikrig, Richard E. Sutton

**Affiliations:** Section of Infectious Diseases, Department of Internal Medicine, Yale University School of Medicine, New Haven, Connecticut, United States of America; Duke University Medical Center, UNITED STATES

## Abstract

UBXN proteins likely participate in the global regulation of protein turnover, and we have shown that UBXN1 interferes with RIG-I-like receptor (RLR) signaling by interacting with MAVS and impeding its downstream effector functions. Here we demonstrate that over-expression of multiple UBXN family members decreased lentivirus and retrovirus production by several orders-of-magnitude in single cycle assays, at the level of long terminal repeat-driven transcription, and three family members, UBXN1, N9, and N11 blocked the canonical NFκB pathway by binding to Cullin1 (Cul1), inhibiting IκBα degradation. Multiple regions of UBXN1, including its UBA domain, were critical for its activity. Elimination of UBXN1 resulted in early murine embryonic lethality. shRNA-mediated knockdown of UBXN1 enhanced human immunodeficiency virus type 1 (HIV) production up to 10-fold in single cycle assays. In primary human fibroblasts, knockdown of UBXN1 caused prolonged degradation of IκBα and enhanced NFκB signaling, which was also observed after CRISPR-mediated knockout of UBXN1 in mouse embryo fibroblasts. Knockout of UBXN1 significantly up- and down-regulated hundreds of genes, notably those of several cell adhesion and immune signaling pathways. Reduction in UBXN1 gene expression in Jurkat T cells latently infected with HIV resulted in enhanced HIV gene expression, consistent with the role of UBXN1 in modulating the NFκB pathway. Based upon co-immunoprecipitation studies with host factors known to bind Cul1, models are presented as to how UBXN1 could be inhibiting Cul1 activity. The ability of UBXN1 and other family members to negatively regulate the NFκB pathway may be important for dampening the host immune response in disease processes and also re-activating quiescent HIV from latent viral reservoirs in chronically infected individuals.

## Author summary

A family of human genes termed UBXN is thought to control many cellular processes, including protein destruction. While working with these proteins, we noticed several profoundly blocked the production of human immunodeficiency virus (HIV) and other, similar viruses. We delved into the how this occurs, and it appears that at least three of the proteins affect a central pathway in man’s immunologic response to viral and other pathogens, termed NFκB, by a mechanism not previously described. When UBXN1 protein levels were reduced, HIV synthesis was enhanced in certain cells. We also tested what happens when UBXN1 is removed or blocked in human and mouse cells, and saw consistent effects on NFκB. Removing UBXN1 from mouse cells changed the expression hundreds of genes (about 5% of all mouse genes), notably those involved in cell stickiness and also the immune response. Blocking UBXN1 in T cells harboring a silent HIV caused HIV to be resynthesized. We believe that the UBXN gene family members that negatively impact NFκB may be important for dampening immune responses and also re-animating silent HIV, important for curing or eliminating HIV in man.

## Introduction

The UBX family member proteins are thought to regulate diverse cellular processes, including protein stability and degradation. Members of the gene family include UBXN2a (also termed UBXD4), UBXN2b (p37), UBXN2c (UBXD10, p47, or UBX1), UBXN3a (FAF1 or UBXD12), UBXN3b (FAF2 or UBXD8), UBXN4 (UBXD2 or UBXDC1), UBXN6 (UBXD1 or UBXDC2), UBXN7 (UBXD7), UBXN8 (REP8 or UBXD6), UBXN9 (ASPCR1, RCC17, TUG, or UBXD9), UBXN10 (UBXD3), and UBXN11 (UBXD5, COA-1, or SOC). These proteins may be grouped into five evolutionary conserved families that are represented by UBXN1, UBXN2c, UBXN3a, UBXN6, and UBXN8. Perhaps the best studied family member is UBXN2c, known to play a crucial role in homotypic membrane fusion processes as an adaptor or co-factor for the AAA ATPase p97/valosin-containing protein (VCP) [1–6]. p97 is thought to control multiple aspects of cellular homeostasis, and recently dominant mutations in p97 that cause rare multisystem degenerative diseases with varied phenotypes have been linked to altered UBXN2c co-factor regulation [7]. The UBX domain of p47 interacts directly with p97/VCP [8], imitating ubiquitinated substrates of this chaperone [9].

Other UBXN family members have been implicated as co-factors that cooperate with p97 [10–14]. In *C*. *elegans*, UBXN1, UBXN2, and UBXN3 appear to redundantly control spermatogenesis via degradation of TRA-1A [12]. UBXN9, by modulating the activity of the p97-Ufd1-Npl4 complex, has been shown to be critical for the degradation of polyubiquitinated proteins via endoplasmic reticulum-associated protein misfolding pathway [15]. However, for most of the other UBXN family members the physiological or cellular function is poorly if at all understood. Of note, members of the UBXN1, UBXN2c, and UBXN3a families also possess an N-terminal UBA domain, thought to be involved in (but not restricted to) binding ubiquitin monomers and higher order forms [16].

Previously we had reported that UBXN1 negatively regulated RIG-I-like receptor (RLR) signaling by binding to MAVS and sterically blocking the interaction of MAVS with several downstream effectors [17]. The overall outcome of RLR pathway inhibition by UBXN1 was enhanced replication of several RNA viruses, including vesicular stomatitis, Sendai, West Nile, and Dengue. Subsequently, a separate group reported that UBXN1 inhibited TNFα-stimulated NF-κB signaling by cIAP recruitment, independent of VCP/p97 [18]. This action of UBXN1 blocked cIAP1 recruitment to TNFR1, inhibiting RIP1 polyubiquitination in response to TNFα.

NFκB signaling is central to the innate immune response in higher organisms, inducing expression of multiple genes via interferon signaling that inhibit pathogen replication, including human immunodeficiency virus type 1 (HIV) and several other viruses [19–22]. At the same time, HIV is dependent upon NFκB activation in order to promote viral RNA transcription—the viral long terminal repeat or LTR has two NFκB binding sites, and mutations or deletions in these binding sites modulate transcript initiation [23–25]. Stimulation of the canonical NFκB pathway requires phosphorylation and degradation of cytosolic IkBα, the latter by the 26S proteasome, and nuclear translocation of NFκB, comprised of a p50 and p65 heterodimer, to directly activate transcription of responsive genes [26–29]. Depending upon the model in vitro T cell system used, quiescent, genomically integrated HIV, which is transcriptionally silent and a barrier to viral eradication, can be stimulated out of latency by activation of the NFκB pathway [30–32]. Such transcriptional activation, coupled with other treatment modalities and immune recognition of HIV-infected cells, is a possible path towards HIV eradication and cure [33–38].

Over the years a number of viral and cellular negative regulators of NFκB signaling have been identified. One of the best known is A20, which functions as a ubiquitin editing gene, both adding and removing different polyubiquitin chains from NFκB signaling proteins [39–41]. Related to this mechanism of inhibition are the A20-Binding Inhibitors of NFκB (ABINs), which were originally identified as A20-binding proteins, and these are also thought to be involved in the negative feedback regulation of NFκB activation [42–43].

In the cytosol, NFκB heterodimer is held in an inactive state by IκBα, which after cell stimulation is phosphorylated by the activated IKK complex composed of NEMO and IKKα/β. Phosphorylated IκBα is recognized by the Cullin (Cul)1 E3 ubiquitin ligase scaffolding complex [44–46], which includes Skp1, Skp2 F-box (β–TrCP), and Rbx1, and IκBα undergoes polyubiquitination and subsequent 26S proteosomal degradation, allowing NFκB heterodimer nuclear translocation. Cul1 activity is itself regulated by the COP9 signalosome and neddylation [47–48]. Although it has been reported that rotavirus NSP1 induces proteosomal degradation of β–TrCP [49] and ORF2 of hepatitis E virus directly binds β–TrCP and stabilizes IκBα [50], we are unaware of any cellular factors known to obstruct or interfere with Cul1 function. Here we report that UBXN1 and other UBXN family members block the canonical NFκB signaling pathway and inhibit retroviral and lentiviral production via interaction with Cul1.

## Results

**Fig 1:**
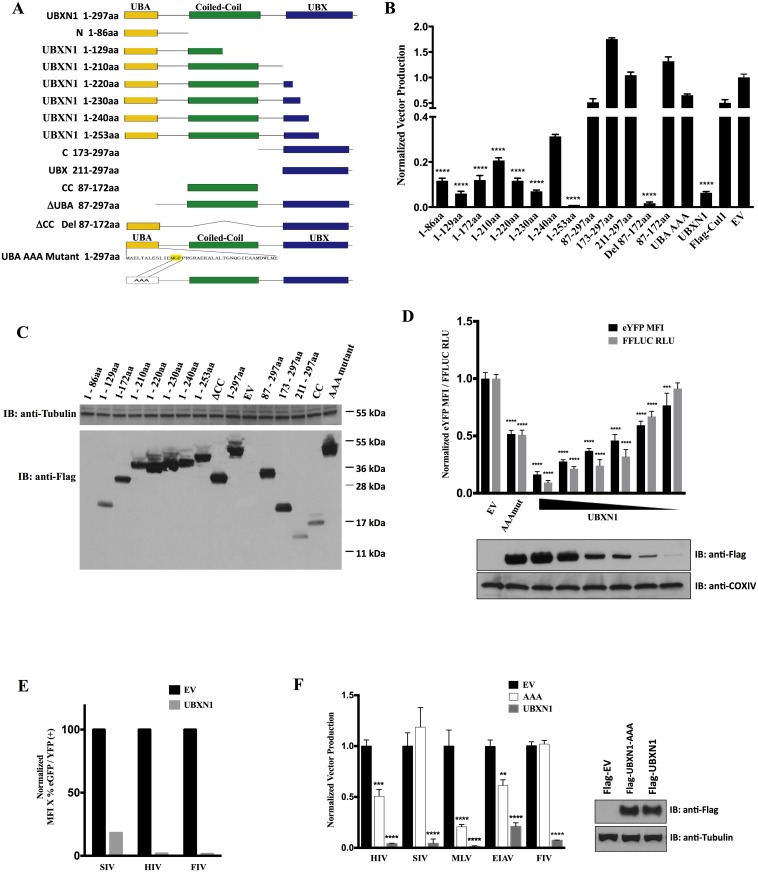
Overexpression of UBXN1 inhibits retrovirus production and NFκB pathway. **(A)** Schematic of UBXN1 deletion and mutant constructs: yellow is N terminal UBA domain thought to bind polyubiquitin, green is coil-colied domain thought to mediate self-association, blue is UBX domain thought to interact with other proteins; **(B)** Quantification of HIV-eYFP (VSV G) production, after 293T co-transfection of HIV vector components and UBXN1 expression plasmid shown at bottom, with titer on HOS cells normalized to empty plasmid (set at 1), as measured by FACS; **(C)** Immunoblot of the various FLAG-UBXN1 mutant proteins, as detected using anti-FLAG antibody; **(D)** Quantification by FACS of HIV LTR activity in 293T cells transfected with decreasing amounts of 1–297 UBXN1 along with HIV-eYFP, as measured by eYFP MFI, normalized to empty vector (EV) (black bars); also quantification by RLU of HIV LTR-FFLUC, also normalized to EV (grey bars); underneath is shown immunoblot of transfected FLAG-tagged UBXN1 wt and AAA mutant proteins; **(E)** Quantification of transfection efficiency in 293T producers of other lentiviral vectors, as measured by eGFP/eYFP MFI by FACS in the presence of either FLAG-UBXN1 (grey bars) or EV (black bars); **(F)** As in part **(B)**, quantification of production of indicated retroviral vectors, after co-transfection of FLAG-UBXN1 (grey bars), FLAG-AAA mutant (open bars), or EV (black bars) expression plasmids along with packaging and transfer vector components and VSV G into 293T cells, with resultant titer normalized to that of EV (set at 1.0 in each case). Shown to the right is a representative immunoblot of wt UBXN1 and AAA mutant. ****p < 0.0001, ***p < 0.0005, **p < 0.005, compared to EV, by two-way ANOVA.

To further investigate the biology of UBXN1, we constructed an HIV-based, third generation lentiviral vector encoding the full-length UBXN1 cDNA (297 aa) and attempted to produce VSV G-pseudotyped lentiviral vector particles in 293T cells by co-transfection of HIV packaging and VSV G expression plasmids, but resultant HIV vector titers were reduced by two orders-of-magnitude compared to control, empty vector. To verify this reduction in titer was not a result of an intrinsic defect to the vector perhaps due to inhibitory cis-acting sequences present within the UBXN1 open reading frame, we co-transfected UBXN1 expression plasmid along with an HIV transfer vector and VSV G envelope plasmid again into 293T cells and observed >100-fold reduction in resultant titer of the HIV vector on HOS targets, compared in parallel to the use of empty cDNA expression plasmid (Fig 1B). To exclude the possibility that the reduction in titer was a result of less functional VSV G being expressed in the producers, 293T cells transfected with VSV G were acid –shocked at pH 5.2 for two minutes and cell syncytia were enumerated an hour later, and there was no observable difference in the number of multinucleate 293T cells in the presence or absence of UBXN1.

To determine whether this reduction in titer was generalizable to other retroviral vectors, we tested other third generation, replication-defective retroviral vectors, including murine leukemia virus (MLV), feline immunodeficiency virus (FIV), simian immunodeficiency virus (SIV), and equine infectious anemia virus (EIAV), all in the presence or absence of co-transfected UBXN1 expression plasmid (Fig 1F). Each retroviral vector was produced in 293T cells using a three-component plasmid system comprised of VSV G, retroviral packaging vector driven by CMV immediate-early enhancer/promoter (IE), and retroviral transfer vector encoding either eGFP or eYFP. In each case, there was a significant decrease in retroviral vector titer in the presence of co-transfected UBXN1, as assessed by flow cytometry readout on target HOS cells 72 h post-transduction (Fig 1F).

To see whether other UBXN family members had a similar effect, we obtained or cloned ourselves CMV IE-driven, FLAG epitope tagged versions of p47, UBXD4, UBXN3a, UBXD8, UBXN4, UBXN6, UBXN7, UBXN8, UBXN9, UBXN10, and UBXN11 (for UBXN11 both rat and human cDNAs), and verified appropriate expression of each after transient transfection of 293T cells, although there was some variability in expression levels between each family member (S1a Fig). Each of these UBXN constructs was co-transfected with HIV or FIV transfer and packaging vector components as described above, along with VSV G, and reproducibly we observed significant, marked inhibition of lentiviral vector production when UBXN1, UBXD8, UBXN6, UBXN11 and UBXN9 were co-transfected and expressed (S1b and S1c Fig).

**Fig 2:**
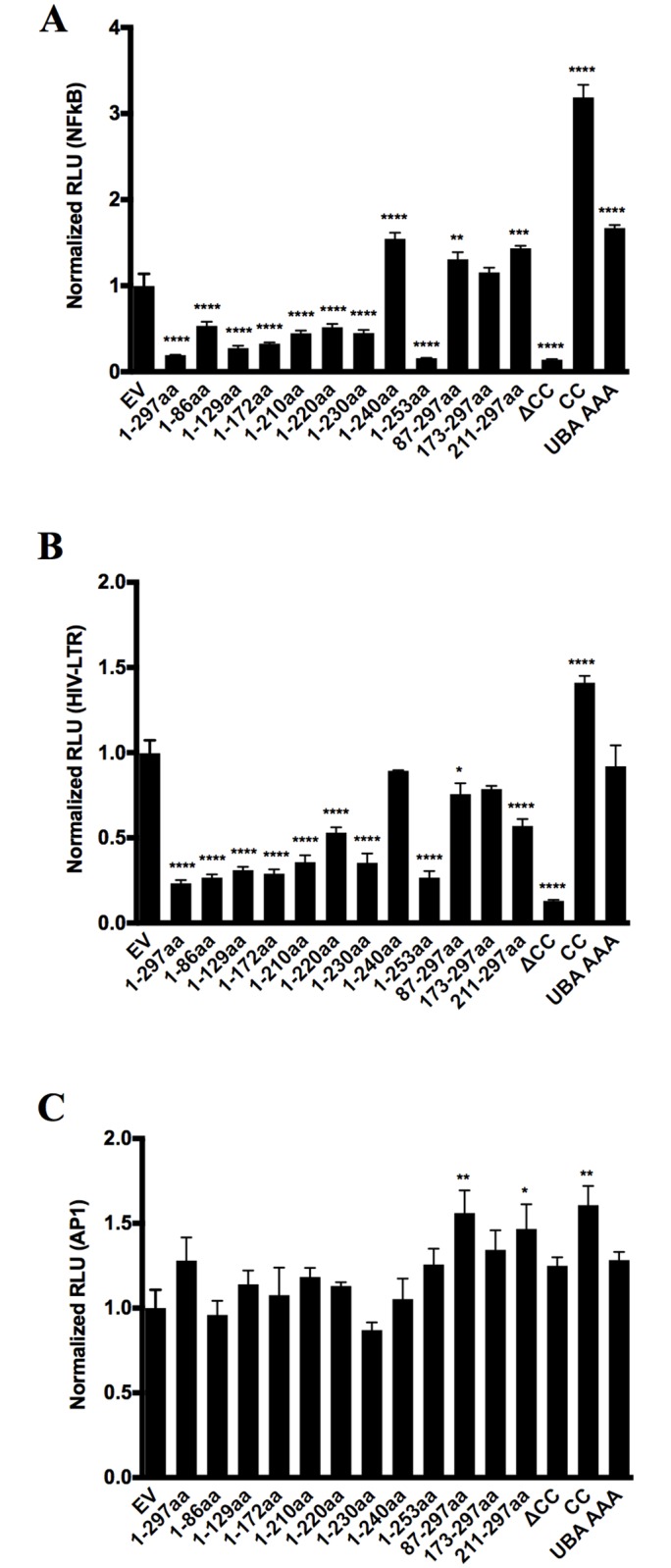
Characterization of UBXN1 functional domains by reporter assay. **(A)** Quantification of NFκB-FFLUC reporter in HEK293 cells 48 h after 96-well format transfection with indicated Myc-UBXN1 constructs (80 ng per well) or empty plasmid (Vector), in the presence 5 ng/mL TNFα for 4h; **(B)** Similar to **(A)**, using an HIV LTR-FFLUC reporter; **(C)** Similar to **(A)**, using an AP1-FFLUC reporter. All data represent mean ± SEM (n = 3). ****p < 0.0001, ***p < 0.0005, **p < 0.005, *p < 0.05, compared to vector alone, by two-way ANOVA.

In the HIV vector used in the above experiments, the autofluorescent reporter is driven off the viral long terminal repeat (LTR), and reporter gene expression was also significantly reduced in a dose-dependent manner by UBXN1 in 293T producer cells, which paralleled that of an HIV LTR-FFLUC construct (Fig 1D). This marked reduction in LTR activity was also observed for the SIV and FIV LTRs after plasmid transfection of 293T cells (Fig 1E). Because of this result, we tested whether UBXN1 and the four other family members directly affected transcriptional initiation of HIV and other retroviruses. After transient transfection into 293T cells of replication-defective HIV, SIV, and MLV vectors that all have LTRs driving eGFP, all five UBXN family members reduced LTR activity, with UBXD8 having the least amount of inhibitory activity (S1d Fig). Additionally, using firefly luciferase reporter transient transfection assays, full-length UBXN1 and other family members, including the four mentioned above, inhibited NFκB and HIV LTR activity but with rare exception had no or little effect on the AP-1 promoter (Fig 2A and 2B, S1e Fig).

**Fig 3:**
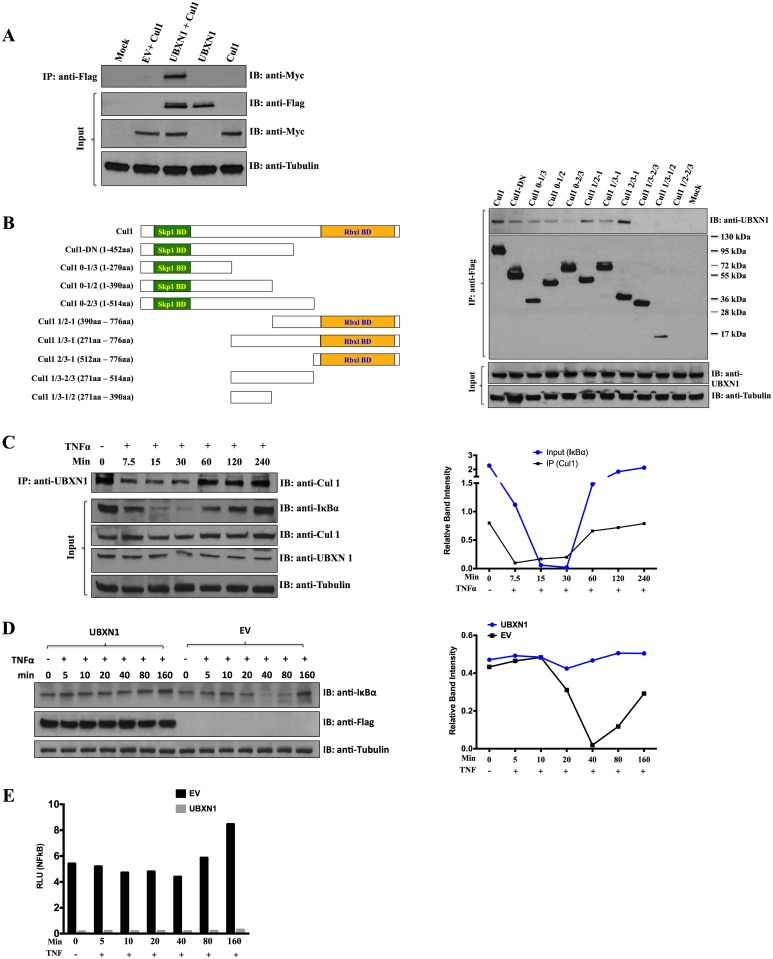
UBXN1 interacts with Cullin1 and prevents IκBα degradation. **(A)** FLAG-UBXN1 was co-transfected with Myc-Cul1, as indicated, and immunoprecipitated (IP) using anti-FLAG antibody, followed by immunoblotting (IB) using anti-FLAG or Myc antibody; β−tubulin served as loading control; **(B)** Left: schematic of Cul1 deletion constructs, with indicated regions that bind to Skp1 and Rbx1 (green and yellow, respectively, not precisely to scale); right: FLAG-Cul1 constructs were transfected into HEK293 cells, as indicated at top; endogenous UBXN1 was detected using anti-UBXN1 antibody and β−tubulin again served as loading control; **(C)** left: At top: Co-IP of endogenous UBXN1 and Cul1 from HFFs, after stimulating cells with 10 ng/mL TNFα; input of various proteins shown at bottom; right: relative band intensity of IκBα and Cul1, normalized against the density of β−tubulin (in the left immunoblot image); **(D)** left: Transfection of HEK293 cells with either empty vector or plasmid encoding FLAG-UBXN1, treated with 5 ng/mL TNFα for the indicated times, and cell lysates immunoblotted for all three indicated proteins, with β−tubulin serving as loading control; right: relative band intensity of IκBα normalized against the density of β−tubulin (in the left immunoblot image); **(E)** Similar to **(D)** except that NFκB-FFLUC reporter was co-transfected and cell lysates assayed for luciferase activity, normalized to co-transfected Renilla-LUC reporter, in the presence (grey bars) or absence (black bars) of UBXN1. Experiments of **(C)** and **(D)** were repeated at least twice, with similar results.

NFκB activity is controlled by nuclear translocation, which can only occur if IkBα is degraded via the 26S proteasome, mediated in part by the E3 ubiquitin ligase Cullin1 (Cul1) [51–52, 45]. Previously, UBXN1 had been shown to interact with several Cullins, including Cul1 (http://www.ebi.ac.uk/intact/pages/interactions/interactions.xhtml?query=Q04323*). When epitope-tagged versions of both UBXN1 and Cul1 were both over-expressed in 293 cells, they interacted with each other by co-immunoprecipitation (IP)-immunoblotting (Fig 3A). This co-IP-IB interaction was also observed between endogenous Cul1 and exogenous, epitope-tagged versions of UBXN1, UBXN9, and UBXN11 expressed in 293 cells (S2g Fig).

Cul1 is considered a scaffolding protein that binds to the adaptor protein Skp1 near its amino terminus and ring box protein Rbx1 near its carboxy terminus; the receptor protein Skp2_fbox_ binds to Skp1 and also to substrate protein. To determine which regions of Cul1 interacted with UBXN1, a series of Flag epitope-tagged derivatives of Cul1 were constructed and expression in 293 cells assessed by immunoblotting (Fig 3B, left and right). All Cul1 proteins expressed at detectable levels. By co-IP and immunoblotting in 293 cells, UBXN1 interacted with both the N and C-terminal thirds of Cul1, but not the middle third of the protein (3B, right). These results suggest that there are multiple points of contact between UBXN1 and Cul1.

We next examined the ability of the endogenous forms of both Cul1 and UBXN1 to interact with each other, before and after TNFα stimulation of primary, non-transformed cells. In the absence of TNFα, the two proteins co-IP in human foreskin fibroblasts (HFFs), but after TNFα stimulation there was visibly less interaction as seen by co-IP, which temporally coincided almost precisely with IkBα degradation (Fig 3C, quantified on the right). Once IkBα levels returned to baseline, so did the observed interaction between Cul1 and UBXN1. Total levels of Cul1 and UBXN1 were unaffected by TNFα treatment of HFF cells, nor were there any obvious changes in the mobility of either protein by SDS-PAGE (Fig 3C).

Degradation of IkBα is central to the activation of NFκB, which is mediated by Cul1 scaffolding complex of Skp1 adapter, Skp2_fbox_ receptor, and Rbx1, which binds to IkBα substrate and results in its ubiquitination and subsequent targeting to the 26S proteasome for degradation. Over-expression of UBXN1 completely blocked IkBα degradation (Fig 3D, quantified on the right), which mirrored the inhibition of NFκB activity (Fig 3E). This was also true for UBXN9 and UBXN11, but not UBXN6 (S2c and S2d Fig, quantified on the right). These results suggest that UBXN1, N9, and N11 but not UBXN6 block IkBα degradation, thus inhibiting NFκB activity.

In order to map the functional domains of UBXN1, previously we had made a series of epitope-tagged UBXN1 deletion constructs (Fig 1A) [17]. These constructs, which consisted of both N and C terminal truncations, and internal deletions of the UBA, UBX, and coiled-coiled domains to allow functional assessment of these three regions of UBXN1, were also tested in the above assays. Of note, reproducibly the shorter constructs were not well-expressed, either because they were unstable or due to poor transfer to the filter membrane (Fig 1C). The ability of UBXN1 to inhibit HIV-based vector production (Fig 1B), NFκB activity (Fig 2A), HIV LTR activity (Fig 2B), degradation of IκBα (S2a and S2b Fig), along with interaction with Cul1 (S2e and S2f Fig) mapped to its UBA domain (amino terminus), with a second important determinant between amino acids 240 and 253. One puzzling result is that the 1–240 aa construct was well-expressed and variably interacted with Cul1 but was less inhibitory in these assays compared to 1–230 aa and the 1–253 aa constructs, suggesting that it was not completely folded correctly or lacked necessary post-translational modifications. Neither the C terminus (amino acids 253–297) nor the coiled-coil domain (amino acids 87 to 172) of UBXN1, the latter of which mediates interaction between UBXN1 monomers [17], was necessary for these activities. A triple alanine (AAA) substitution mutation in the UBA domain thought to bind to ubiquitin multimers (amino acid positions 13–15) markedly reduced the inhibitory activity of UBXN1. This was true for HIV vector production (Fig 1B and S1b Fig), HIV LTR transcriptional activity (Figs 2B and 1D), production of other retroviral vectors with the possible exception of MLV (Fig 1F and S1b Fig), and NFkB transcriptional activity (Fig 2A). This AAA UBXN1 mutant is quite informative since it was very well expressed but clearly did not interact with Cul1 by co-IP (S2e and S2f Fig). This is consistent with the inhibitory activity of UBXN1 being mediated by interaction with Cul1.

In order to examine whether there is a genetic interaction between UBXN1 and Cul1, increasing amounts of UBXN1 and a dominant negative (DN), COOH-truncated form of Cul1 were co-transfected into 293T cells in the presence of an HIV LTR-FFLUC reporter. As expected, in the absence of the DN Cul1 increasing amounts of UBXN1 were inhibitory, in a dose-dependent fashion (S3 Fig). Additionally, increasing amounts of DN Cul1 in the absence of UBXN1 were also inhibitory, presumably because DN Cul1 interferes with the function of endogenous Cul1 in targeting IκBα for proteasomal degradation. Intermediate amounts of DN Cul1, however, were stimulatory in the presence of UBXN1, likely because the exogenous DN Cul1 interacts with UBXN1, blocking its inhibitory function against endogenous Cul1. Moreover, at the highest amounts of both UBXN1 and DN Cul1 there was no further inhibition of HIV LTR activity, suggesting that both are acting similarly, on the same pathway.

**Fig 4:**
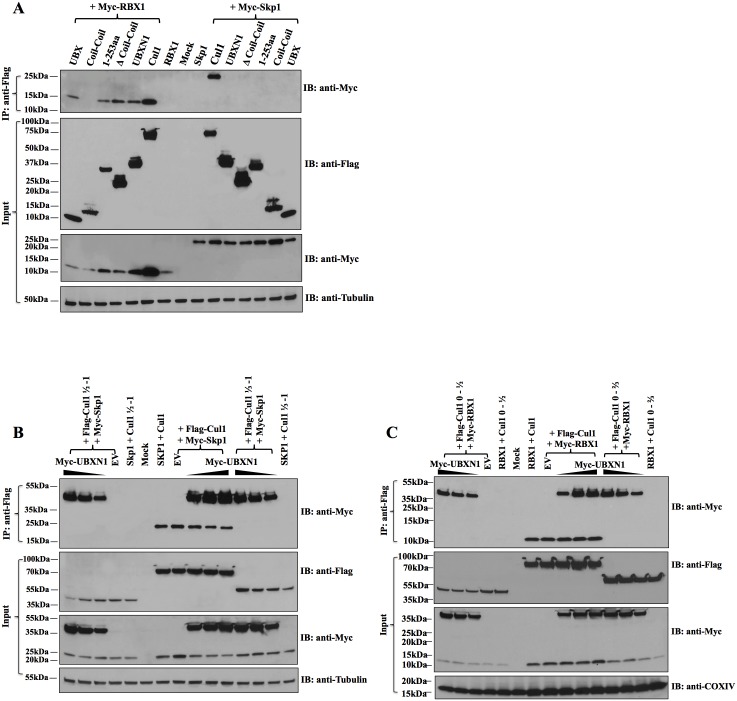
Co-immunoprecipitation of UBXN1, Rbx1, Skp1, and Cul1. **(A)** 293T cells were transiently transfected with the indicated FLAG-UBXN1 constructs or FLAG-Cul1 in the presence of either Myc-Rbx1 or Myc-Skp1 as indicated, with co-IP at top and input, including β-tubulin, at bottom; **(B)** Similar to **(A)**, except decreasing amounts of Myc-UBXN1 were transfected along with Myc-Skp1 and either empty vector (EV), ½-1 FLAG-Cul1, FLAG-Cul1, or 1/3-1 FLAG-Cul1, as indicated, with co-IP at top and input, including β-tubulin, at bottom; **(C)** Similar to **(B)**, except that Myc-Rbx1 was transfected along with EV, either 0-1/2 FLAG-Cul1, FLAG-Cul1, or 0-2/3 FLAG-Cul1, as indicated, with co-IP at top and input, including Cox IV, at bottom. Note that co-expression of Cul1 increased Rbx1 levels.

Both Skp1 and Rbx1 are known to bind to the N and C termini of Cul1, respectively, in order to target substrate proteins for proteosomal degradation. Because UBXN1 appeared to bind both the N and C termini of Cul1 as well, we investigated whether UBXN1 interacted with Skp1 or Rbx1 and whether UBXN1 interfered with either Skp1 or Rbx1 binding to Cul1. We constructed epitope-tagged versions of both Skp1 and Rbx1 cDNAs driven by the CMV promoter and co-transfected 293 cells with those along with UBXN1 and Cul1 expression plasmids. As anticipated, by co-immunoprecipitation Skp1 bound to full-length Cul1 (Fig 4A). Furthermore, Skp1 bound to Cul1 at the N-terminus, and increasing amounts of UBXN1 bound to Cul1 and yet did not displace Skp1 bound to Cul1 (Fig 4B). In addition, UBXN1 did not interact with Skp1.

As expected, Rbx1 bound Cul1 and also to various forms of UBXN1, but not the coiled-coil domain by itself (Fig 4A). Skp1 did not bind the latter half of Cul1 whereas UBXN1 did (Fig 4B), however the binding of Skp1 to full-length Cul1 was not displaced by increasing amounts of UBXN1 (Fig 4B). Also as expected Rbx1 bound to Cul1 at the C-terminus, and increasing amounts of UBXN1 did not displace Rbx1 bound to Cul1 (Fig 4C). UBXN1, however, did bind to Rbx1 by co-immunoprecipitation (Fig 4C).

We also examined whether there is a genetic interaction between Skp1 and UBXN1. Similar to the experiment with DN Cul1, increasing amounts of UBXN1 and full-length Myc-tagged Skp1 were co-transfected into 293T cells but in the presence of an HIV LTR-FFLUC reporter. As expected, in the absence of Myc-Skp1 increasing amounts of UBXN1 were inhibitory, in a dose-dependent fashion (S4 Fig). Increasing amounts of Myc-Skp1 were also inhibitory, likely because Myc-Skp1 is titrating away Skp2_fbox_ from the Cul1 scaffolding complex. In this case, however, at intermediate levels of Myc-Skp1 and increasing amounts of UBXN1 there was no stimulation in HIV LTR activity. This may be explained by the fact that both UBXN1 and Myc-Skp1 bind to Cul1 (Fig 4A) and at high amounts are inhibitory to Cul1 function. At the highest amounts of combined UBXN1 and Myc-Skp1 there was no further inhibition of HIV LTR activity, suggesting that both are indeed acting on the same pathway.

**Fig 5:**
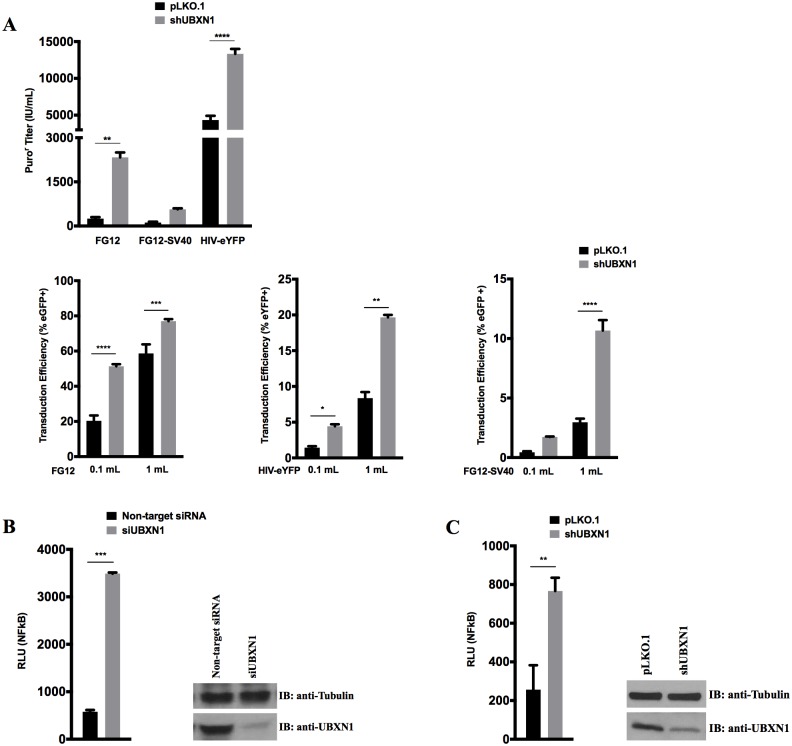
Knockdown UBXN1 results in enhanced retroviral vector production and NFκB signaling. **(A)** Quantification of HIV vector production after co-transfection of third-generation HIV-based vector encoding both anti-UBXN1 shRNA and puro^r^ gene (vs. control, empty vector), along with VSV G and either FG12, HIV-eYFP, or FG12-SV40 into 293T cells. FG12 is a third generation, self-inactivating HIV vector that encodes eGFP driven by the UbiC promote; FG12-SV40 is similar except it has an internal SV40 promoter driving eGFP. Top: puro^r^ titer on HOS targets; Bottom: percentage of eYFP/eGFP+ HOS targets as measured by flow cytometry for each of the three vectors, using two different amounts of indicated vector supernatant; **(B)** (left) Quantification of NFκB-FFLUC reporter in HEK293 cells after siRNA knockdown of UBXN1, compared to Trilencer-27 Universal scrambled negative control siRNA; (right) corresponding immunoblot of UBXN1 and β-tubulin in presence of either control or anti-UBXN1 siRNA. FFLUC values were normalized to those of co-transfected Renilla luciferase reporter; cells were stimulated for 4 h with 5 ng/mL of TNFα 48h post-transfection. (**C**) Similar to **(B)** except that either empty HIV vector pLK0.1 or vector encoding anti-UBXN1 shRNA was transfected into HEK293 cells, with corresponding immunoblot shown on right. Data represent mean ± SEM (n = 3). *p < 0.05, **p < 0.005, ***p < 0.0005, **** p<0.0001 by two-way ANOVA.

In order to examine the role of UBXN1 in a more physiological experimental setting, we first investigated the effect of knocking down UBXN1 using both shRNA and siRNA approaches in 293T cells. Validated shRNA against UBXN1 was ligated into an HIV-based vector, and when co-transfected into 293T cells with HIV packaging vector and VSV G expression plasmid the presence of the shRNA resulted in a 3 to 10-fold increase in viral titer of both the vector it was introduced into and also a separate HIV vector (encoding either eGFP or eYFP) used in parallel, compared to empty vector control (Fig 5A). This increase in titer was observed for all three of the separate HIV vectors that were co-transfected. The boost in titer of the anti-UBXN1 shRNA-encoding HIV vector is even more surprising considering that any shRNA-containing vector typically reduces titer by 10 to 30-fold, due to activation of RNA interference in the 293T cells [53]. siRNA and shRNA-encoded lentivirus vector-mediated knockdown of UBXN1 in 293 cells also resulted in significantly enhanced NFκB signaling (Fig 5B). The degree of knockdown using the siRNA against UBXN1 was ~80% whereas for the shRNA it was ~50% (Fig 5C).

We attempted to produce a murine conditional knockout animal, placing the LoxP-selection cassette in one of the small, 5' introns of murine *UBXN1*. Although we were able to obtain several heterozygous male and female mice, we were unable to obtain homozygous targeted animals, despite multiple matings and pup screenings. We also harvested fetuses from heterozygous matings, prepared mouse embryo fibroblasts (MEFs) at embryonic days 7, 11, 18, but only observed heterozygous (*UBXN1* +/-) or wild-type homozygous (*UBXN1* +/+) MEFs, as assessed by PCR using primers that spanned an inserted LoxP site (S2 Table). These results suggest that UBXN1 is essential for fetal development and eliminating the gene (or reducing its function) resulted in early embryonic lethality, to the point that homozygous null MEFs are not recoverable, even early on.

**Fig 6:**
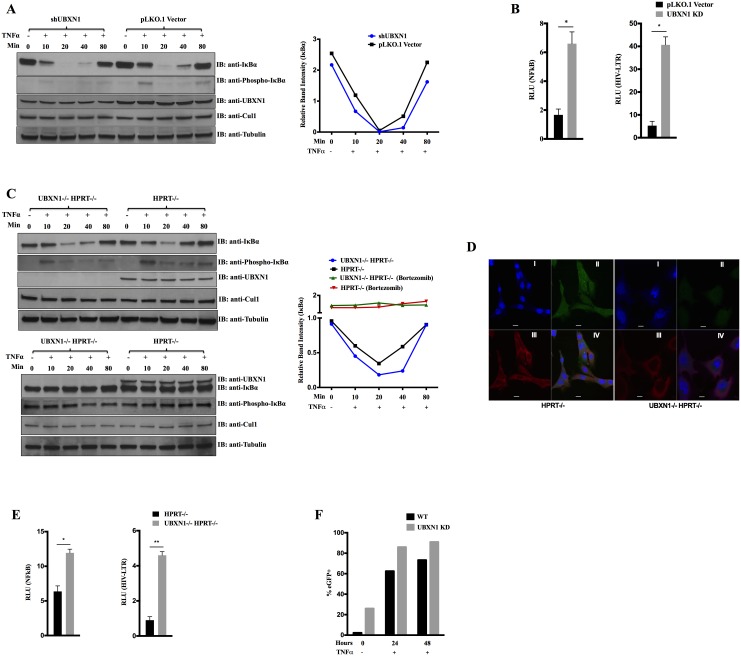
UBXN1 blocks NFκB signaling and inhibits HIV LTR activity. **(A)** Left: immunoblot of indicated proteins from HFFs stably transduced with either empty HIV-based vector or vector encoding anti-UBXN1 shRNA, after stimulation with 5 ng/mL TNFα for indicated times; right: relative band intensity of IκBα, normalized to β−tubulin (in the left immunoblot image); **(B)** Quantification of NFκB (left) and HIV LTR (right) FFLUC reporters in cell lines of **(A)**, normalized to co-transfected Renilla-LUC plasmid; for NFκB reporter cells were treated for 4 h with 5 ng/mL TNFα 48 h post-transfection; **(C)** Left: immunoblot of indicated proteins from UBXN1-/- HPRT-/- and control HPRT-/- MEFs, after stimulation with 10 ng/mL TNFα for indicated times (upper) or treated with 1 μM Bortezomib for 5 h and then stimulated with 10 ng/mL TNFα for the indicated times (lower); right: relative band intensity of IκBα, normalized to β−tubulin (in the left immunoblot images); **(D)** Confocal immunofluorescence microscopy of UBXN1 and Cul1 in UBXN1-/- HPRT-/- and control HPRT-/- MEFs. Nuclear DNA stained with TO-PRO-3 (I); UBXN1 (II) and Cul1 (III) were stained using secondary antibodies conjugated to Alexa Fluor 546 and 488, respectively; IV shows merge; Scale bar = 10 μm; **(E)** Quantification of NFκB and HIV LTR-FFLUC reporters in UBXN1-/- HPRT-/- and control HPRT-/- MEFs. Left: NFκB-FFLUC values 48 h post-transfection in the presence of 5 ng/mL TNFα for 4h, normalized to Renilla-LUC reporter; right: HIV LTR-FFLUC values 48 hrs post-transfection, similarly normalized; **(F)** JLAT10.6 T cells stably transduced with either HIV-based vector encoding anti-UBXN1 shRNA or control empty vector, after stimulating the cells with 5 ng/mL TNFα for the indicated times and measuring % eGFP+ by FACS. Data in **(B)** and **(E)** represent mean ± SEM (n = 3). **p < 0.005, *p < 0.05 by student’s t-test. Experiments of **(A)** and **(C)** were repeated several times, with similar results.

Instead, we decided to stably knockout or knockdown *UBXN1* in hTERT-immortalized MEFs and HFFs, respectively. To reduce UBXN1 expression in HFFs, we used the shRNA described above, encoded within the HIV-based vector, to produce stable HFF UBXN1 knockdown cells. After verifying ~50% knockdown (Fig 6A, quantified on right), HFFs were stimulated with TNFα. There was a subtle but quantifiable enhanced recovery of IκBα levels in control versus knockdown HFFs, which was evident at both early and late time points (Fig 6A). There were no differences in Cul1 levels between control and knockdown HFFs (Fig 6A). We also assessed NFκB signaling and HIV LTR activity in the UBXN1 KD HFFs, which were both increased, compared to HFFs transduced with empty vector control (Fig 6B). Of note, UBXN1 KD in HFFs or KO in MEFs did not modulate IkBα phosphorylation levels, most evident with the use of the proteosomal inhibitor Bortezomib, which suggests that UBXN1 does not inhibit or modulate IKK activity (Fig 6A and 6C).

To knockout *UBXN1* in the immortalized MEFs, we used Cas9/CRISPR and guide (g)RNAs targeting both a 5’ and 3’ exon of the gene simultaneously, along with a gRNA targeting a coding exon of murine *HPRT*. The latter allowed us to select knockout MEFs using 6-thioguanine (6TG) and enriched for *UBXN1* knockout clones several hundred fold (see Methods). Control MEFs had *HPRT* alone knocked out. Knockout of UBXN1 in MEFs was verified by immunoblotting (Fig 6C), PCR using flanking primers, and confocal immunofluorescence microscopy (Fig 6D). We were unable to obtain KO HFFs using a similar strategy that involved gRNA co-targeting of human UBXN1 along with *HPRT*. Although small (<100 cells) colonies resistant to 6TG arose in culture we were unable to propagate these HFFs; this may be related to altered cell cycle progression we observed in the KO MEFs (see below).

Knockout (both *UBXN1*-/- and *HPRT*-/-) MEFs appeared visibly larger and proliferated more slowly than *HPRT* -/- (control) MEFs (Fig 6D and S5a and S5b Fig). *UBXN1* KO MEFs were uninucleate but the nuclei were larger (Fig 6D). Cell cycle analysis of mid-logarithmic growth *UBXN1* KO MEFs indicated most were in G2/M, in sharp contrast to the *HPRT* KO MEFs, which had a normal cell cycle distribution (S5a and S5b Fig). Despite this, *UBXN1* KO cells were able to be passaged repeatedly, without evidence of senescence or progressive changes in morphology. After TNFα stimulation of *UBXN1* knockout MEFs, there was delayed recovery of IκBα protein levels, most obvious at the 10–40 min time points (Fig 6C, top left, quantified on right). In the presence of the proteasomal inhibitor Bortezomib after TNFα treatment there was no discernible degradation of IkBα, irrespective of the presence or absence of UBXN1 (Fig 6C, bottom left, quantified on right).

NFκB is well-known to induce IκBα in order to extinguish its own signaling, as an autoregulatory negative feedback mechanism. NFκB had significantly enhanced activity in the *UBXN1* KO MEFs, as did the HIV LTR reporter, compared to control cells (Fig 6E). Cul1 levels were unchanged in the *UBXN1* knockout MEFs, compared to control MEFs (Fig 6C). To test the reversibility of these effects, UBXN1 was added back to the *UBXN1* KO MEFs via lentiviral vector stable transduction. After doing so, there was an increase and stabilization of IκBα protein levels after stimulation of the cells with TNFα (S6a Fig, quantified on right). We also observed significant inhibition of both NFκB and HIV LTR activity after UBXN1 was added back to the *UBXN1* KO MEFs (S6b Fig).

In order to quantify the transcriptome after *UBXN1* KO, RNA-Seq was performed on both control and *UBXN1* KO MEFs. Nearly 800 genes were significantly up- and down-regulated in *UBXN1* KO compared to control MEFs (S3 Table). Using KEGG analysis, several gene pathways were significantly over-represented in terms of modulated genes, including focal adhesion, cytokine-cytokine receptor interaction, ECM-receptor interaction, chemokine signaling, and protein digestion and absorption (S4 Table and S7a and S7b Fig). Whether UBXN1 KO directly or indirectly modulated the levels of these genes in MEFs is difficult to ascertain at this time.

We next examined the effects of reducing UBXN1 expression on lentiviral and retroviral replication. Despite higher NFκB activity, compared to control MEFs *UBXN1* KO MEFs were significantly less susceptible in single round infectivity assays to VSV G-pseudotyped, replication-defective HIV, SIV, FIV, EIAV, and MLV VSV G-pseudotyped particles (S8a Fig). All of these vectors encoded an autofluorescent gene such that transduction was measured by flow cytometry. This was also true for replication-defective adenoviral vectors (S8b Fig), but the *UBXN1* KO MEFs were equally or more susceptible to infection by adeno-associated virus vector (S8c Fig), suggesting that the observed phenotype is not simply due to a general unhealthy or toxic cellular state. Similar results were observed with the KD MEFs (S8d Fig).

To examine the effects of reduced UBXN1 expression in a more physiological system related to HIV replication, we stably knocked down UBXN1 in C8166 T cells using the shRNA-encoding HIV vector described above, with ~50–80% KD confirmed by immunoblotting and RNA Seq, respectively (S9a Fig and S5 Table). The C8166 knockdown T cells had enhanced NFκB signaling and HIV LTR activity compared to control C8166 T cells (S9b Fig). Using replication-defective, single cycle HIV-based vectors pseudotyped with either VSV G or CXCR4-tropic HIV envelopes, we observed that KD C8166 T cells were equally susceptible to HIV infection, compared to control C8166 T cells (S9c Fig). This is likely because multiple transcription factors, not just NFκB, regulate transcription off the HIV LTR.

HIV latency in the memory T cell population is thought to be a major barrier to virus eradication in man, with multiple layers of regulatory control at the level of transcriptional initiation, including reduced activity of NFκB. In order to test the effects of UBXN1 on HIV latency, control or shRNA against UBXN1 was introduced by lentiviral vector transduction into JLAT 10.6 T cells, a Jurkat T cell-based line with an integrated but replication-defective HIV encoding eGFP that is a model of HIV latency. Degree of knockdown of UBXN1 was assessed to be ~80% by immunoblotting (S9e Fig). In control JLAT 10.6 T cells, percentage eGFP+ cells was ~3%, which increased to ~75% after 48 h of TNFα treatment, whereas shRNA UBXN1 JLAT 10.6 T cells at baseline were ~25% eGFP+, which increased to 90% after 48 h of TNFα stimulation (Fig 6F). shRNA UBXN1 JLAT 10.6 T cells did not appear to be more sensitive to TNFα treatment (S9f Fig), nor did they apoptose after TNFα stimulation. These results suggest that reduction in UBXN1 levels resulted in activation of NFκB, leading to enhanced HIV LTR-based transcription in this JLAT Jurkat cell line.

## Discussion

Although UBXN1 was previously thought to be involved in protein degradation and turnover [54–57], we have now shown that UBXN1 inhibits two key antiviral pathways, both RLR and NFκB signaling, by binding to cellular components and interfering with downstream effector functions. While other cellular, viral, and bacterial gene products are known to inhibit one or the other pathway, to our knowledge UBXN1 is unique in this regard and appears to tonically inhibit NFκB signaling by somehow interfering with Cul1 function in unstimulated cells.

Of interest, several other UBXN family members, notably N3b (D8), N6, N9, and N11, consistently and quite markedly inhibit the production of replication-defective HIV and other retroviruses from 293T cells. Although all four of those family members and UBXN1 have inhibitory activity directed against both NFκB and the HIV LTR, only UBXN1, UBXN9 and UBXN11 stabilized IkBα after TNFα stimulation. Those three family members also interacted with Cul1, at least by co-immunoprecipitation. That in *UBXN1* KO MEFs after TNFα stimulation there is enhanced degradation of IkBα but levels return to baseline suggests that there is some redundancy in the system. The fact that the four other UBXN family members were expressed in MEFs, with RPKM values ranging from ~2 to 30 (lowest for UBXN11), supports this contention. Of note, UBXN1 RPKM values in MEFs were much higher, at approximately 100. Precisely how UBXN family members N6 and N3b (D8) act to inhibit NFκB signaling and retrovirus production and whether different UBX gene family members can compensate for each other remains to be explored.

**Fig 7:**
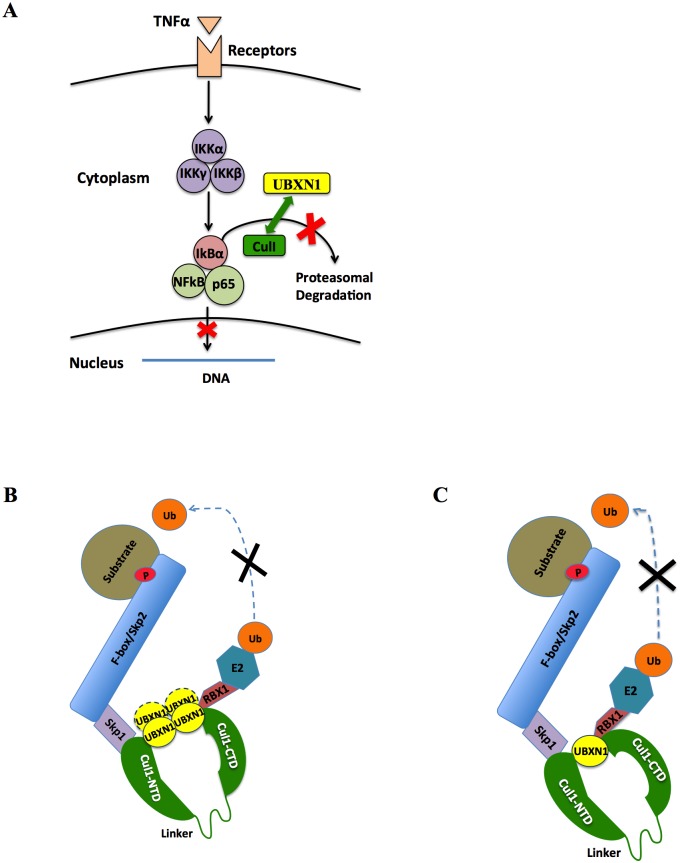
Schematic models of UBXN1 interaction with Cul1. **(A)** Cartoon rendering of how UBXN1 may be interfering with Cul1 in the canonical NFκB signaling pathway; **(B)** and **(C)** additional schematics of how UBXN1 may somehow be interfering with Cul1 activity either by steric hindrance **(B)** or allosteric effect **(C)**. UBXN1 is known to multimerize and interacts with both N and C termini of Cul1; stoichiometry between UBXN1 and Cul1 is not known (dashed ovals). In both models Skp1 and Rbx1 interact with the N and C termini of Cul1, respectively, and UBXN1 interacts with Rbx1 but not Skp1. These models do not exclude the possibility that another factor or protein (e.g., multimerized ubiquitin) mediates the interaction between UBXN1 and Cul1/Rbx1.

At this juncture we cannot exclude the possibility that UBXN1 has other, yet unknown inhibitory activities. For example, UBXN1 and the four other UBXN family members inhibited MLV LTR activity, and yet the MLV LTR does not have any NFκB sites. The mechanism of NFκB inhibition we describe here regarding IkBα stabilization is completely unrelated to and distinct from that recently reported by an independent group of investigators [18]. It is not entirely clear how UBXN1 becomes inactivated after TNFα cellular stimulation and then is no longer able to interact with Cul1 (or interacts only weakly with Cul1). Precisely how UBXN1 inhibits Cul1 function is not known. Based upon the fact that UBXN1 independently interacts with both the N and C-termini of Cul1, does not interact with Skp1 but does with Rbx1, and does not inhibit the binding of Skp1 or Rbx1 to Cul1, we present schematic models of how UBXN1 may be inhibiting the canonical NFκB pathway (Fig 7A). In one model, UBXN1 binds to Cul1 and multimerizes to somehow inhibit Cul1 function without much allosteric effect (Fig 7B); in the other, the presence of UBXN1 results in a conformational change in Cul1 between the N and C terminal domains, which are thought to be connected by a flexible linker region (Fig 7C). In both of these models Skp1 binds to Cul1 independently of UBXN1 whereas UBXN1 interacts with Rbx1 and Rbx1 also binds to Cul1. Whether UBXN1 interferes with the binding of other proteins or enzymes (i.e., Skp2 or E2 ubiquitin conjugating enzyme) to Skp1, Rbx1, or Cul1 remains to be established. Interaction between UBXN1 and Cul1 may be indirect, mediated by another protein(s) (e.g., ubiquitin multimers) or by a small molecule; experiments to examine direct binding of purified UBXN1 to Cul1 and Rbx1 are in progress. Whether UBXN1 also disrupts the function of other Cul family members is also under investigation. It is important to note that have we not yet observed any post-translational modifications of UBXN1 after cell stimulation that could potentially regulate its activity.

UBXN1 is not a viral restriction factor since it interacts with cellular proteins and not any viral gene products and in fact can enhance RNA virus replication [17]. Here it inhibits HIV and other lentivirus production in single cycle assays likely because virus production in 293T cells is very dependent upon NFκB binding to the viral LTR enhancer as part of the enhanceosome and stimulating viral mRNA biosynthesis. This underscores the delicate and intricate balance the NFkB pathway plays during HIV infection since it is absolutely required for high level virus production and yet the downstream effectors or interferon stimulated genes can be detrimental to virus replication. Thus, although much more HIV can be produced from activated CD4+ T cells, at later stages those same cells can block viral replication. With regards to quiescent memory T cells, thought to be the major reservoir of latent HIV and the chief barrier to cure [58–61], the data presented here knocking down UBXN1 gene expression in JLAT cells suggest that interfering with the function of UBXN1 and perhaps other family members may facilitate the reactivation of HIV gene expression by enhancing NFκB activity, as part of the ‘shock and kill’ strategy of eliminating latently HIV-infected T cells.

That UBXN1 blocks both the RLR and NFκB pathways suggests that it is involved in innate immunity. Thus far, examination of several thousand whole exome sequences from both the Yale and NHLBI collections have revealed no homozygous single nucleotide variants or indels within the UBXN1 coding sequence, consistent with its essential function in mouse embryogenesis. Perusal of the ExAC dbase demonstrates only a handful of homozygous missense mutations within the coding sequence of UBXN1 in man (http://exac.broadinstitute.org/gene/ENSG00000162191); several of these are conservative substitutions. It will be of interest to test the functionality of the other missense mutations and correlate with disease processes. It is possible that enhancement of UBXN1 function could ameliorate inflammatory disorders, certain forms of autoimmunity, and malignancy in which either the RLR or NFκB pathway has been activated [62]. Additionally, other UBXN family members could play heretofore unappreciated roles in innate and adaptive immunity.

## Materials and methods

### Plasmids

pNFκB-Luc, pRL-TK, and AP1-Luc reporters, and all UBXN1 expression plasmids were as previously described [17]. HIV LTR-Luc and Tat expression plasmids were kind gifts of Dr. Andrew Rice (Baylor College of Medicine). pcDNA3-MYC3-Cul1 and pCDN3-DN-hCul1-Flag were obtained from Addgene; to prepare a FLAG-tagged version the Cul1 insert was excised using flanking BamH1 sites, Klenowed, and inserted into FLAG-pcDNA3.1/Zeo (Invitrogen) at the EcoR5 site, and expression confirmed by transfection and immunoblotting using anti-FLAG antibody as described above. Cul1 truncation mutants were made by PCR amplification using various combinations of primer pairs (S1 Table) and PrimeStar HS DNA Polymerase (Takara), TOPO-cloned into pCR-Blunt II-TOPO vector (Invitrogen), and the insert sequenced by the dideoxy method. pSil-mHPRT-Tomato was a gift of Richard Flavell (Yale), and is based upon pSilencer-Tomato, with the gRNA sequence 5’-GATCCGAAAAAGTGTTTATTCCTCAGTTTAAGAGCTATGCTGGAAACAGCATAGCAAGTTTAAATAAGGCTAGTCCGTTATCAACTTGAAAAAGTGGCACCGAGTCGGTGCTTTTTTA-3’ inserted between the BamH1 and HindIII sites (underlined sequence targets second exon of murine *HPRT*). pSil-mUBXN1-Tomato-1 and pSil-mUBXN1-Tomato-2 were similarly constructed, with insert sequences being 5’-GATCCGCATCGAGGCTGCGATGGATGTTTAAGAGCTATGCTGGAAACAGCATAGCAAGTTTAAATAAGGCTAGTCCGTTATCAACTTGAAAAAGTGGCACCGAGTCGGTGCTTTTTTA-3’ and 5’-GATCCGCCTTCTGCTGTCCTCATTGGTTTAAGAGCTATGCTGGAAACAGCATAGCAAGTTTAAATAAGGCTAGTCCGTTATCAACTTGAAAAAGTGGCACCGAGTCGGTGCTTTTTTA-3’ (underlined sequences target third and last exon of murine *UBXN1*, respectively). HIV-CIY has been described [63]; NL4-3-Renilla is based upon HIV provirus pNL4-3, with a frameshift in Vpr at the unique EcoR1 site and the 1.0 kb coding sequence of Renilla luciferase inserted at the unique Xho1 site in the same 5’-3’ orientation as the other viral genes. FG12-SV40 is based upon the third generation HIV vector FG12 with the UbiC promoter replaced by a 0.35 kb SV40 origin-promoter. Both full-length Skp1 and Rbx1 were PCR-amplified from a HeLa cDNA library and cloned into three different expression plasmids such that the epitope tags (HA, FLAG, and Myc) were at the amino terminus; DNA sequence and protein expression of all six constructs were respectively confirmed by Sanger sequencing and immunoblotting, the latter after transient transfection of 293T cells.

### Cell lines and cell cycle analysis

Adherent or suspension cells were cultured in DMEM or RPMI (ThermoFisher Scientific), respectively, supplemented with 10–15% fetal calf serum (FCS, Life Technologies), ultraglutamine as needed, and antibiotics. Mouse embryo and human foreskin fibroblasts were immortalized using an HIV-based vector encoding hTERT, the catalytic subunit of human telomerase, or SV40 virus large T antigen, coupled by an internal ribosome entry site to *bsd*, and maintained in DMEM supplemented with 10 μg/ml blasticidin (Invivogen).

For cell cycle analysis, mid-logarithmic cells were lifted in PBS plus 2 mM EDTA, pelleted, resuspended in complete medium supplemented with 10 μg/mL Hoechst 33342, and incubated for 45 min at 37°C prior to data acquisition on an LSRII flow cytometer (B-D). Cell cycle analysis was performed using FlowJo software. Because MTT assay results were misleading due to the increased size of the *UBXN1* KO cells, for proliferation studies cells were plated at low density in replicates in 6-well format and enumerated manually every 48 h after trypsinization, using a hemocytometer and trypan blue exclusion dye staining, passaging as necessary.

### Reagents and antibodies

Rabbit anti-UBXN1 (HPA012669), mouse anti-tubulin (T5168), and mouse anti-flag (F1804) antibodies were from Sigma-Aldrich; mouse anti-Cullin1 (32–2400) was from Invitrogen; mouse anti-Cullin1 (sc-17775) was from Santa Cruz Biotechnology. Rabbit anti-Cul1 monoclonal antibody was from Novus Biologicals (NBP1-40523). Rabbit anti-Myc (71D10), mouse anti-IκBα (L35A5), mouse anti-NFκB p65 (L8F6), rabbit anti-COX IV (3E11), rabbit anti-β-tubulin (9F3), rabbit anti-phospho-IκBα (Ser32) were all from Cell Signaling Technology. Secondary antibodies used from Cell Signaling Technology were anti-mouse IgG-HRP (#7076) and anti-rabbit IgG-HRP (#7074). Rabbit anti-p65 RelA (10745-1-AP) were from Proteintech; Phosphatase Inhibitor Cocktail (5870) and human Tumor Necrosis Factor-α (8902) were from Cell Signaling Technology; Protein A/G Agarose (20423) was from Thermo Scientific, Protease Inhibitor Cocktail Tablets (Complete EDTA-free) were from Roche Life Sciences. Alexa Fluor 488 Goat anti-Rabbit IgG (H+L) Secondary Antibody (Cat #: A-11008), Alexa Fluor 546 Goat anti-Mouse IgG (H+L) Secondary Antibody (Cat #: A-11003), TO-PRO-3 Iodide (642/661) (Cat #: T3605), and ProLong Gold Antifade Mountant with DAPI (Cat #: P-36931) were from Life Technologies. For enhanced chemiluminescence (ECL) HyGLO Quick Spray Chemiluminescent HRP Antibody Detection Reagent (Cat #: E2400) and HyBlot CL Autoradiography Film (Cat #: E3012; both from Denville Scientific Inc) were used.

### Analysis of protein-protein interactions

Approximately 10^6^ HEK293 cells (from the American Type Culture Collection, Manassas, VA) were reverse transfected in 6 well plates with various expression plasmids using Lipofectamine 2000 (Life Technologies). Whole-cell extracts were prepared from transfected cells using lysis buffer (50mM Tris-HCl pH 7.4, 150mM NaCl, 2mM EDTA, 1% Triton X-100, and 0.1% SDS) and incubated overnight at 4°C with 1:100 dilution of mouse monoclonal anti-FLAG antibody, then bound to protein A/G PLUS-Agarose beads for 2 hr at 4°C. Beads were washed three times and proteins eluted by boiling for 10 min in SDS sample lysis buffer, electrophoresed on pre-made SDS-PAGE gradient gels, transferred to nitrocellulose membranes (Bio-Rad), and probed with anti-Cul1 rabbit monoclonal antibody (Novus Biologicals) as primary and anti-rabbit IgG-HRP as secondary to detect Cul1-UBXN1 interactions by ECL and autoradiography. For input protein expression, 10% of whole cell extracts were gel electrophoresed in parallel and probed with anti-FLAG, anti-Cul1, and anti-β-Tubulin antibodies.

For co-immunoprecipitation of endogenous proteins, ~2 × 10^6^ of immortalized human foreskin fibroblasts were stimulated with TNFα at a concentration of 10 ng/mL. Whole cell extracts (10 μg) were prepared at different time points using lysis buffer as described above, incubated overnight at 4°C with 1:100 dilution of rabbit anti-UBXN1 antibody (Sigma-Aldrich), bound to protein A/G Agarose beads, and further processed as described above.

For confocal immunofluorescence microscopy of UBXN1 and Cul1 in MEFs, cells were fixed in one of 8-chamber culture slides (Falcon, #354108) with 4% formaldehyde-PBS for 15 min at room temperature, rinsed in PBS, then immunostained according to Immunofluorescence General Protocol (http://www.cellsignal.com/contents/resources-protocols/immunofluorescence-general-protocol/if). Primary rabbit anti-UBXN1 (Sigma-Aldrich) was diluted by 1:400, mouse anti-Cul1 (Santa Cruz) was diluted by 1:100. UBXN1 and Cul1 were stained using 1:1000 diluted secondary antibodies conjugated to Alexa Fluor 546 and 488 (Life Technologies), respectively, and nuclear DNA stained using TO-PRO-3. Images were acquired using a Zeiss LSM 510 Meta (objective 633).

### RNA interference and gene knockout

Four pmol of siRNA directed against human UBXN1 (#SASI_Hs01_00134629, Sigma-Aldrich) or Trilencer-27 Universal scrambled negative control siRNA (SR30004, Origene) were reverse-transfected into HEK 293 cells using Lipofectamine 2000 in 12-well plates, together with luciferase reporter plasmids (80 ng NFkB-luc per well, for example, and 16 ng pRL-TK per well). Ninety-six h post transfection, cells were washed with PBS and lysed for Dual Glow Luciferase assay, following the manufacturer’s instructions (Promega). FFLUC RLU was normalized to that of Renilla.

In order to generate stable UBXN1 knockdown cell lines, UBXN1-specific shRNA duplex oligonucleotides were designed targeting the same sequence as the siRNA above (#SASI_Hs01_00134629, Sigma-Aldrich), and inserted into pLKO.1 cloning vector between Age1 and EcoR1 sites, and DNA sequence confirmed.

For shRNA vector production, 293T cells were transfected using the calcium phosphate method with 10 μg shRNA plasmid (or pLKO.1 empty cloning vector), 10 μg HIV-PV, and 10 μg VSV-G expression plasmid. Replication-defective particles were harvested 72 h later, filtered, and then used for transducing various cell lines. Stable knockdown cells were maintained in 10 μg/ml puromycin (Sigma-Aldrich), with other supplements as needed.

For knocking out UBXN1 in murine cells, 5 x 10^6^ immortalized MEFs were nucleofected using MEF reagent (Lonza) and A-024 Amaxa program with 5 μg of codon-optimized Cas9-eGFP (gift of Dr. Richard Flavell of Yale), 5 μg of pSil-mHPRT-Tomato, 5 μg of pSil- mUBXN1-Tomato-1 and 5 μg of pSil-gadRNA-mUBXN1-Tomato-2 or just 5 μg of pSil-mHPRT-Tomato. After 96 hr, cells were selected in 100 μM 6-thioguanine (Sigma-Aldrich). Surviving cells were diluted and seeded into 48-well plates to select for mouse knockout cell clones, expanded, and screened via immunoblot using anti-UBXN1 antibody. Genomic DNA was extracted from candidate knockout cell clones using DNeasy (Qiagen), and an ~4.0 kb region of murine UBXN1 PCR amplified using primers 5'-TGGAGAGCCTCATCGAGATGGGCTTT-3' and 5'-TGCCCTTCTCAGAAAGGCAG TTCTGG-3' with PrimeStar HS DNA Polymerase (Takara), TOPO-cloned into pCR-Blunt II-TOPO vector (Invitrogen), and both ends of the insert sequenced by the dideoxy method to confirm deletion or rearrangement of *UBXN1*.

### Replication-defective, pseudotyped virus production

VSV G-pseudotyped, replication-defective HIV particles were produced from 293T cells using calcium-phosphate transfection protocol as described [65], as were FIV-eGFP(VSV G) particles. In addition to VSV G expression plasmid, for production of replication-defective, single-cycle EIAV packaging and transfer vector plasmids were pCEV53B and SIN6.1CeGFPW, respectively (kind gifts of Dr. John Olsen, UNC-Chapel Hill), for MLV pHIT60 and pBabe-IRES-eYFP, for SIV pSIV-PV and pSIV-NIG (gifts of Dr. Hung Fan, UC Irvine).

### RNA-Seq, and pathway analysis

For deep sequencing of mRNA, total RNA from MEF UBXN1 knockout clones was extracted using RNeasy Mini Kit (Qiagen) according to the manufacture’s instructions. RNA-Seq was performed by Yale’s Stem Cell Center’s Genome core by preparing a cDNA library depleted of rRNA, using Illumina HiSeq2000 platform (50 nucleotide paired-end reads). Reads were mapped to the hg19 human reference genome using TopHat2 aligner [64], and results analyzed using CuffDiff pipeline to identify differentially expressed genes [65].

List of genes that were determined to be significantly differentially expressed by RNA Seq analysis between total RNA samples was uploaded to the WebGestalt web server {http://www.webgestalt.org} and analyzed for enriched KEGG pathways. Statistical significance was calculated by WebGestalt. Pathway graphics were generated using the KEGG website and performing pathway enrichment analysis.

## Supporting information

S1 Fig Characterization of UBXN family member constructs. **(a)** Immunoblot of FLAG-tagged UBXN family members after transient transfection of 293T cells, with β−tubulin serving as loading control; EV denotes empty vector; **(b)** Quantification of HIV-eYFP (VSV G) (closed bars) and FIV-eGFP (VSV G) (open bars) vector production, after 293T cell co-transfection of HIV/FIV vector components and UBXN1 or family member expression plasmid shown at bottom, with titer on HOS cells normalized to EV (set at 1, as measured by FACS 48 h post-transduction; **(c)** similar to **(b),** with quantification of HIV-CIY (VSV G) (left), EIAV-eGFP (VSV G) (right) vector production, using two different amounts of vector supernatant titered on HOS cells and normalized to empty plasmid (set at 1), as measured by FACS 48 h post-transduction; **(d)** quantification by FACS of LTR activity using eGFP/eYFP reporter for HIV, SIV, and MLV vectors after transfection of 293T cells, normalized to empty plasmid (set at 1), as assessed by FACS 48 h post-transfection; **(e)** left: quantification of NFκB-FFLUC (closed bars) and HIV LTR-FFLUC reporter (open bars) in HEK293 cells 48 h after 96-well format transfection with indicated Flag-UBXN family member plasmids or EV, after treatment with 5 ng/mL TNFα for 4h; right: similar but using an AP1-FFLUC reporter. All data represent mean ± SEM (n = 3). ****p < 0.0001, *p < 0.05 by two-way ANOVA.
(TIFF)

S2 Fig UBXN mutants and family members inhibit NFκB activity by stabilizing IκBα. **(a)** Left: immunoblot of cellular proteins after transfection of HEK293 cells with either empty plasmid, 1–253, or ΔCoiled-coil Myc-UBXN1 plasmids, treated with 5 ng/mL TNFα for the indicated times, with β−tubulin serving as loading control; right: relative band intensity of IκBα normalized to β−tubulin (in the left immunoblot image); **(b)** similar to **(a)** after transfection of HEK293 cells with either empty plasmid, Coiled-coil, or 211–297 Myc-UBXN1 plasmids; **(c)** similar to **(a)** after transfection of HEK293 cells with either empty, UBXN6, or UBXN9 FLAG-tagged plasmids, treated with 2 ng/mL TNFα for the indicated times, with β−tubulin again serving as loading control; **(d)** similar to **(a)** after transfection of HEK293 cells with either empty or UBXN11 FLAG-tagged plasmids; **(e, f)** top: co-immunoprecipitation of indicated FLAG-UBXN1 constructs with Myc-Cul1 after transfection of HEK293 cells; input of both tagged proteins is shown; **(g)** top: co-immunoprecipitation of endogenous Cul1 and FLAG-UBXN family members after transfection of HEK293 cells, using anti-FLAG antibody, followed by immunoblotting (IB) using anti-Cul1 or anti-FLAG antibody; input of tagged proteins shown; β−tubulin serves as loading control.
(TIFF)

S3 Fig Genetic interaction between Cul1 and UBXN1. 293T cells in 12-well format were transiently transfected with HIV LTR-FFLUC reporter and indicated amounts of DN Cul1 with either no UBXN1 (solid black), 0.125 μg UBXN1 (solid gray), 0.25 μg UBXN1 (dashed gray), or 0.5 μg UBXN1 (dashed black). Left: RLU readout 48 h post-transfection; right: immunoblots of tagged versions of UBXN1 and Cul1, with CoxIV serving as loading control.
(TIFF)

S4 Fig Genetic interaction between Skp1 and UBXN1. 293T cells in 12-well format were transiently transfected with HIV LTR-FFLUC reporter and indicated amounts of UBXN1 with either no Skp1 (solid black), 0.125 μg Skp1 (solid gray), 0.25 μg Skp1 (dashed black), or 0.5 μg Skp1 (dashed grey). Left: RLU readout 48 h post-transfection; right: immunoblots of tagged versions of UBXN1 and Skp1, with CoxIV serving as loading control.
(TIFF)

S5 Fig Knockout of UBXN1 Inhibits the Growth of MEFs. **(a)** Forward and side scatter profiles of mid-logarithmic UBXN1-/- HPRT-/- clones 3 and 15 and HPRT-/- MEFs (top) and Hoechst cell cycle analyses by FACS, with 2N, S, and 4N distributions (bottom); **(b)** Cell growth kinetics of indicated of UBXN1-/- HPRT-/- clones 3, 15, 23 and 39 and HPRT-/- MEFs.
(TIFF)

S6 Fig Add back UBXN1 to UBXN1 KO MEFs Inhibits NFκB and HIV-LTR activity. **(a)** Left: immunoblot of indicated proteins from UBXN1 knockout MEFs stably transduced with either empty or Flag-UBXN1 encoding HIV vector, after treatment with 5 ng/mL TNFα for the indicated times; right: relative band intensity of IκBα normalized to β−tubulin (in the left immunoblot image) **(b)** Quantification of normalized NFκB (left) and HIV LTR (right) FFLUC reporters in cell lines of **(a)**; for NFkB reporter, cells were treated for 4 h with 5 ng/mL TNFα 48 h post-transfection; data represent mean ± SEM (n = 3). **p < 0.005, *p < 0.05 by student’s t-test.
(TIFF)

S7 Fig Significantly Enriched KEGG Gene Pathways in UBXN1 Knockout MEFs. **(a)** Focal adhesion/ECM-receptor interaction pathway; **(b)** Chemokine signaling/cytokine-cytokine receptor interaction pathway. Genes significantly up- or down-regulated are highlighted in yellow, with log_2_ fold change indicated by the number of + or – symbols (i.e., ++++ indicates gene was up-regulated approximately 16-fold); in cases where more than one gene was significantly altered, red dashed line connects to the regulated genes.
(TIFF)

S8 Fig Susceptibility of UBXN1 KO MEFs to Various Viral Vectors. **(a)** Susceptibility of UBXN1 KO vs. control MEFs to VSV G-pseudotyped, replication-defective HIV, SIV, FIV, EIAV, and MLV. In all cases readout was by flow cytometry using transfer vector-encoded eGFP or eYFP reporter. All data represents mean ± SEM (n = 4); **(b)** Similar to **(a)**, using varying amounts of a first generation adenoviral vector encoding eGFP; **(c)** Similar to **(a)**, using varying amounts of a replication-defective AAV vector encoding eGFP; **(d)** Left: Susceptibility of UBXN1 stable KD vs. control MEFs to single cycle, VSV G-pseudotyped SIV, FIV, EIAV, and MLV, as in **(a)**; Right: Immunoblot of UBXN1 after knockdown by control or anti-UBXN1 shRNA HIV-based vector in MEF cells, with β-tubulin serving as loading control. All data represent mean ± SEM (n = 3). ****p < 0.0001, ***p < 0.0005, *p < 0.05 by two-way ANOVA.
(TIFF)

S9 Fig Characterization of UBXN1 Knockdown T Cell Lines. **(a)** Immunoblot of indicated proteins in C8166 T cells stably transduced with either HIV-based vector encoding shRNA against UBXN1 or empty vector; **(b)** Quantification of NFκB-FFLUC (left) and HIV LTR-FFLUC (right) reporters in UBXN1 KD vs. control C8166 T cells, data represent mean ± SEM (n = 3). **p < 0.005, *p < 0.05 by student’s t-test; **(c)** Susceptibility of UBXN1 KD (pools of KD C8166 T cells derived from three independent transductions as indicated vs. control vector-transduced C8166 T cells (closed bars) to replication-defective HIV-CIY pseudotyped with either VSV G, NL4-3, or HXB2 env, with readout of % eYFP+ by FACS 72 h post-transduction; titer normalized by to that of wild type C8166 cells (set at 100); **(d)** Immunoblot of indicated proteins in JLAT 10.6 T cells stably transduced with control vs. HIV vector encoding shRNA against UBXN1; **(e)** UBXN1 KD or control JLAT 10.6 T cells treated with increasing amounts of TNFα from 10 pg/mL to 1 ng/mL for 48 h, then analyzed by flow cytometry, with % eGFP+ as readout, indicative of latent HIV reactivation.
(TIFF)

S1 Table Primers for Amplifying Portions of Cul1. (PDF)

S2 Table Screening of UBXN1 Heterozygous Mating. (PDF)

S3 Table Genes Significantly Up- and Down-Regulated in UBXN1 Knockout MEFs. (PDF)

S4 Table KEGG Pathway Analysis of RNA-Seq UBXN1 KO vs. MEF control. (PDF)

## References

[1] Kano F., Kondo H., Yamamoto A., Tanaka A.R., Hosokawa N., Nagata K., and Murata M. The maintenance of the endoplasmic reticulum network is regulated by p47, a cofactor of p97, through phosphorylation by cdc2 kinase. Genes Cells 2005; 10, 333–344. 10.1111/j.1365-2443.2005.00837.x 15773896

[2] Kondo H., Rabouille C., Newman R., Levine T.P., Pappin D., Freemont P., and Warren G. p47 is a cofactor for p97-mediated membrane fusion. Nature 1997; 388, 75–78. 10.1038/40411 9214505

[3] Meyer H.H., Kondo H., and Warren G. The p47 co-factor regulates the ATPase activity of the membrane fusion protein, p97. FEBS Lett 1998; 437, 255–257. 9824302 10.1016/s0014-5793(98)01232-0

[4] Uchiyama K., Jokitalo E., Lindman M., Jackman M., Kano F., Murata M., Zhang X., and Kondo H. The localization and phosphorylation of p47 are important for Golgi disassembly-assembly during the cell cycle. J Cell Biol 2003; 161, 1067–1079. 10.1083/jcb.200303048 12810701 PMC2173005

[5] Uchiyama K., and Kondo H. p97/p47-Mediated biogenesis of Golgi and ER. J Biochem 2005; 137, 115–119. 10.1093/jb/mvi028 15749824

[6] Yuan X., Simpson P., McKeown C., Kondo H., Uchiyama K., Wallis R., Dreveny I., Keetch C., Zhang X., Robinson C., et al Structure, dynamics and interactions of p47, a major adaptor of the AAA ATPase, p97. Embo j 2004; 23, 1463–1473. 10.1038/sj.emboj.7600152 15029246 PMC391063

[7] Zhang X., Gui L., Zhang X., Bulfer S.L., Sanghez V., Wong D.E., Lee Y., Lehmann L., Lee J.S., Shih P.Y., et al Altered cofactor regulation with disease-associated p97/VCP mutations. Proc Natl Acad Sci U S A. 2015; 112, E1705–1714. 10.1073/pnas.1418820112 25775548 PMC4394316

[8] Yuan X., Shaw A., Zhang X., Kondo H., Lally J., Freemont P.S., and Matthews S. Solution structure and interaction surface of the C-terminal domain from p47: a major p97-cofactor involved in SNARE disassembly. J Mol Biol 2001; 311, 255–263. 10.1006/jmbi.2001.4864 11478859

[9] Rape M., Hoppe T., Gorr I., Kalocay M., Richly H., and Jentsch S. Mobilization of processed, membrane-tethered SPT23 transcription factor by CDC48 (UFD1/NPL4), a ubiquitin-selective chaperone. Cell 2001; 107, 667–677. 11733065 10.1016/s0092-8674(01)00595-5

[10] Nagahama M., Ohnishi M., Kawate Y., Matsui T., Miyake H., Yuasa K., Tani K., Tagaya M., and Tsuji A. UBXD1 is a VCP-interacting protein that is involved in ER-associated degradation. Biochem Biophys Res Commun 2009; 382, 303–308. 10.1016/j.bbrc.2009.03.012 19275885

[11] Orme C.M., and Bogan J.S. The ubiquitin regulatory X (UBX) domain-containing protein TUG regulates the p97 ATPase and resides at the endoplasmic reticulum-golgi intermediate compartment. J Biol Chem 2012; 287, 6679–6692. 10.1074/jbc.M111.284232 22207755 PMC3307297

[12] Sasagawa Y., Yamanaka K., Saito-Sasagawa Y., and Ogura T. Caenorhabditis elegans UBX cofactors for CDC-48/p97 control spermatogenesis. Genes Cells 2010; 15, 1201–1215. 10.1111/j.1365-2443.2010.01454.x 20977550

[13] Schuberth C., and Buchberger A. UBX domain proteins: major regulators of the AAA ATPase Cdc48/p97. Cell Mol Life Sci 2008; 65, 2360–2371. 10.1007/s00018-008-8072-8 18438607 PMC11131665

[14] Trusch F., Matena A., Vuk M., Koerver L., Knaevelsrud H., Freemont P.S., Meyer H., and Bayer P. The N-terminal Region of the Ubiquitin Regulatory X (UBX) Domain-containing Protein 1 (UBXD1) Modulates Interdomain Communication within the Valosin-containing Protein p97. J Biol Chem 2015; 290, 29414–29427. 10.1074/jbc.M115.680686 26475856 PMC4705944

[15] Alberts S.M., Sonntag C., Schafer A., and Wolf D.H. Ubx4 modulates cdc48 activity and influences degradation of misfolded proteins of the endoplasmic reticulum. J Biol Chem 2009; 284, 16082–16089. 10.1074/jbc.M809282200 19359248 PMC2713509

[16] Buchberger A. From UBA to UBX: new words in the ubiquitin vocabulary. Trends Cell Biol 2002; 12, 216–221. 12062168 10.1016/s0962-8924(02)02269-9

[17] Wang P., Yang L., Cheng G., Yang G., Xu Z., You F., Sun Q., Lin R., Fikrig E., and Sutton R.E. UBXN1 interferes with Rig-I-like receptor-mediated antiviral immune response by targeting MAVS. Cell reports 2013; 3, 1057–1070. 10.1016/j.celrep.2013.02.027 23545497 PMC3707122

[18] Wang Y.B., Tan B., Mu R., Chang Y., Wu M., Tu H.Q., Zhang Y.C., Guo S.S., Qin X.H., Li T., et al Ubiquitin-associated Domain-containing Ubiquitin Regulatory X (UBX) Protein UBXN1 Is a Negative Regulator of Nuclear Factor kappaB (NF-kappaB) Signaling. J Biol Chem 2015; 290, 10395–10405. 10.1074/jbc.M114.631689 25681446 PMC4400349

[19] Hughes R., Towers G., and Noursadeghi M. Innate immune interferon responses to human immunodeficiency virus-1 infection. Rev Med Virol 2012; 22, 257–266. 10.1002/rmv.1708 22359246

[20] Marsili G., Remoli A.L., Sgarbanti M., Perrotti E., Fragale A., and Battistini A. HIV-1, interferon and the interferon regulatory factor system: an interplay between induction, antiviral responses and viral evasion. Cytokine & growth factor reviews 2012; 23, 255–270. 22748237 10.1016/j.cytogfr.2012.06.001

[21] Pitha P.M. Innate antiviral response: role in HIV-1 infection. Viruses 2011; 3, 1179–1203. 10.3390/v3071179 21994776 PMC3185785

[22] Rustagi A., and Gale M. Jr. Innate antiviral immune signaling, viral evasion and modulation by HIV-1. J Mol Biol 2014; 426, 1161–1177. 10.1016/j.jmb.2013.12.003 24326250 PMC7425209

[23] Griffin G.E., Leung K., Folks T.M., Kunkel S., and Nabel G.J. Activation of HIV gene expression during monocyte differentiation by induction of NF-kappa B. Nature 1989; 339, 70–73. 10.1038/339070a0 2654643

[24] Osborn L., Kunkel S., and Nabel G.J. Tumor necrosis factor alpha and interleukin 1 stimulate the human immunodeficiency virus enhancer by activation of the nuclear factor kappa B. Proc Natl Acad Sci U S A 1989; 86, 2336–2340. 2494664 10.1073/pnas.86.7.2336 PMC286907

[25] Roulston A., Lin R., Beauparlant P., Wainberg M.A., and Hiscott J. Regulation of human immunodeficiency virus type 1 and cytokine gene expression in myeloid cells by NF-kappa B/Rel transcription factors. Microbiol Rev 1995; 59, 481–505. 7565415 10.1128/mr.59.3.481-505.1995 PMC239370

[26] Campbell, K.J., and Perkins, N.D. Regulation of NF-kappaB function. Biochemical Society symposium, 2006; 165–180. 10.1042/bss0730165 16626297

[27] Hacker H., and Karin M. Regulation and function of IKK and IKK-related kinases. Science's STKE: signal transduction knowledge environment 2006; re13 10.1126/stke.3572006re13 17047224

[28] Skaug B., Jiang X., and Chen Z.J. The role of ubiquitin in NF-kappaB regulatory pathways. Annual review of biochemistry 2009; 78, 769–796. 10.1146/annurev.biochem.78.070907.102750 19489733

[29] Wegener E., and Krappmann D. Dynamic protein complexes regulate NF-kappaB signaling. Handb Exp Pharmacol 2008; 237–259. 10.1007/978-3-540-72843-6_10 18491055

[30] Hakre S., Chavez L., Shirakawa K., and Verdin E. HIV latency: experimental systems and molecular models. FEMS microbiology reviews 2012; 36, 706–716. 10.1111/j.1574-6976.2012.00335.x 22372374 PMC3563430

[31] Jiang G., and Dandekar S. Targeting NF-kappaB signaling with protein kinase C agonists as an emerging strategy for combating HIV latency. AIDS Res Hum Retroviruses 2015; 31, 4–12. 10.1089/AID.2014.0199 25287643 PMC4287114

[32] Jordan A., Bisgrove D., and Verdin E. HIV reproducibly establishes a latent infection after acute infection of T cells in vitro. Embo J 2003; 22, 1868–1877. 10.1093/emboj/cdg188 12682019 PMC154479

[33] Dahabieh M.S., Battivelli E., and Verdin E. Understanding HIV latency: the road to an HIV cure. Annu Rev Med 2015; 66, 407–421. 10.1146/annurev-med-092112-152941 25587657 PMC4381961

[34] Hezareh M.. Prostratin as a new therapeutic agent targeting HIV viral reservoirs. Drug news & perspectives 2005; 18, 496–500. 16391719 10.1358/dnp.2005.18.8.944543

[35] Remoli A.L., Marsili G., Battistini A., and Sgarbanti M. The development of immune-modulating compounds to disrupt HIV latency. Cytokine & growth factor reviews 2012; 23, 159–172. 22766356 10.1016/j.cytogfr.2012.05.003

[36] Sanchez-Duffhues G., Vo M.Q., Perez M., Calzado M.A., Moreno S., Appendino G., and Munoz E. Activation of latent HIV-1 expression by protein kinase C agonists. A novel therapeutic approach to eradicate HIV-1 reservoirs. Current drug targets 2011; 12, 348–356. 20955147 10.2174/138945011794815266

[37] Shan L., and Siliciano R.F. From reactivation of latent HIV-1 to elimination of the latent reservoir: the presence of multiple barriers to viral eradication. BioEssays: news and reviews in molecular, cellular and developmental biology 2013; 35, 544–552. 10.1002/bies.201200170 PMC4386637 23613347

[38] Siliciano R.F., and Greene W.C. HIV latency. Cold Spring Harbor perspectives in medicine 2011; 1, a007096 10.1101/cshperspect.a007096 22229121 PMC3234450

[39] Heyninck K., and Beyaert R. A20 inhibits NF-kappaB activation by dual ubiquitin-editing functions. Trends Biochem Sci 2005; 30, 1–4. 10.1016/j.tibs.2004.11.001 15653317

[40] Vereecke L., Beyaert R., and van Loo G. The ubiquitin-editing enzyme A20 (TNFAIP3) is a central regulator of immunopathology. Trends Immunol 2009; 30, 383–391. 10.1016/j.it.2009.05.007 19643665

[41] Wertz I., and Dixit V. A20--a bipartite ubiquitin editing enzyme with immunoregulatory potential. Adv Exp Med Biol 2014; 809, 1–12. 25302362 10.1007/978-1-4939-0398-6_1

[42] Verstrepen L., Carpentier I., and Beyaert R. The biology of A20-binding inhibitors of NF-kappaB activation (ABINs). Adv Exp Med Biol 2014; 809, 13–31. 25302363 10.1007/978-1-4939-0398-6_2

[43] Verstrepen L., Carpentier I., Verhelst K., and Beyaert R. ABINs: A20 binding inhibitors of NF-kappa B and apoptosis signaling. Biochemical pharmacology 2009; 78, 105–114. 10.1016/j.bcp.2009.02.009 19464428

[44] Deshaies R.J. SCF and Cullin/Ring H2-based ubiquitin ligases. Annual review of cell and developmental biology 1999; 15, 435–467. 10.1146/annurev.cellbio.15.1.435 10611969

[45] Jackson P.K., and Eldridge A.G. The SCF ubiquitin ligase: an extended look. Mol Cell 2002; 9, 923–925. 12049727 10.1016/s1097-2765(02)00538-5

[46] Tanaka K., Kawakami T., Tateishi K., Yashiroda H., and Chiba T. Control of IkappaBalpha proteolysis by the ubiquitin-proteasome pathway. Biochimie 2001; 83, 351–356. 11295496 10.1016/s0300-9084(01)01237-8

[47] Cope G.A., and Deshaies R.J. COP9 signalosome: a multifunctional regulator of SCF and other cullin-based ubiquitin ligases. Cell 2003; 114, 663–671. 14505567 10.1016/s0092-8674(03)00722-0

[48] Ohh M., Kim W.Y., Moslehi J.J., Chen Y., Chau V., Read M.A., and Kaelin W.G. Jr. An intact NEDD8 pathway is required for Cullin-dependent ubiquitylation in mammalian cells. EMBO Rep 2002; 3, 177–182. 10.1093/embo-reports/kvf028 11818338 PMC1083969

[49] Graff J.W., Ettayebi K., and Hardy M.E. Rotavirus NSP1 inhibits NFkappaB activation by inducing proteasome-dependent degradation of beta-TrCP: a novel mechanism of IFN antagonism. PLoS Pathog 2009; 5, e1000280 10.1371/journal.ppat.1000280 19180189 PMC2627925

[50] Surjit M., Varshney B., and Lal S.K. The ORF2 glycoprotein of hepatitis E virus inhibits cellular NF-kappaB activity by blocking ubiquitination mediated proteasomal degradation of IkappaBalpha in human hepatoma cells. BMC biochemistry 2012; 13, 7 10.1186/1471-2091-13-7 22590978 PMC3506457

[51] Cardozo T., and Pagano M. The SCF ubiquitin ligase: insights into a molecular machine. Nat Rev Mol Cell Biol 2004; 5, 739–751. 10.1038/nrm1471 15340381

[52] Ho M.S., Tsai P.I., and Chien C.T. F-box proteins: the key to protein degradation. Journal of biomedical science 2006; 13, 181–191. 10.1007/s11373-005-9058-2 16463014

[53] Poluri A., and Sutton R.E. Titers of HIV-based vectors encoding shRNAs are reduced by a dicer-dependent mechanism. Mol Ther 2008; 16, 378–386. 10.1038/sj.mt.6300370 18071333

[54] Besche H.C., Haas W., Gygi S.P., and Goldberg A.L. Isolation of mammalian 26S proteasomes and p97/VCP complexes using the ubiquitin-like domain from HHR23B reveals novel proteasome-associated proteins. Biochemistry 2009; 48, 2538–2549. 10.1021/bi802198q 19182904 PMC3811022

[55] Ishibashi T., Ogawa S., Hashiguchi Y., Inoue Y., Udo H., Ohzono H., Kato A., Minakami R., and Sugiyama H. A novel protein specifically interacting with Homer2 regulates ubiquitin-proteasome systems. J Biochem 2005; 137, 617–623. 10.1093/jb/mvi074 15944415

[56] LaLonde D.P., and Bretscher A. The UBX protein SAKS1 negatively regulates endoplasmic reticulum-associated degradation and p97-dependent degradation. J Biol Chem 2011; 286, 4892–4901. 10.1074/jbc.M110.158030 21135095 PMC3039385

[57] Wu-Baer F., Ludwig T., and Baer R. The UBXN1 protein associates with autoubiquitinated forms of the BRCA1 tumor suppressor and inhibits its enzymatic function. Mol Cell Biol 2010; 30, 2787–2798. 10.1128/MCB.01056-09 20351172 PMC2876507

[58] Coiras M., Lopez-Huertas M.R., Perez-Olmeda M., and Alcami J. Understanding HIV-1 latency provides clues for the eradication of long-term reservoirs. Nat Rev Microbiol 2009; 7, 798–812. 10.1038/nrmicro2223 19834480

[59] Finzi D., Hermankova M., Pierson T., Carruth L.M., Buck C., Chaisson R.E., Quinn T.C., Chadwick K., Margolick J., Brookmeyer R., et al Identification of a reservoir for HIV-1 in patients on highly active antiretroviral therapy [see comments]. Science 1997; 278, 1295–1300. 9360927 10.1126/science.278.5341.1295

[60] Margolis D.M. How Might We Cure HIV? Current infectious disease reports 2014; 16, 392 10.1007/s11908-014-0392-2 24562540

[61] Siliciano R.F. What do we need to do to cure HIV infection. Top HIV Med 2010; 18, 104–108. 20921575

[62] Yamamoto Y., and Gaynor R.B. Role of the NF-kappaB pathway in the pathogenesis of human disease states. Current molecular medicine 2001; 1, 287–296. 11899077 10.2174/1566524013363816

[63] Elinav H., Wu Y., Coskun A., Hryckiewicz K., Kemler I., Hu Y., Rogers H., Hao B., Ben Mamoun C., Poeschla E., Sutton R. Human CRM1 Augments Production of Infectious Human and Feline Immunodeficiency Viruses from Murine Cells. J Virol 2012; 86, 12053–12068. 10.1128/JVI.01970-12 22933280 PMC3486471

[64] Kim D., Pertea G., Trapnell C., Pimentel H., Kelley R., Salzberg S. L. TopHat2: accurate alignment of transcriptomes in the presence of insertions, deletions and gene fusions. Genome Biol 2013; 14, R36 10.1186/gb-2013-14-4-r36 23618408 PMC4053844

[65] Trapnell C., Hendrickson D. G., Sauvageau M., Goff L., Rinn J. L., Pachter L. Differential analysis of gene regulation at transcript resolution with RNA-seq. Nat Biotechnol 2013; 31, 46–53. 10.1038/nbt.2450 23222703 PMC3869392

