# Supplementary figures and images for: Multiple UBXN family members inhibit retrovirus and lentivirus production and canonical NFκΒ signaling by stabilizing IκBα
Source: PLoS Pathog. 2017 Feb 2;13(2):e1006187. doi: 10.1371/journal.ppat.1006187 (PMC5308826; doi:10.1371/journal.ppat.1006187)

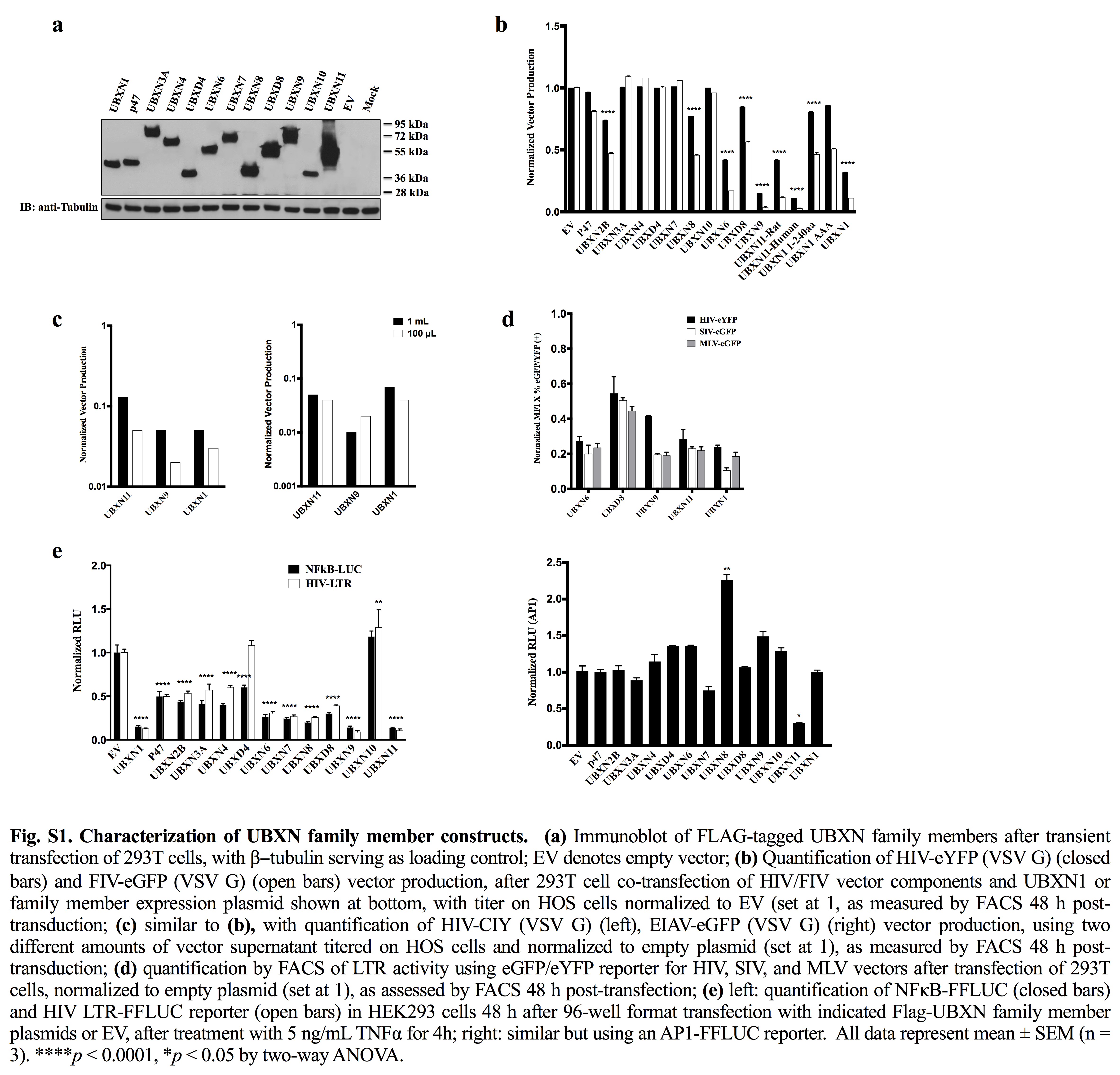

Supplement: S1 Fig — (a) Immunoblot of FLAG-tagged UBXN family members after transient transfection of 293T cells, with β−tubulin serving as loading control; EV denotes empty vector; (b) Quantification of HIV-eYFP (VSV G) (closed bars) and FIV-eGFP (VSV G) (open bars) vector production, after 293T cell co-transfection of HIV/FIV vector components and UBXN1 or family member expression plasmid shown at bottom, with titer on HOS cells normalized to EV (set at 1, as measured by FACS 48 h post-transduction; (c) similar to (b), with quantification of HIV-CIY (VSV G) (left), EIAV-eGFP (VSV G) (right) vector production, using two different amounts of vector supernatant titered on HOS cells and normalized to empty plasmid (set at 1), as measured by FACS 48 h post-transduction; (d) quantification by FACS of LTR activity using eGFP/eYFP reporter for HIV, SIV, and MLV vectors after transfection of 293T cells, normalized to empty plasmid (set at 1), as assessed by FACS 48 h post-transfection; (e) left: quantification of NFκB-FFLUC (closed bars) and HIV LTR-FFLUC reporter (open bars) in HEK293 cells 48 h after 96-well format transfection with indicated Flag-UBXN family member plasmids or EV, after treatment with 5 ng/mL TNFα for 4h; right: similar but using an AP1-FFLUC reporter. All data represent mean ± SEM (n = 3). ****p < 0.0001, *p < 0.05 by two-way ANOVA. (TIFF) [file ppat.1006187.s001.tiff]

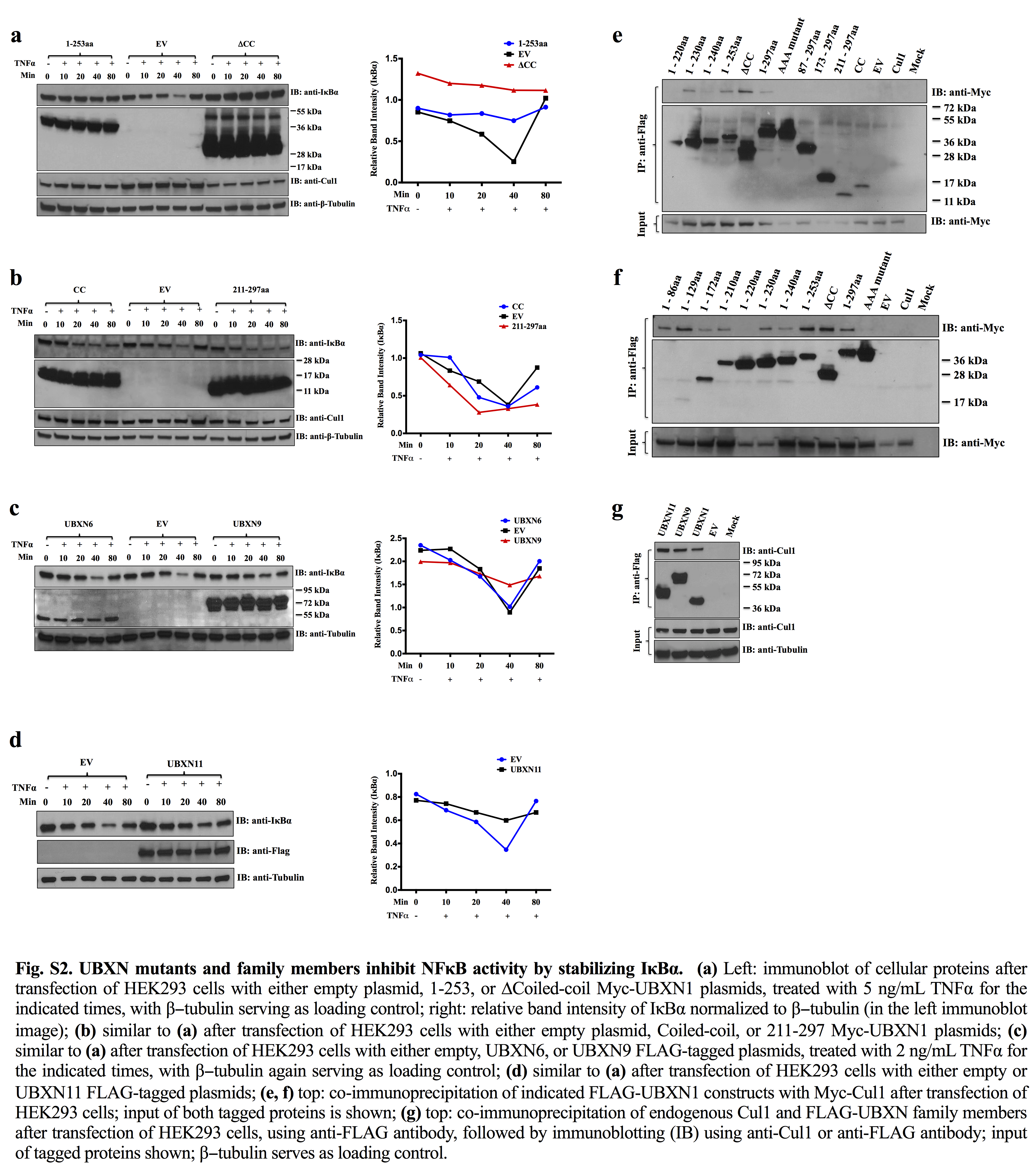

Supplement: S2 Fig — (a) Left: immunoblot of cellular proteins after transfection of HEK293 cells with either empty plasmid, 1–253, or ΔCoiled-coil Myc-UBXN1 plasmids, treated with 5 ng/mL TNFα for the indicated times, with β−tubulin serving as loading control; right: relative band intensity of IκBα normalized to β−tubulin (in the left immunoblot image); (b) similar to (a) after transfection of HEK293 cells with either empty plasmid, Coiled-coil, or 211–297 Myc-UBXN1 plasmids; (c) similar to (a) after transfection of HEK293 cells with either empty, UBXN6, or UBXN9 FLAG-tagged plasmids, treated with 2 ng/mL TNFα for the indicated times, with β−tubulin again serving as loading control; (d) similar to (a) after transfection of HEK293 cells with either empty or UBXN11 FLAG-tagged plasmids; (e, f) top: co-immunoprecipitation of indicated FLAG-UBXN1 constructs with Myc-Cul1 after transfection of HEK293 cells; input of both tagged proteins is shown; (g) top: co-immunoprecipitation of endogenous Cul1 and FLAG-UBXN family members after transfection of HEK293 cells, using anti-FLAG antibody, followed by immunoblotting (IB) using anti-Cul1 or anti-FLAG antibody; input of tagged proteins shown; β−tubulin serves as loading control. (TIFF) [file ppat.1006187.s002.tiff]

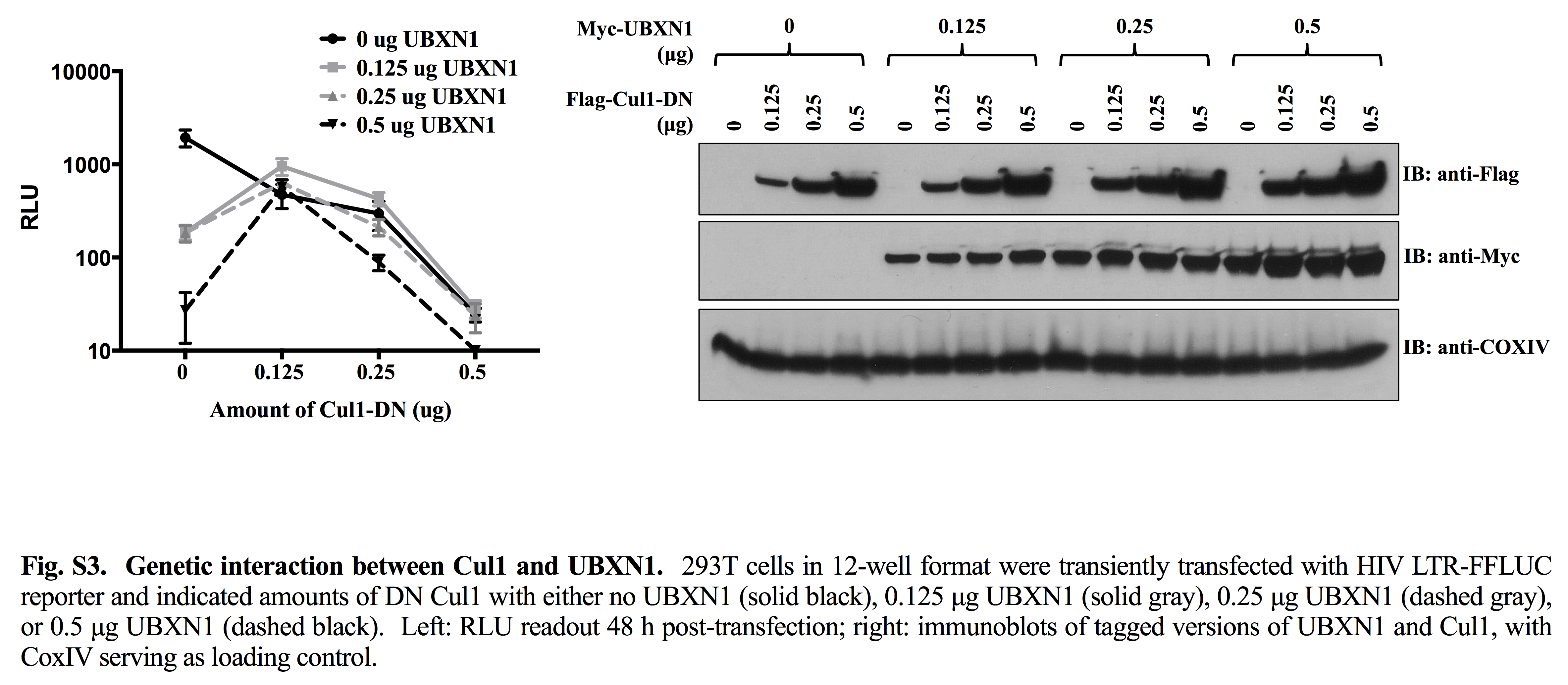

Supplement: S3 Fig — 293T cells in 12-well format were transiently transfected with HIV LTR-FFLUC reporter and indicated amounts of DN Cul1 with either no UBXN1 (solid black), 0.125 μg UBXN1 (solid gray), 0.25 μg UBXN1 (dashed gray), or 0.5 μg UBXN1 (dashed black). Left: RLU readout 48 h post-transfection; right: immunoblots of tagged versions of UBXN1 and Cul1, with CoxIV serving as loading control. (TIFF) [file ppat.1006187.s003.tiff]

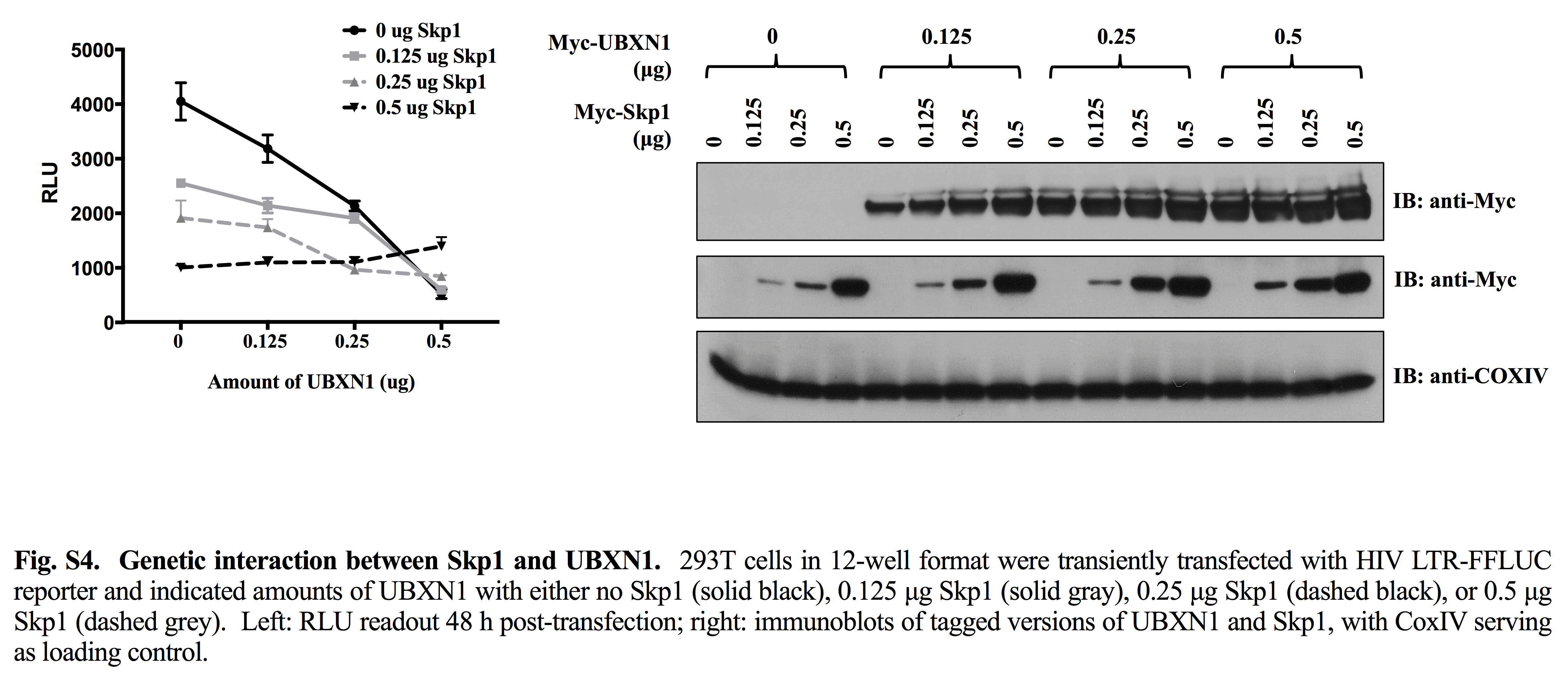

Supplement: S4 Fig — 293T cells in 12-well format were transiently transfected with HIV LTR-FFLUC reporter and indicated amounts of UBXN1 with either no Skp1 (solid black), 0.125 μg Skp1 (solid gray), 0.25 μg Skp1 (dashed black), or 0.5 μg Skp1 (dashed grey). Left: RLU readout 48 h post-transfection; right: immunoblots of tagged versions of UBXN1 and Skp1, with CoxIV serving as loading control. (TIFF) [file ppat.1006187.s004.tiff]

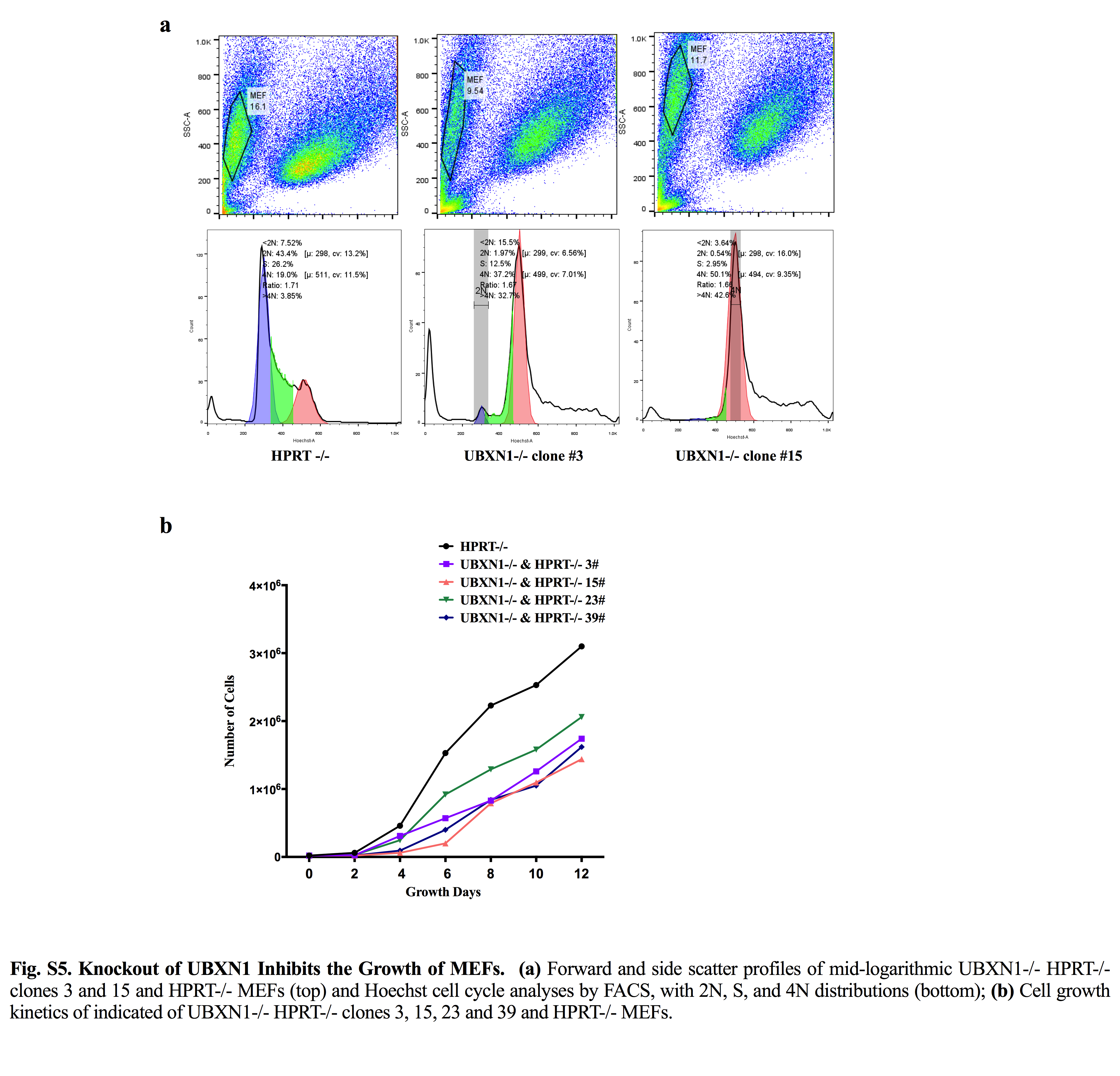

Supplement: S5 Fig — (a) Forward and side scatter profiles of mid-logarithmic UBXN1-/- HPRT-/- clones 3 and 15 and HPRT-/- MEFs (top) and Hoechst cell cycle analyses by FACS, with 2N, S, and 4N distributions (bottom); (b) Cell growth kinetics of indicated of UBXN1-/- HPRT-/- clones 3, 15, 23 and 39 and HPRT-/- MEFs. (TIFF) [file ppat.1006187.s005.tiff]

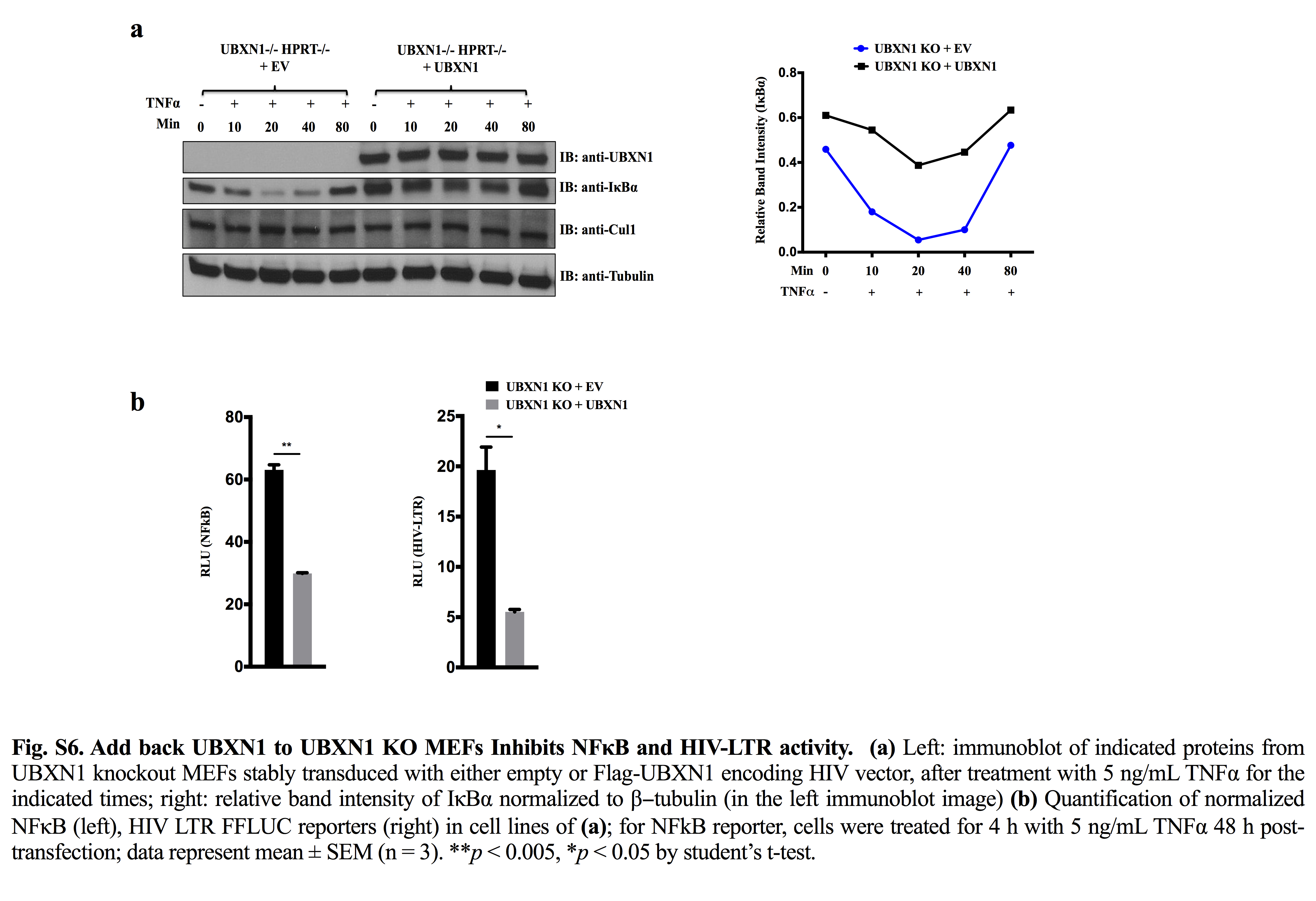

Supplement: S6 Fig — (a) Left: immunoblot of indicated proteins from UBXN1 knockout MEFs stably transduced with either empty or Flag-UBXN1 encoding HIV vector, after treatment with 5 ng/mL TNFα for the indicated times; right: relative band intensity of IκBα normalized to β−tubulin (in the left immunoblot image) (b) Quantification of normalized NFκB (left) and HIV LTR (right) FFLUC reporters in cell lines of (a); for NFkB reporter, cells were treated for 4 h with 5 ng/mL TNFα 48 h post-transfection; data represent mean ± SEM (n = 3). **p < 0.005, *p < 0.05 by student’s t-test. (TIFF) [file ppat.1006187.s006.tiff]

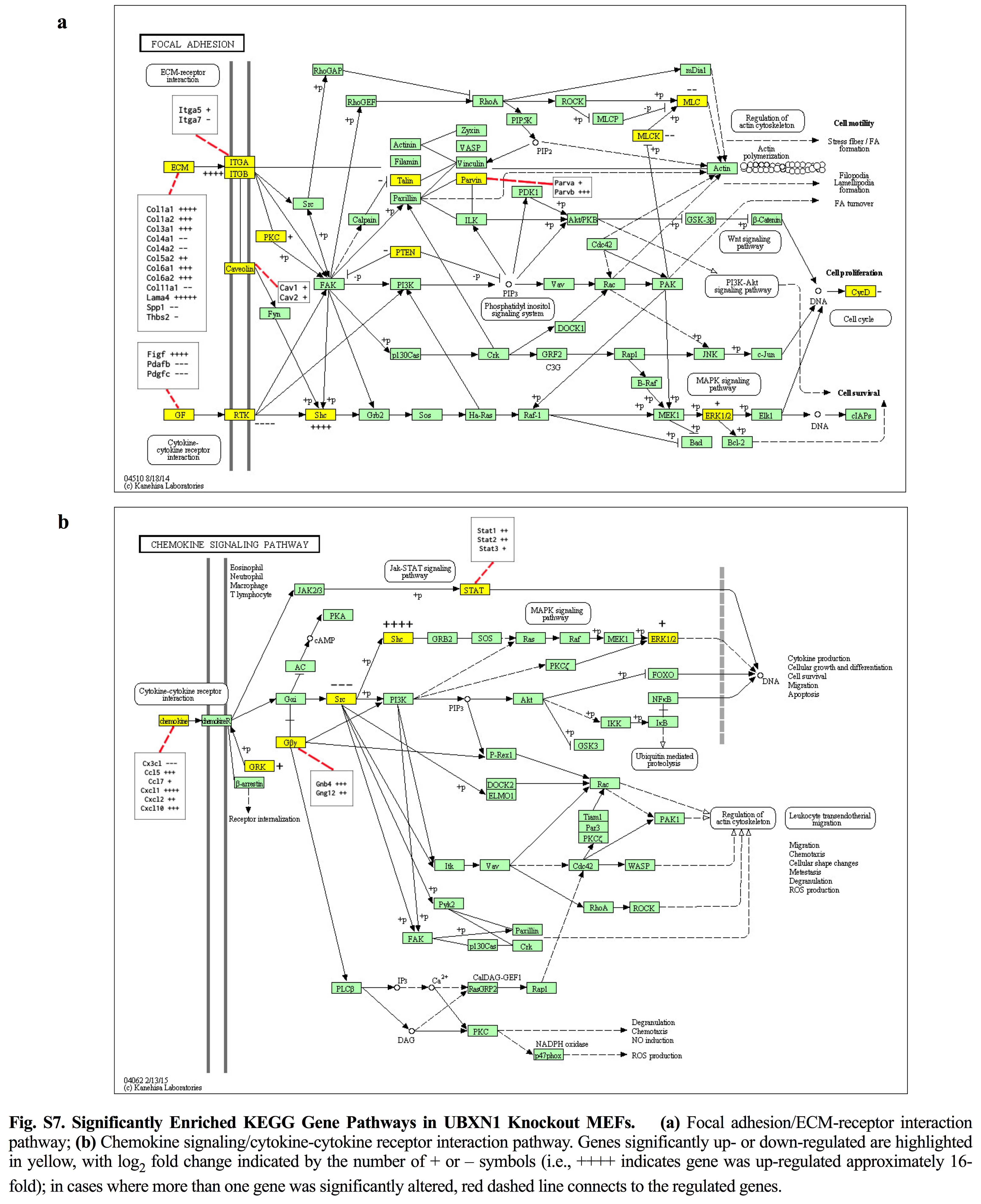

Supplement: S7 Fig — (a) Focal adhesion/ECM-receptor interaction pathway; (b) Chemokine signaling/cytokine-cytokine receptor interaction pathway. Genes significantly up- or down-regulated are highlighted in yellow, with log2 fold change indicated by the number of + or – symbols (i.e., ++++ indicates gene was up-regulated approximately 16-fold); in cases where more than one gene was significantly altered, red dashed line connects to the regulated genes. (TIFF) [file ppat.1006187.s007.tiff]

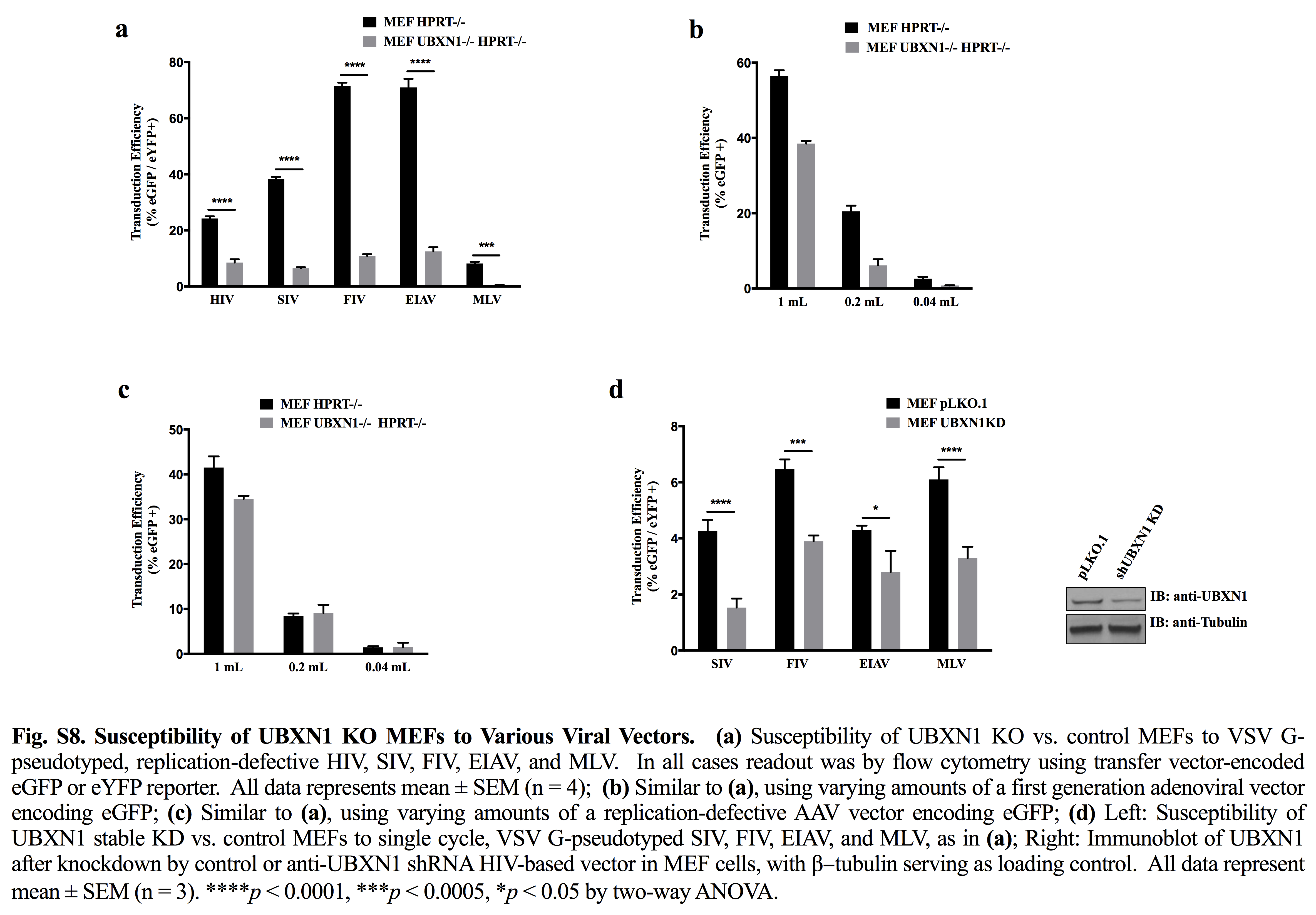

Supplement: S8 Fig — (a) Susceptibility of UBXN1 KO vs. control MEFs to VSV G-pseudotyped, replication-defective HIV, SIV, FIV, EIAV, and MLV. In all cases readout was by flow cytometry using transfer vector-encoded eGFP or eYFP reporter. All data represents mean ± SEM (n = 4); (b) Similar to (a), using varying amounts of a first generation adenoviral vector encoding eGFP; (c) Similar to (a), using varying amounts of a replication-defective AAV vector encoding eGFP; (d) Left: Susceptibility of UBXN1 stable KD vs. control MEFs to single cycle, VSV G-pseudotyped SIV, FIV, EIAV, and MLV, as in (a); Right: Immunoblot of UBXN1 after knockdown by control or anti-UBXN1 shRNA HIV-based vector in MEF cells, with β-tubulin serving as loading control. All data represent mean ± SEM (n = 3). ****p < 0.0001, ***p < 0.0005, *p < 0.05 by two-way ANOVA. (TIFF) [file ppat.1006187.s008.tiff]

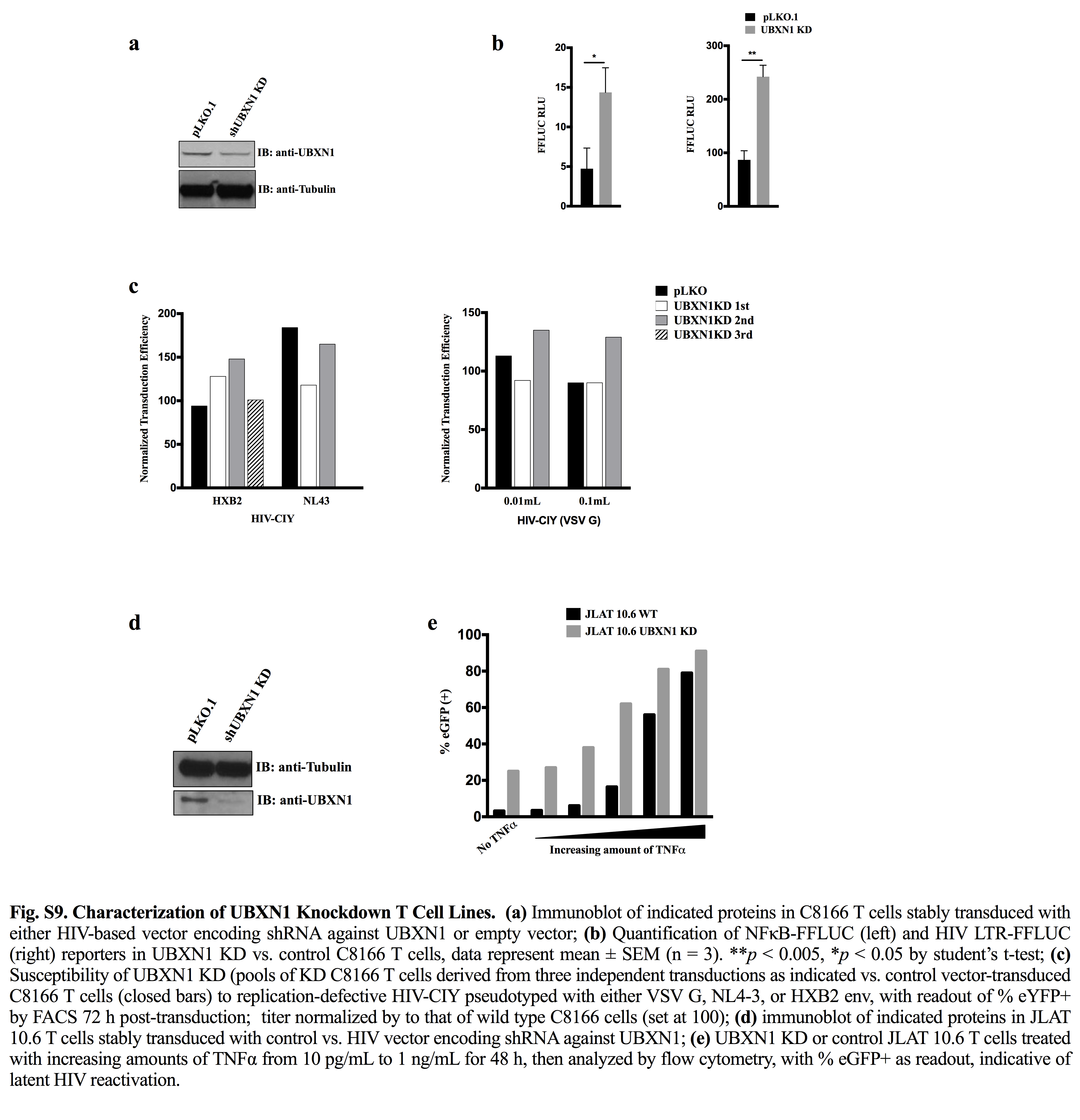

Supplement: S9 Fig — (a) Immunoblot of indicated proteins in C8166 T cells stably transduced with either HIV-based vector encoding shRNA against UBXN1 or empty vector; (b) Quantification of NFκB-FFLUC (left) and HIV LTR-FFLUC (right) reporters in UBXN1 KD vs. control C8166 T cells, data represent mean ± SEM (n = 3). **p < 0.005, *p < 0.05 by student’s t-test; (c) Susceptibility of UBXN1 KD (pools of KD C8166 T cells derived from three independent transductions as indicated vs. control vector-transduced C8166 T cells (closed bars) to replication-defective HIV-CIY pseudotyped with either VSV G, NL4-3, or HXB2 env, with readout of % eYFP+ by FACS 72 h post-transduction; titer normalized by to that of wild type C8166 cells (set at 100); (d) Immunoblot of indicated proteins in JLAT 10.6 T cells stably transduced with control vs. HIV vector encoding shRNA against UBXN1; (e) UBXN1 KD or control JLAT 10.6 T cells treated with increasing amounts of TNFα from 10 pg/mL to 1 ng/mL for 48 h, then analyzed by flow cytometry, with % eGFP+ as readout, indicative of latent HIV reactivation. (TIFF) [file ppat.1006187.s009.tiff]
