# Supplementary material for: Multiple UBXN family members inhibit retrovirus and lentivirus production and canonical NFκΒ signaling by stabilizing IκBα
Source: PLoS Pathog. 2017 Feb 2;13(2):e1006187. doi: 10.1371/journal.ppat.1006187 (PMC5308826; doi:10.1371/journal.ppat.1006187)
Supplement: S1 Table — (PDF) [file ppat.1006187.s010.pdf]

Supplementary Table 1: Primers for Amplifying Portions of Cul1[1]

Primers	Sequence
Cul1 0-F	5’ --- CCGCGGCCGCCATGTCGTCAACCCGGAGCCAG --- 3’
Cul1 1-R	5’ --- CCGGATCCTTAAGCCAAGTAACTGTAGGTGTCC --- 3’
Cul1 1/3-R	5’ --- CCGGATCCCTACTCAAGCAGACGAGCCTCTGC --- 3’
Cul1 1/3-F	5’ --- CCGCGGCCGCCGAACAACGAAGAGTTCAGGTTTACC --- 3’
Cul1 2/3-R	5’ --- CCGGATCCCTAGCTCACGCCAATGTCTTGAAACATG --- 3’
Cul1 2/3-F	5’ --- CCGCGGCCGCCGTGAGCAAAGATCTGAACGAGCAATTC --- 3’
Cul1 1/2-R	5’ --- CCGGATCCCTAGCCAGCGTCATTGTTGAATGCAGAC --- 3’
Cul1 1/2-F	5’ --- CCGCGGCCGCCGGCTTTGTGGCTGCTCTTGATAAG --- 3’

Footnote: [1]Full-length Cul1 expression plasmid used as template for PCR amplification; sequence of all PCR products confirmed and cloned into pcDNA3-myc expression vector in frame.
