# Supplementary material for: Multiple UBXN family members inhibit retrovirus and lentivirus production and canonical NFκΒ signaling by stabilizing IκBα
Source: PLoS Pathog. 2017 Feb 2;13(2):e1006187. doi: 10.1371/journal.ppat.1006187 (PMC5308826; doi:10.1371/journal.ppat.1006187)
Supplement: S2 Table — (PDF) [file ppat.1006187.s011.pdf]

Supplementary Table 2: Screening of UBXN1 Heterozygous Matings[1]

Mating Date	Fetus Age	Fetus Number	Heterozygous	Wild Type	Homozygous
5/9/2014	7.5	13	13	0	0
3/6/2014	10	12	9	3	0
3/4/2014	18	5	5	0	0
2/27/2014	16	8	7	1	0
2/20/2014	12	9	4	5	0

Footnote: [1]Genotyping performed by PCR using primers spanning the inserted intronic LoxP site.
