# Supplementary material for: Multiple UBXN family members inhibit retrovirus and lentivirus production and canonical NFκΒ signaling by stabilizing IκBα
Source: PLoS Pathog. 2017 Feb 2;13(2):e1006187. doi: 10.1371/journal.ppat.1006187 (PMC5308826; doi:10.1371/journal.ppat.1006187)
Supplement: S3 Table — (PDF) [file ppat.1006187.s012.pdf]

Supplementary Table 3: Genes Significantly Up- and Down-Regulated in UBXN1 Knockout MEFs

Gene ID	Gene Name	Gene Description	Locus	Control[1]	KnockOut	Fold-change (log10)[2]	q value[3]
NM_001285435	Adamtsl5	ADAMTS-like protein 5 precursor	chr10:79697304-80369637	0	1.42807	5000	0.016073
NM_009484	Uty	histone demethylase UTY	chrY:1097143-1245738	0	2.85296	5000	0.002524
NM_012011	Eif2s3y	eukaryotic translation initiation factor 2 subunit 3, Y-linked	chrY:1010611-1028598	0	8.70761	5000	0.002524
NM_031191	Prl2c2	prolactin-2C2 precursor	chr13:12996123-13005330	0	5.54222	5000	0.002524
NM_009712	Arsb	arylsulfatase B precursor	chr13:93771678-93943016	0.196597	27.2401	2.14163	0.002524
NM_007542	Bgn	biglycan precursor	chrX:73483634-73495936	1.02368	137.017	2.126611	0.002524
NM_016873	Wisp2	WNT1-inducible-signaling pathway protein 2 precursor	chr2:163820833-163833147	1.00491	92.0165	1.961737	0.038016
NM_010180	Fbln1	fibulin-1 precursor	chr15:85206007-85286294	0.548704	36.2794	1.820322	0.023435
NM_183319	Xkrx	XK-related protein 2	chrX:134149044-134161928	0.036912	2.35086	1.804064	0.002524
NM_028572	Vgll3	transcription cofactor vestigial-like protein 3	chr16:65815632-65863066	0.505694	29.7697	1.769885	0.031798
NM_133187	Fam198b	protein FAM198B	chr3:79885929-79946278	0.733831	41.1302	1.748566	0.002524
NM_009621	Adamts1	A disintegrin and metalloproteinase with thrombospondin motifs 1 precursor	chr16:85793827-85803115	2.37686	111.894	1.672806	0.002524
NM_009099	Trim30a	tripartite motif-containing protein 30A	chr7:104409025-104465193	0.351146	15.095	1.633347	0.014951

NM_054041	Antxr1	anthrax toxin receptor 1 precursor	chr6:87133852-87335775	0.594806	22.9428	1.586272	0.002524
NM_053110	Gpnmb	transmembrane glycoprotein NMB precursor	chr6:49036517-49058182	1.41592	51.8154	1.56342	0.002524
NM_011782	Adamts5	A disintegrin and metalloproteinase with thrombospondin motifs 5 precursor	chr16:85858156-85901125	0.444932	15.6567	1.546406	0.006356
NM_021384	Rsad2	radical S-adenosyl methionine domain-containing protein 2	chr12:26442742-26456452	0.475216	15.4142	1.511029	0.002524
NM_010681	Lama4	laminin subunit alpha-4 precursor	chr10:38965514-39110188	0.889325	27.4709	1.489813	0.002524
NM_172964	Arhgap28	rho GTPase-activating protein 28	chr17:67842707-68004108	0.386354	11.7481	1.482985	0.013662
NM_001033217	Prickle1	prickle-like protein 1	chr15:93499113-93595891	0.432469	13.1109	1.481676	0.012409
NM_001271419	Ly6a	lymphocyte antigen 6A-2/6E-1 precursor	chr15:74994876-74998031	7.84168	235.744	1.47803	0.002524
NM_008987	Ptx3	pentraxin-related protein PTX3 precursor	chr3:66053557-66296837	12.3801	357	1.459947	0.002524
NM_011854	Oasl2	2'-5'-oligoadenylate synthase-like protein 2	chr5:114896933-114912245	1.00376	28.8389	1.458346	0.002524
NM_009141	Cxcl5	C-X-C motif chemokine 5 precursor	chr5:90759297-90761625	1.16375	33.2504	1.455938	0.017468
NM_011454	Serpinb6b	serine (or cysteine) proteinase inhibitor, clade B, member 6b	chr13:32965512-32979037	0.610599	16.7462	1.438162	0.018718
NM_001252055	Ly6c1	lymphocyte antigen 6C1 isoform 1 precursor	chr15:75044017-75048837	4.16606	107.111	1.410109	0.004567

NM_011909	Usp18	ubl carboxyl-terminal hydrolase 18	chr6:121245905-121270917	1.62296	41.1367	1.403923	0.006356
NM_001113379	Lrrc32	leucine-rich repeat-containing protein 32 precursor	chr7:98494221-98501830	1.06628	25.8434	1.384479	0.002524
NM_001293783	Ddx60	probable ATP-dependent RNA helicase DDX60	chr8:61928086-62037701	0.288614	6.85139	1.37546	0.012409
NM_007472	Aqp1	aquaporin-1	chr6:55336298-55348555	8.69906	203.413	1.368904	0.002524
NM_031167	Il1rn	interleukin-1 receptor antagonist protein isoform 1	chr2:24336859-24351491	1.29094	29.2328	1.354963	0.011083
NM_133186	Steap3	metalloreductase STEAP3	chr1:120226415-120271082	1.75776	36.7175	1.319914	0.002524
NM_177378	Rnf150	RING finger protein 150 precursor	chr8:82863355-83091271	0.32921	6.58855	1.301317	0.002524
NM_011150	Lgals3bp	galectin-3-binding protein precursor	chr11:118392751-118401931	1.88991	36.127	1.281388	0.012409
NM_007742	Col1a1	collagen alpha-1(I) chain precursor	chr11:94936269-94951856	14.7984	279.845	1.276701	0.002524
NM_009061	Rgs2	regulator of G-protein signaling 2	chr1:143999337-144004149	0.690824	13.0295	1.27556	0.012409
NM_001302649	Ifi203	interferon-activable protein 203 isoform 1	chr1:173920400-173942672	0.367948	6.73826	1.262761	0.039854
NM_008091	Gata3	trans-acting T-cell-specific transcription factor GATA-3	chr2:9857077-9878600	0.531315	9.44703	1.249943	0.029348
NM_008176	Cxcl1	growth-regulated alpha protein precursor	chr5:90891244-90893115	24.7433	428.438	1.238431	0.002524

NM_181404	Kank1	KN motif and ankyrin repeat domain-containing protein 1	chr19:25237201-25434496	0.458739	7.77849	1.229331	0.024582
NM_008092	Gata4	transcription factor GATA-4	chr14:63198914-63245260	0.498985	8.16442	1.213837	0.028592
NM_172647	F11r	junctional adhesion molecule A precursor	chr1:171437560-171464593	2.12416	34.099	1.205556	0.002524
NM_033314	Slco2a1	solute carrier organic anion transporter family member 2A1	chr9:103008488-103087849	0.650205	10.4308	1.20527	0.013662
NM_025779	Ccdc109b	calcium uniporter regulatory subunit MCUb, mitochondrial isoform1	chr3:129914959-129970206	2.5903	41.3602	1.203235	0.012409
NM_010260	Gbp2	interferon-induced guanylate-binding protein 2	chr3:142620662-142638008	3.43212	54.4946	1.200791	0.002524
NM_019440	Irgm2	interferon inducible GTPase 2	chr11:58214976-58222783	1.29652	19.9406	1.186958	0.002524
NM_001164806	Bend4	BEN domain-containing protein 4	chr5:67392146-67427799	0.447754	6.86619	1.185676	0.012409
NM_010216	Figf	vascular endothelial growth factor D isoform VEGF-D358 preproprotein	chrX:164373547-164402647	1.36282	20.6511	1.180504	0.029348
NM_001285995	Enpp2	ectonucleotide pyrophosphatase/phosphodiesterase family member 2 isoform 4 preproprotein	chr15:54838678-54920146	1.44637	21.8289	1.178752	0.002524

NM_175549	Robo2	roundabout homolog 2 precursor	chr16:73892305-74410912	0.583466	8.78952	1.177951	0.002524
NM_001083312	Gbp7	guanylate binding protein 7	chr3:142530335-142550151	1.38918	20.7451	1.174155	0.002524
NM_020259	Hhip	hedgehog-interacting protein precursor	chr8:79965850-80058008	0.18051	2.67697	1.171142	0.0266
NM_173006	Pon3	serum paraoxonase/lactonase 3 precursor	chr6:5220851-5256233	1.2121	17.7565	1.165817	0.009547
NM_172443	Tbc1d16	TBC1 domain family member 16	chr11:119143042-119228499	0.173979	2.53919	1.164197	0.040429
NM_172463	Sned1	sushi, nidogen and EGF-like domain-containing protein 1 precursor	chr1:93235896-93296448	1.35577	19.7707	1.163833	0.002524
NM_009778	C3	complement C3 preproprotein	chr17:57203966-57228136	1.92884	27.7235	1.157554	0.002524
NM_001162884	Igsf10	immunoglobulin superfamily member 10 precursor	chr3:59006977-59344256	1.98089	28.2407	1.154014	0.002524
NM_001005858	Ifit3b	interferon-induced protein with tetratricopeptide repeats 3-like	chr19:34607956-34613401	0.927448	13.2124	1.153691	0.038016
NM_009472	Unc5c	netrin receptor UNC5C isoform 2 precursor	chr3:141465563-141834924	0.407322	5.7674	1.151042	0.017468
NM_010930	Nov	protein NOV homolog precursor	chr15:54745927-54753761	7.03302	98.9731	1.148375	0.002524
NM_022018	Fam129a	protein Niban	chr1:151571372-151719347	1.6931	22.9795	1.132658	0.002524
NM_013566	Itgb7	integrin beta-7 precursor	chr15:102215994-102231935	0.908426	12.3072	1.13187	0.042004

NM_001291857	Aebp1	adipocyte enhancer-binding protein 1 isoform 1 precursor	chr11:5861865-5878256	8.28846	110.752	1.125876	0.002524
NM_001024539	Shc2	SHC-transforming protein 2	chr10:79617937-79637918	0.396447	5.24991	1.121966	0.031798
NM_009636	Aebp1	adipocyte enhancer-binding protein 1 isoform 2 precursor	chr11:5861865-5878256	4.90136	64.508	1.119299	0.004567
NM_009180	St6galnac2	alpha-N-acetylgalactosaminide alpha-2,6-sialyltransferase 2	chr11:116676704-116694660	1.12119	14.7063	1.117827	0.032599
NM_175459	Glis3	zinc finger protein GLIS3 isoform 1	chr19:28258850-28720027	1.07199	13.2652	1.092519	0.002524
NM_018734	Gbp3	guanylate-binding protein 4	chr3:142560051-142573212	1.50197	18.223	1.083961	0.029348
NM_010501	Ifit3	interferon-induced protein with tetratricopeptide repeats 3	chr19:34583528-34588982	3.25829	38.7776	1.075592	0.002524
NM_028331	C1qtnf6	complement C1q tumor necrosis factor-related protein 6 isoform 1 precursor	chr15:78523345-78529651	1.13691	13.357	1.069984	0.012409
NM_010720	Lipg	endothelial lipase precursor	chr18:74939321-74961263	2.01806	22.9048	1.054993	0.002524
NM_001243008	Col6a3	collagen alpha-3(VI) chain isoform 1 precursor	chr1:90766859-90843971	0.385138	4.35128	1.053	0.013662
NM_013598	Kitl	kit ligand precursor	chr10:100015823-100100412	2.08231	23.4091	1.050839	0.002524

NM_133871	Ifi44	interferon-induced protein 44	chr3:151730922-151749959	1.02082	11.1639	1.038867	0.009547
NM_133362	Erdr1	erythroid differentiation regulator 1	chrY:90785441-90816465	29.2466	312.651	1.028984	0.002524
NM_011718	Wnt10b	protein Wnt-10b precursor	chr15:98771751-98778150	2.16775	23.078	1.027187	0.028592
NM_011019	Osmr	oncostatin-M-specific receptor subunit beta precursor	chr15:6813576-6874313	5.24898	54.1342	1.013396	0.002524
NM_010181	Fbn2	fibrillin-2 precursor	chr18:58008622-58209926	5.90268	60.5491	1.011057	0.002524
NM_023386	Rtp4	receptor-transporting protein 4	chr16:23609918-23614222	3.02056	30.4115	1.002951	0.002524
NM_026825	Lrrc16a	leucine-rich repeat-containing protein 16A	chr13:24012483-24280790	1.06615	10.3683	0.987887	0.002524
NM_001289493	Gbp3	guanylate-binding protein 4	chr3:142560051-142573212	2.58887	25.1309	0.987098	0.004567
NR_003508	Mx2	N/A	chr16:97536080-97560901	0.72792	7.02919	0.984823	0.048747
NM_175263	Notum	palmitoleoyl-protein carboxylesterase NOTUM precursor	chr11:120653788-120660837	4.28774	39.4949	0.964313	0.002524
NM_146007	Col6a2	collagen alpha-2(VI) chain precursor	chr10:76595755-76623404	6.16474	56.7517	0.964064	0.002524
NM_008331	Ifit1	interferon-induced protein with tetratricopeptide repeats 1	chr19:34640888-34650009	10.4299	95.0695	0.959762	0.002524
NM_011578	Tgfbr3	transforming growth factor beta receptor type 3 precursor	chr5:107106569-107289595	5.41063	49.1095	0.95792	0.002524

NM_153790	Scarf2	scavenger receptor class F member 2 precursor	chr16:17797281-17808287	7.35612	66.4538	0.95587	0.002524
NM_013584	Lifr	leukemia inhibitory factor receptor isoform 1 precursor	chr15:7129571-7197489	1.35007	12.0965	0.952302	0.002524
NM_021377	Sorcs1	VPS10 domain-containing receptor SorCS1 isoform 1 precursor	chr19:50143300-50678646	0.43003	3.83305	0.950048	0.023435
NM_133859	Olfml3	olfactomedin-like protein 3 precursor	chr3:103735393-103738001	5.46799	47.4854	0.938732	0.002524
NM_007609	Casp4	caspase-4 precursor	chr9:5308848-5336791	1.35333	11.7398	0.938256	0.04259
NM_133167	Parvb	beta-parvin	chr15:84232042-84315609	0.564773	4.87656	0.936239	0.032599
NM_031397	Bicc1	protein bicaudal C homolog 1	chr10:70925095-71159634	0.75103	6.45001	0.933903	0.043282
NM_010207	Fgfr2	fibroblast growth factor receptor 2 isoform IIIc	chr7:130162450-130266808	1.07054	9.1755	0.933024	0.002524
NM_020557	Cmpk2	UMP-CMP kinase 2, mitochondrial precursor	chr12:26469214-26479837	0.676461	5.75398	0.929725	0.030873
NM_019990	Stard10	PCTP-like protein	chr7:101321318-101346312	3.76024	31.894	0.928494	0.009547
NM_145515	Mark1	serine/threonine-protein kinase MARK1	chr1:184896423-184999549	1.05392	8.93086	0.928088	0.011083
NM_023738	Uba7	ubiquitin-like modifier-activating enzyme 7	chr9:107975566-107984056	0.960637	8.01882	0.921552	0.029348
NM_009369	Tgfbi	transforming growth factor-beta-induced protein ig-h3 precursor	chr13:56609602-56639339	5.33033	44.2956	0.919608	0.002524
NM_001081351	Cped1	cadherin-like and PC-esterase domain-containing protein 1	chr6:21985909-22255606	0.48205	3.93085	0.911395	0.021334

NM_007963	Mecom	MDS1 and EVI1 complex locus protein EVI1 isoform 1	chr3:29951295-30013204	4.4493	35.3079	0.89958	0.002524
NM_015744	Enpp2	ectonucleotide pyrophosphatase/phosphodiesterase family member 2 isoform 2 preproprotein	chr15:54838678-54920146	4.66672	36.614	0.894634	0.002524
NM_007553	Bmp2	bone morphogenetic protein 2 preproprotein	chr2:133552158-133562896	1.43037	11.0383	0.887454	0.007984
NM_001201470	Papss2	bifunctional 3'-phosphoadenosine 5'-phosphosulfate synthase 2 isoform 2	chr19:32595714-32667187	0.89538	6.86921	0.884899	0.011083
NM_009644	Ahrr	aryl hydrocarbon receptor repressor	chr13:74211117-74292309	0.991954	7.60979	0.884881	0.006356
NM_009323	Tbx15	T-box transcription factor TBX15	chr3:99253759-99354260	8.04592	61.6444	0.884318	0.002524
NM_013653	Ccl5	C-C motif chemokine 5 precursor	chr11:83525778-83530518	20.6549	155.502	0.876714	0.006356
NM_001164100	Add3	gamma-adducin isoform b	chr19:53140442-53247326	2.67812	20.0928	0.875212	0.002524
NM_001033207	Nlrc5	protein NLRC5	chr8:94472762-94527272	0.844752	6.29804	0.872475	0.002524
NM_176848	Fbxo2	F-box only protein 2	chr4:148160667-148166417	4.56686	33.7853	0.86911	0.012409
NM_013846	Ror2	tyrosine-protein kinase transmembrane receptor ROR2 precursor	chr13:53109316-53286109	1.68946	12.4424	0.867156	0.013662
NM_009930	Col3a1	collagen alpha-1(III) chain precursor	chr1:45311537-45349706	20.3584	148.28	0.862337	0.002524
NR_030671	AW011738	N/A	chr4:156203283-156206028	0.382447	2.75646	0.857782	0.023435

NM_138741	Sdpr	serum deprivation-response protein	chr1:51218385-51333779	11.206	79.6453	0.85171	0.002524
NM_009846	Cd24a	signal transducer CD24 precursor	chr10:43579168-43584265	22.58	159.39	0.848739	0.002524
NM_007743	Col1a2	collagen alpha-2(I) chain precursor	chr6:4505696-4541543	2.84414	20.0751	0.848706	0.002524
NM_008086	Gas1	growth arrest-specific protein 1	chr13:60174404-60177535	18.6108	130.86	0.847041	0.002524
NM_008813	Enpp1	ectonucleotide pyrophosphatase/phosphodiesterase family member 1 isoform 2	chr10:24637913-24712159	1.24178	8.69058	0.845006	0.002524
NM_013683	Tap1	antigen peptide transporter 1 isoform 1	chr17:34187555-34197225	2.06648	14.3296	0.841003	0.011083
NM_011507	Suclg2	succinyl-CoA ligase [GDP-forming] subunit beta, mitochondrial precursor	chr6:95473008-95718846	3.25127	22.372	0.837652	0.002524
NM_001039530	Parp14	poly [ADP-ribose] polymerase 14	chr16:35832877-35871382	1.43878	9.88666	0.837053	0.002524
NM_008381	Inhbb	inhibin beta B chain precursor	chr1:119415464-119422248	7.73599	52.4375	0.831126	0.002524
NM_028882	Sema3d	semaphorin-3D precursor	chr5:12383165-12588943	2.45629	16.6077	0.83003	0.002524
NM_030150	Dhx58	probable ATP-dependent RNA helicase DHX58	chr11:100694883-100704271	1.05033	7.08431	0.82897	0.035191
NM_134163	Mbnl3	muscleblind-like protein 3	chrX:51113493-51205832	0.814479	5.458	0.826153	0.002524

NM_010156	Samd9l	sterile alpha motif domain-containing protein 9-like	chr6:3372257-3399571	5.22856	34.5877	0.820539	0.002524
NM_001033301	Fhdc1	FH2 domain-containing protein 1	chr3:84442195-84480439	2.02464	13.3847	0.820259	0.002524
NM_009933	Col6a1	collagen alpha-1(VI) chain precursor	chr10:76708791-76726044	24.2366	158.386	0.815246	0.002524
NM_001168346	Nfatc4	nuclear factor of activated T-cells, cytoplasmic 4 isoform 2	chr14:55824794-55833943	0.884858	5.72455	0.810866	0.041104
NM_145379	Mrgprf	mas-related G-protein coupled receptor member F	chr7:145300908-145309557	3.55861	22.8846	0.808263	0.004567
NM_175512	Dhrs9	dehydrogenase/reductase SDR family member 9 precursor	chr2:69380461-69403086	1.0601	6.71659	0.801802	0.041104
NM_181545	Slfn8	schlafen 8 isoform 1	chr11:83002157-83020810	0.434975	2.74884	0.800686	0.046295
NM_013531	Gnb4	guanine nucleotide-binding protein subunit beta-4	chr3:32583527-32616535	2.85872	18.0617	0.800586	0.007984
NM_175155	Sash1	SAM and SH3 domain-containing protein 1	chr10:8722218-8886070	0.50115	3.13259	0.795935	0.049461
NM_177632	Fam43a	protein FAM43A	chr16:30599722-30602797	2.1952	13.6827	0.794698	0.004567
NM_007603	Capn6	calpain-6	chrX:143802236-143827412	4.11925	25.4566	0.790983	0.002524
NM_001039485	Piezo2	piezo-type mechanosensitive ion channel component 2	chr18:63010212-63387183	0.328871	2.01181	0.786561	0.040429
NM_183162	Helz2	helicase with zinc finger domain 2	chr2:181227614-181242027	1.65708	10.1014	0.785038	0.002524
NM_021274	Cxcl10	C-X-C motif chemokine 10 precursor	chr5:92331840-92414627	22.453	135.867	0.781841	0.002524

NM_010288	Gja1	gap junction alpha-1 protein	chr10:56377299-56390419	36.5628	219.213	0.777828	0.002524
NM_008425	Kcnj2	inward rectifier potassium channel 2	chr11:111066163-111076825	1.04916	6.25613	0.775462	0.007984
NM_001081437	Fbln2	fibulin-2 isoform b precursor	chr6:91212763-91272540	50.2116	298.955	0.774803	0.002524
NM_015783	Isg15	ubiquitin-like protein ISG15 precursor	chr4:156199423-156200818	31.1361	185.015	0.773942	0.002524
NM_010517	Igfbp4	insulin-like growth factor-binding protein 4 precursor	chr11:99041259-99052643	15.7904	93.7747	0.773695	0.002524
NM_015819	Hs6st2	heparan-sulfate 6-O-sulfotransferase 2 isoform 2 precursor	chrX:51386636-51681602	6.68484	39.2374	0.768608	0.002524
NM_001159418	Irf9	interferon regulatory factor 9 isoform 2	chr14:55603984-55610030	5.02004	29.1136	0.763388	0.002524
NM_027835	Ifih1	interferon-induced helicase C domain-containing protein 1 isoform 1	chr2:62595792-62646255	2.45582	14.1486	0.760516	0.002524
NM_022814	Svep1	sushi, von Willebrand factor type A, EGF and pentraxin domain-containing protein 1 precursor	chr4:58042795-58206596	0.570242	3.25559	0.75657	0.016073
NM_144910	Cnot6l	CCR4-NOT transcription complex subunit 6-like isoform 1	chr5:96070332-96161990	1.48018	8.41912	0.754953	0.039854
NM_001083897	Mpzl1	myelin protein zero-like protein 1 isoform a precursor	chr1:165592180-165634541	3.54102	20.1048	0.75417	0.009547

NM_011824	Grem1	gremlin-1 precursor	chr2:113748674-113758648	48.3834	274.633	0.754056	0.002524
NM_001162943	Dchs1	protocadherin-16 precursor	chr7:105752988-105787550	0.413525	2.34384	0.753427	0.011083
NM_009155	Sepp1	selenoprotein P precursor	chr15:3270766-3280508	3.67483	20.6529	0.749745	0.006356
NM_008608	Mmp14	matrix metalloproteinase-14 precursor	chr14:54431603-54441258	45.4076	251.067	0.742662	0.002524
NM_019547	Rbm38	RNA-binding protein 38	chr2:173021901-173034731	1.95866	10.7213	0.738288	0.002524
NM_001024945	Qsox1	sulfhydryl oxidase 1 isoform a precursor	chr1:155778154-155812899	4.35671	23.8031	0.737475	0.011083
NM_177390	Myo1d	unconventional myosin-Id	chr11:80482126-80780025	1.17272	6.36331	0.734489	0.006356
NM_028696	Nabp1	SOSS complex subunit B2	chr1:51469487-51478399	2.30533	12.5053	0.73436	0.006356
NM_001159394	Nfkbiz	NF-kappa-B inhibitor zeta isoform a	chr16:55811376-55838641	2.74898	14.7907	0.730817	0.016073
NM_001081324	Neto2	neuropilin and tolloid-like protein 2	chr8:85636587-85691009	0.768882	4.10264	0.727204	0.025561
NM_183300	Zfyve9	zinc finger FYVE domain-containing protein 9	chr4:108639258-108781904	2.28834	12.1056	0.723465	0.002524
NM_019631	Tmem45a	transmembrane protein 45A	chr16:56805160-56886163	7.22579	37.6835	0.717267	0.002524
NM_001025577	Maf	transcription factor Maf	chr8:115703252-115706894	3.74796	19.5169	0.716614	0.002524
NM_009154	Sema5a	semaphorin-5A precursor	chr15:32244812-32696341	3.52898	18.201	0.712445	0.002524
NM_001037713	Xaf1	XIAP-associated factor 1 isoform 1	chr11:72301628-72313733	2.58924	13.344	0.712117	0.013662
NM_011812	Fbln5	fibulin-5 precursor	chr12:101746564-101819119	4.63274	23.8263	0.711219	0.002524
NM_009284	Stat6	signal transducer and transcription activator 6	chr10:127642985-127666703	4.54117	23.0099	0.704747	0.045466

NM_013863	Bag3	BAG family molecular chaperone regulator 3	chr7:128523582-128546979	2.9436	14.9027	0.704386	0.002524
NM_027854	Tmem248	transmembrane protein 248	chr5:130219743-130243765	5.46495	27.5149	0.701981	0.040429
NM_134087	Fam83h	protein FAM83H	chr15:76001091-76014336	2.13724	10.7088	0.699886	0.004567
NR_131212	Neat1	N/A	chr19:5824709-5845480	9.88147	49.0869	0.696144	0.002524
NM_025992	Herc6	E3 ISG15--protein ligase Herc6	chr6:57580991-57665136	3.18266	15.7634	0.694862	0.002524
NM_001002927	Penk	proenkephalin-A precursor	chr4:4133535-4138445	3.27749	16.1329	0.69217	0.030873
NM_018738	Igtp	interferon gamma induced GTPase	chr11:58199555-58207592	3.96626	19.4736	0.691063	0.004567
NM_010101	S1pr3	sphingosine 1-phosphate receptor 3	chr13:51408617-51422797	1.45589	7.12101	0.689413	0.027658
NM_021704	Cxcl12	stromal cell-derived factor 1 isoform alpha precursor	chr6:117168534-117181368	17.8734	87.2007	0.688314	0.004567
NM_028243	Prcp	lysosomal Pro-X carboxypeptidase precursor	chr7:92875252-92934581	2.97103	14.4368	0.686562	0.012409
NM_008329	Ifi204	interferon-activable protein 204	chr1:173747293-173766919	5.983	28.9475	0.684693	0.002524
NM_133733	Clmp	CXADR-like membrane protein precursor	chr9:40685963-40784046	2.72705	13.1445	0.683052	0.002524
NM_008984	Ptprm	receptor-type tyrosine-protein phosphatase mu precursor	chr17:66666847-67354459	0.999686	4.771	0.678744	0.031798
NM_001134480	Plcxd2	PI-PLC X domain-containing protein 2	chr16:45959260-46010413	1.27097	6.05394	0.677901	0.002524

NM_023065	Ifi30	gamma-interferon-inducible lysosomal thiol reductase precursor	chr8:70762772-70766663	13.2928	63.1414	0.676697	0.002524
NM_172621	Clic5	chloride intracellular channel protein 5	chr17:44188571-44280168	0.614524	2.91669	0.676351	0.023435
NM_001114332	Slc16a10	monocarboxylate transporter 10 isoform 1	chr10:40033534-40142254	2.55222	11.9821	0.671616	0.022426
NM_009538	Plagl1	zinc finger protein PLAGL1	chr10:13090787-13131695	1.03562	4.85561	0.671044	0.006356
NM_009546	Trim25	E3 ubiquitin/ISG15 ligase TRIM25	chr11:88999402-89020293	4.44373	20.7533	0.669337	0.002524
NM_028186	Nkd2	protein naked cuticle homolog 2	chr13:73819327-73847631	3.07909	14.2814	0.666348	0.002524
NM_001103157	Steap2	metalloreductase STEAP2 isoform 1	chr5:5664828-5694568	1.49727	6.91058	0.664214	0.040429
NM_013556	Hprt	hypoxanthine-guanine phosphoribosyltransferase	chrX:52988077-53021660	18.0077	82.9536	0.663377	0.002524
NM_177662	Ctso	cathepsin O precursor	chr3:81932615-81956725	2.0173	9.23752	0.660785	0.027658
NM_133955	Rhou	rho-related GTP-binding protein RhoU	chr8:123653928-123663880	10.2129	45.9334	0.652979	0.002524
NM_001252401	Tle2	transducin-like enhancer protein 2 isoform 2	chr10:81575286-81590845	3.04192	13.6652	0.652467	0.046295
NM_178377	Commd10	COMM domain-containing protein 10	chr18:46958875-47087994	3.46167	15.5205	0.651622	0.002524
NM_153789	Mylip	E3 ubiquitin-protein ligase MYLIP	chr13:45389741-45411940	2.43032	10.8394	0.64934	0.024582

NM_001164040	Ly6e	lymphocyte antigen 6E precursor	chr15:74955050-74959905	30.344	134.56	0.646844	0.002524
NM_138587	Fam3c	protein FAM3C precursor	chr6:22306521-22356081	12.7082	55.7068	0.641823	0.002524
NM_011173	Pros1	vitamin K-dependent protein S precursor	chr16:62854333-62929340	11.6792	50.8382	0.638777	0.002524
NM_022721	Fzd5	frizzled-5 precursor	chr1:64730557-64737750	1.05379	4.57807	0.637931	0.046295
NM_008362	Il1r1	interleukin-1 receptor type 1 isoform 1 precursor	chr1:40225079-40316177	3.61754	15.4801	0.631359	0.039854
NM_019963	Stat2	signal transducer and activator of transcription 2	chr10:128270575-128292849	5.25449	22.4728	0.631127	0.002524
NM_027464	Fam213a	redox-regulatory protein FAM213A	chr14:40993739-41013775	5.54645	23.6603	0.630005	0.002524
NM_139149	Fus	RNA-binding protein FUS	chr7:127967478-127982031	11.4958	49.005	0.629704	0.002524
NM_198113	Ssh3	protein phosphatase Slingshot homolog 3	chr19:4261668-4269172	4.13425	17.522	0.627187	0.002524
NM_144830	Tmem106a	transmembrane protein 106A	chr11:101582241-101591785	2.64114	11.067	0.622238	0.022426
NM_011756	Zfp36	tristetraprolin	chr7:28376783-28379228	5.39472	22.579	0.621735	0.004567
NM_027219	Cdc42ep1	cdc42 effector protein 1	chr15:78842646-78855529	8.99294	37.4905	0.620019	0.002524
NM_001177558	Gng12	guanine nucleotide-binding protein G(I)/G(S)/G(O) subunit gamma-12	chr6:66896396-67021361	7.52061	31.1225	0.616823	0.002524
NM_172796	Slfn9	schlafen 9	chr11:82980302-82991830	4.86564	19.9584	0.612984	0.002524
NM_183221	Fat4	protocadherin Fat 4 precursor	chr3:38886939-39011983	4.19534	17.0775	0.609658	0.002524

NM_001173477	Speg	striated muscle-specific serine/threonine-protein kinase isoform 4	chr1:75375296-75432306	7.94157	32.2374	0.608454	0.048088
NM_007737	Col5a2	collagen alpha-2(V) chain precursor	chr1:45374330-45503282	41.5537	167.773	0.606112	0.002524
NM_001168333	Tinagl1	tubulointerstitial nephritis antigen-like precursor	chr4:130165599-130175122	14.4223	57.9074	0.603698	0.002524
NM_172612	Rnd1	rho-related GTP-binding protein Rho6 precursor	chr15:98669204-98677461	5.12499	20.5581	0.603291	0.004567
NM_001111053	Dclk1	serine/threonine-protein kinase DCLK1 isoform 4	chr3:55242525-55539068	9.31316	37.2404	0.601919	0.002524
NM_178884	Obsl1	obscurin-like protein 1	chr1:75485824-75506452	1.55382	6.19862	0.600895	0.046989
NM_026698	Tmem129	E3 ubiquitin-protein ligase TM129	chr5:33653215-33657832	4.0486	16.008	0.597033	0.002524
NM_001111279	Wdfy1	WD repeat and FYVE domain-containing protein 1 isoform 1	chr1:79702261-79761769	3.88081	15.3284	0.596572	0.002524
NM_008606	Mmp11	stromelysin-3 precursor	chr10:75923221-75932502	8.15296	32.1367	0.595687	0.002524
NM_027992	Tmem106b	transmembrane protein 106B	chr6:13069758-13089269	23.8578	93.7868	0.594513	0.002524
NM_028032	Ppp2r2a	serine/threonine-protein phosphatase 2A 55 kDa regulatory subunit B alpha isoform isoform 1	chr14:67014055-67072471	2.94267	11.5087	0.592283	0.007984
NM_172777	Gbp9	guanylate binding protein family, member 9	chr5:105078393-105110292	1.32924	5.17526	0.590329	0.043961

NM_028450	Gulp1	PTB domain-containing engulfment adapter protein 1	chr1:44551670-44796836	14.4302	55.7146	0.586698	0.002524
NM_173451	Arsj	arylsulfatase J precursor	chr3:126363851-126440374	1.74014	6.67956	0.584164	0.043961
NM_019413	Robo1	roundabout homolog 1 precursor	chr16:72663148-73046100	2.73498	10.4499	0.582159	0.002524
NM_001098222	Bhlhb9	protein BHLHb9	chrX:135885850-135891081	6.39827	24.3274	0.580034	0.002524
NM_019969	Plag1	zinc finger protein PLAG1	chr4:3901157-3938405	1.43602	5.44164	0.578571	0.025561
NM_139001	Cspg4	chondroitin sulfate proteoglycan 4 precursor	chr9:56865103-56899870	3.05925	11.576	0.577944	0.002524
NM_008610	Mmp2	72 kDa type IV collagenase precursor	chr8:92827327-92853420	8.04671	30.3777	0.576936	0.002524
NM_025567	Cyc1	cytochrome c1, heme protein, mitochondrial	chr15:76343522-76345934	41.5455	155.739	0.573875	0.002524
NM_009283	Stat1	signal transducer and activator of transcription 1 isoform 2	chr1:52119437-52161865	8.38628	31.4114	0.573516	0.002524
NM_007987	Fas	tumor necrosis factor receptor superfamily member 6 isoform 1 precursor	chr19:34290658-34327770	4.44301	16.464	0.568859	0.029348
NM_025408	Acer3	alkaline ceramidase 3	chr7:98213659-98309527	3.93865	14.5476	0.567445	0.004567
NM_178051	Mterf4	transcription termination factor 4, mitochondrial	chr1:93299210-93305870	4.4343	16.338	0.566373	0.009547
NM_198625	Mtss1l	MTSS1-like protein	chr8:110721483-110741400	3.94289	14.519	0.566123	0.006356

NM_028651	Tmtc4	transmembrane and TPR repeat-containing protein 4	chr14:122918974-122983261	4.41054	16.2283	0.56578	0.009547
NM_033602	Peli2	E3 ubiquitin-protein ligase pellino homolog 2	chr14:48120868-48260883	1.5405	5.65239	0.56457	0.013662
NM_001048207	Gypc	glycophorin-C	chr18:32528319-32560034	3.10289	11.3164	0.561942	0.023435
NM_022420	Gprc5b	G-protein coupled receptor family C group 5 member B isoform 2 precursor	chr7:118842217-118995211	4.17851	15.124	0.558645	0.017468
NM_021460	Lipa	lysosomal acid lipase/cholesteryl ester hydrolase precursor	chr19:34492315-34527474	26.4076	95.2964	0.557348	0.002524
NM_008845	Pip4k2a	phosphatidylinositol 5-phosphate 4-kinase type-2 alpha	chr2:18842255-18998121	1.69834	6.10605	0.555734	0.042004
NM_011452	Serpinb9b	serine (or cysteine) proteinase inhibitor, clade B, member 9b	chr13:33027413-33040558	5.20555	18.6455	0.554109	0.025561
NM_001303033	Ubr3	E3 ubiquitin-protein ligase UBR3 isoform 3	chr2:69897245-70024010	4.23905	15.0762	0.551023	0.029348
NM_011804	Creg1	protein CREG1 precursor	chr1:165763779-165775304	3.84044	13.6508	0.550777	0.025561
NM_175274	Ttyh3	protein tweety homolog 3 isoform 2	chr5:140620576-140649031	6.77625	24.039	0.549928	0.002524
NM_009906	Tpp1	tripeptidyl-peptidase 1 precursor	chr7:105744846-105752207	9.71184	34.3094	0.548112	0.002524
NM_009976	Cst3	cystatin-C precursor	chr2:148871731-148875468	323.068	1134.43	0.545484	0.002524
NM_178600	Vkorc1	vitamin K epoxide reductase complex subunit 1 precursor	chr7:127893062-127895617	17.2281	59.3496	0.537182	0.03705

NM_181444	Gprc5a	retinoic acid-induced protein 3	chr6:135065661-135084708	6.3664	21.8453	0.535463	0.025561
NM_001291068	Polr2a	DNA-directed RNA polymerase II subunit RPB1	chr11:69733998-69758633	3.67464	12.4492	0.529924	0.002524
NM_009752	Glb1	beta-galactosidase preproprotein	chr9:114401077-114474379	7.32492	24.5676	0.525559	0.039854
NM_023143	C1ra	complement C1r-A subcomponent precursor	chr6:124512620-124523440	7.65175	25.6359	0.52509	0.002524
NM_153412	Phldb2	pleckstrin homology-like domain family B member 2 isoform 2	chr16:45746230-45844378	18.4224	61.5412	0.523819	0.002524
NM_008326	Irgm1	immunity-related GTPase family M protein 1	chr11:48865248-48871346	11.3549	37.7717	0.521983	0.002524
NM_133918	Emilin1	EMILIN-1 precursor	chr5:30913785-30921273	18.3989	61.203	0.52198	0.002524
NM_145930	AW549877	UPF0600 protein C5orf51 homolog	chr15:3982034-3995752	11.9578	39.5479	0.519472	0.002524
NM_010221	Fkbp10	peptidyl-prolyl cis-trans isomerase FKBP10 isoform 1 precursor	chr11:100415693-100441089	25.5534	84.3678	0.518729	0.002524
NM_010498	Ids	iduronate 2-sulfatase precursor	chrX:70343069-70365085	6.03595	19.8805	0.517681	0.002524
NM_133832	Rdh10	retinol dehydrogenase 10	chr1:16105881-16132550	6.86964	22.6043	0.517257	0.009547
NM_001163513	Dlg5	disks large homolog 5 isoform 1	chr14:24133952-24245920	4.67342	15.374	0.517151	0.004567
NM_010809	Mmp3	stromelysin-1 preproprotein	chr9:7445821-7455974	17.7171	58.2464	0.516878	0.002524

NM_008626	Mrc2	C-type mannose receptor 2 precursor	chr11:105292645-105351145	17.5284	57.5985	0.516667	0.002524
NM_001163575	Parp10	poly [ADP-ribose] polymerase 10	chr15:76232994-76243440	7.44667	24.4514	0.516342	0.002524
NM_026162	Plxdc2	plexin domain-containing protein 2 precursor	chr2:16356303-16755839	1.8348	6.01532	0.51567	0.022426
NM_212445	Kdelc2	KDEL motif-containing protein 2 precursor	chr9:53384022-53401867	5.4658	17.7984	0.512726	0.002524
NM_011204	Ptpn13	tyrosine-protein phosphatase non-receptor type 13	chr5:103425191-103598361	3.90879	12.6711	0.510773	0.002524
NM_010065	Dnm1	dynamin-1 isoform 1	chr2:32308470-32353329	8.55952	27.6974	0.50999	0.004567
NM_172145	Eva1b	protein eva-1 homolog B	chr4:126148002-126149874	18.0684	58.2601	0.508452	0.018718
NM_133964	Dohh	deoxyhypusine hydroxylase	chr10:81384427-81388352	14.1785	45.5146	0.506519	0.002524
NM_001143689	H2-Q4	histocompatibility 2, Q region locus 4 precursor	chr17:35379616-35384674	9.17808	28.9992	0.499635	0.021334
NM_021606	Nek6	serine/threonine-protein kinase Nek6	chr2:38511696-38587490	5.24594	16.3919	0.494806	0.016073
NM_025943	Dzip1	zinc finger protein DZIP1	chr14:118875519-118925470	3.73364	11.66	0.494568	0.006356
NM_026768	Mrps18a	28S ribosomal protein S18a, mitochondrial precursor	chr17:46111003-46128908	16.8714	52.4022	0.492199	0.002524
NM_026599	Cgnl1	cingulin-like protein 1	chr9:71626506-71771602	9.75727	30.2558	0.49148	0.002524
NM_025706	Tbc1d15	TBC1 domain family member 15	chr10:115197870-115251493	10.2975	31.8647	0.490577	0.002524
NM_001013371	Dtx3l	E3 ubiquitin-protein ligase DTX3L	chr16:35926514-35972621	4.78364	14.5466	0.483003	0.041104

NM_009656	Aldh2	aldehyde dehydrogenase, mitochondrial isoform 1 precursor	chr5:121566026-121593824	14.213	42.9383	0.480158	0.014951
NM_172989	Lpar1	lysophosphatidic acid receptor 1 isoform 1	chr4:58435251-58553491	7.81505	23.5395	0.478863	0.014951
NM_177282	Mical2	protein-methionine sulfoxide oxidase MICAL2 isoform B	chr7:112225835-112355194	4.751	14.2812	0.477981	0.004567
NM_145823	Pitpnc1	cytoplasmic phosphatidylinositol transfer protein 1	chr11:107207891-107470720	1.34939	4.03232	0.47542	0.048088
NM_001166064	Syde2	rho GTPase-activating protein SYDE2	chr3:145987869-146021720	2.56272	7.65008	0.474965	0.034399
NM_008378	Impact	protein IMPACT	chr18:12972251-12992948	30.8027	91.8998	0.474724	0.002524
NM_172588	Serinc5	serine incorporator 5	chr13:92611137-92711946	4.62709	13.789	0.474225	0.004567
NM_013528	Gfpt1	glutamine--fructose-6-phosphate aminotransferase [isomerizing] 1	chr6:87042845-87092207	11.9784	35.6498	0.473659	0.002524
NM_133919	Aff1	AF4/FMR2 family member 1 isoform 2	chr5:103754161-103855322	5.70946	16.9475	0.472509	0.013662
NM_001081229	Tsc22d2	TSC22 domain family protein 2	chr3:58415688-58466787	3.63936	10.7555	0.470606	0.002524
NM_177152	Lrig3	leucine-rich repeats and immunoglobulin-like domains protein 3 precursor	chr10:125966218-126015359	3.4304	10.0849	0.468327	0.036128
NM_010729	Loxl1	lysyl oxidase homolog 1 precursor	chr9:58287722-58313212	47.7207	140.005	0.467436	0.002524

NM_001025438	Camk2d	calcium/calmodulin-dependent protein kinase type II subunit delta isoform 2	chr3:126596950-126846326	11.0602	32.4298	0.46718	0.002524
NM_172595	Arl15	ADP-ribosylation factor-like protein 15	chr13:113794507-114157461	3.36266	9.82214	0.465522	0.045466
NM_020590	Gabarapl1	gamma-aminobutyric acid receptor-associated protein-like 1	chr6:129532183-129542331	22.896	66.5095	0.463123	0.002524
NM_001145952	Lpp	lipoma-preferred partner homolog isoform 1	chr16:24392555-24992578	3.91156	11.3499	0.462644	0.048088
NR_030703	Snord104	N/A	chr11:106500990-106501063	55160.6	159626	0.461473	0.045466
NM_008973	Ptn	pleiotrophin precursor	chr6:36715662-36811361	18.2531	52.7763	0.461103	0.007984
NM_010380	H2-D1	H-2 class I histocompatibility antigen, D-B alpha chain precursor	chr17:35263093-35267497	39.1753	112.136	0.456735	0.002524
NM_207636	Fndc3a	fibronectin type-III domain-containing protein 3A	chr14:72537952-72710003	6.3154	18.0246	0.455464	0.002524
NM_029537	Tmem98	transmembrane protein 98	chr11:80810414-80822033	12.183	34.6351	0.453761	0.040429
NM_013727	Azi2	5-azacytidine-induced protein 2 isoform a	chr9:118040521-118150196	7.41244	21.0355	0.45299	0.016073
NM_019949	Ube2l6	ubiquitin/ISG15-conjugating enzyme E2 L6	chr2:84798827-84810003	11.5398	32.6956	0.452292	0.04259

NM_001284520	Cmah	cytidine monophosphate-N-acetylneuraminic acid hydroxylase isoform b	chr13:24327403-24477289	1.82291	5.15701	0.451632	0.006356
NM_001170537	Mef2c	myocyte-specific enhancer factor 2C isoform 1	chr13:83504033-83667079	2.02866	5.71796	0.450031	0.043282
NM_173747	Gpkow	G patch domain and KOW motifs-containing protein	chrX:7697133-7710259	6.17235	17.343	0.448673	0.002524
NM_133990	Il13ra1	interleukin-13 receptor subunit alpha-1 precursor	chrX:36112138-36171261	13.8459	38.8989	0.448616	0.002524
NM_001162999	Fnip2	folliculin-interacting protein 2	chr3:79345375-79567679	9.58444	26.8228	0.446936	0.002524
NM_019680	Elf4	ETS-related transcription factor Elf-4	chrX:48411048-48463132	3.59657	10.0503	0.446292	0.002524
NM_022993	Lrp10	low-density lipoprotein receptor-related protein 10 precursor	chr14:54464146-54470291	16.5602	46.2721	0.446256	0.002524
NM_007759	Crabp2	cellular retinoic acid-binding protein 2	chr3:87948692-87953372	14.9399	41.708	0.445871	0.041104
NM_001163567	Fam102b	protein FAM102B	chr3:108970996-109027607	20.3948	56.5625	0.443008	0.002524
NM_001039239	Zfp808	zinc finger protein 80	chr13:62129889-62173936	2.49059	6.90004	0.44255	0.033482
NM_026514	Cdc42ep3	cdc42 effector protein 3	chr17:79334024-79355091	11.0742	30.6449	0.442047	0.043961
NM_177798	Frs2	fibroblast growth factor receptor substrate 2	chr10:117070126-117148474	5.85857	16.1912	0.441488	0.002524
NM_027629	Pgm2l1	glucose 1,6-bisphosphate synthase	chr7:100227606-100278872	1.11149	3.06596	0.44066	0.049461
NM_138602	Praf2	PRA1 family protein 2	chrX:7728570-7731063	31.8732	87.9195	0.44066	0.002524

NM_009383	Tial1	nucleolysin TIAR	chr7:128439776-128461513	7.68818	21.1869	0.440244	0.002524
NM_028758	Gga2	ADP-ribosylation factor-binding protein GGA2	chr7:121986721-122021198	6.49222	17.828	0.438709	0.002524
NM_007513	Slc7a1	high affinity cationic amino acid transporter 1	chr5:148327409-148399904	6.23398	16.9826	0.435238	0.002524
NM_001002268	Adgrg6	G-protein coupled receptor 126 precursor	chr10:14402584-14545036	4.17945	11.3364	0.433357	0.007984
NM_001177780	Dlg3	disks large homolog 3 isoform 4	chrX:100767721-100818410	7.38399	20.0156	0.433077	0.013662
NM_178406	Gpr153	probable G-protein coupled receptor 153	chr4:152274361-152285337	7.89261	21.1945	0.429001	0.018718
NM_172525	Arhgap29	rho GTPase-activating protein 29	chr3:121953325-122016153	10.6721	28.5891	0.42795	0.002524
NM_175489	Osbpl8	oxysterol-binding protein-related protein 8 isoform a	chr10:111164801-111297247	4.91087	13.1491	0.42774	0.033482
NM_029492	Zdhhc20	probable palmitoyltransferase ZDHHC20	chr14:57832701-57890262	17.4693	46.6537	0.426611	0.002524
NM_008960	Pten	phosphatidylinositol 3,4,5-trisphosphate 3-phosphatase and dual-specificity protein phosphatase PTEN	chr19:32757576-32826160	29.6061	78.9541	0.425994	0.002524
NM_001017983	Foxred2	FAD-dependent oxidoreductase domain-containing protein 2 precursor	chr15:77940521-77956722	7.3483	19.5408	0.424756	0.004567
NM_009713	Arsa	arylsulfatase A precursor	chr15:89472475-89484850	6.10307	16.1872	0.423624	0.011083

NR_045517	Cldnd1	N/A	chr16:58727909-58734247	18.114	47.8763	0.422104	0.009547
NM_198095	Bst2	bone marrow stromal antigen 2 precursor	chr8:71534261-71537437	83.9939	221.488	0.421102	0.006356
NM_025972	Naaa	N-acylethanolamine-hydrolyzing acid amidase isoform 1 precursor	chr5:92257659-92278181	12.8707	33.6949	0.417959	0.012409
NM_170669	Rps15a	40S ribosomal protein S15a	chr7:118104375-118116147	30.0965	78.6802	0.417348	0.002524
NM_010276	Gem	GTP-binding protein GEM	chr4:11704446-11714993	11.935	31.1206	0.416225	0.034399
NM_008621	Mpp1	55 kDa erythrocyte membrane protein	chrX:75095853-75130949	26.3423	68.6182	0.415786	0.036128
NM_032000	Trps1	zinc finger transcription factor Trps1	chr15:50654758-50890041	7.65735	19.7633	0.411779	0.002524
NM_028975	Tmem33	transmembrane protein 33 isoform 1	chr5:67260564-67291461	8.33664	21.4735	0.410912	0.007984
NM_008549	Man2a1	alpha-mannosidase 2	chr17:64601648-64755110	13.0089	33.4179	0.409738	0.002524
NM_027519	Medag	mesenteric estrogen-dependent adipogenesis protein	chr5:149411805-149431703	8.62998	22.1439	0.409244	0.007984
NM_019913	Txn2	thioredoxin, mitochondrial precursor	chr15:77915050-77928994	40.0858	102.775	0.408898	0.002524
NM_172893	Parp12	poly [ADP-ribose] polymerase 12	chr6:39086411-39118349	14.8057	37.7424	0.4064	0.002524
NM_001009935	Txnip	thioredoxin-interacting protein isoform 1	chr3:96555767-96566801	16.4311	41.7359	0.404843	0.034399
NM_153459	Dusp7	dual specificity protein phosphatase 7	chr9:106368631-106375723	9.9406	25.1703	0.403477	0.021334

NM_172767	Vwa5a	von Willebrand factor A domain-containing protein 5A	chr9:38718267-38743337	7.10727	17.8878	0.400855	0.039029
NM_025301	Mrpl17	39S ribosomal protein L17, mitochondrial precursor	chr7:105803781-105811087	3.75579	9.43211	0.399909	0.016073
NM_022656	Nisch	nischarin	chr14:31170927-31206826	38.6499	96.8187	0.398811	0.002524
NM_175445	Rassf2	ras association domain-containing protein 2	chr2:131992849-132029988	6.31152	15.7874	0.398178	0.002524
NM_026684	Ndufb10	NADH dehydrogenase [ubiquinone] 1 beta subcomplex subunit 10	chr17:24722066-24724388	41.6807	104.254	0.398157	0.002524
NM_145959	D15Ertd621e	protein FAM91A1	chr15:58415467-58457801	33.4203	83.5504	0.397938	0.002524
NM_008332	Ifit2	interferon-induced protein with tetratricopeptide repeats 2	chr19:34550693-34576534	15.7444	39.3361	0.397667	0.002524
NM_022305	B4galt1	beta-1,4-galactosyltransferase 1	chr4:40804581-40854537	15.0152	37.5067	0.397579	0.002524
NM_001001566	Chpf	chondroitin sulfate synthase 2 isoform a	chr1:75474568-75479471	13.0953	32.6574	0.396869	0.036128
NM_027722	Nudt4	diphosphoinositol polyphosphate phosphohydrolase 2	chr10:95547006-95564167	48.0949	119.712	0.396041	0.002524
NM_001159900	Hopx	homeodomain-only protein	chr5:77086985-77115123	29.7467	73.4595	0.392609	0.030274
NM_013755	Gyg	glycogenin-1	chr3:20122083-20155116	11.7432	28.996	0.392555	0.022426
NM_029770	Unc5b	netrin receptor UNC5B precursor	chr10:60762594-60831581	12.9606	31.5402	0.38624	0.002524

NM_010685	Lamp2	lysosome-associated membrane glycoprotein 2 isoform 2 precursor	chrX:38401356-38456460	106.705	259.555	0.386044	0.02012
NM_028876	Tmed5	transmembrane emp24 domain-containing protein 5 precursor	chr5:108121646-108132591	14.9824	36.3566	0.385002	0.004567
NM_019946	Mgst1	microsomal glutathione S-transferase 1	chr6:138140536-138156752	77.6981	188.114	0.384009	0.004567
NM_022655	Ireb2	iron-responsive element-binding protein 2	chr9:54863754-54912534	7.59752	18.2584	0.380791	0.007984
NM_001286370	Ghr	growth hormone receptor isoform 1 precursor	chr15:3317754-3583352	12.2568	29.3672	0.379484	0.009547
NM_153586	Rbm41	RNA-binding protein 41 isoform 2	chrX:139889510-139998595	3.01369	7.22036	0.37946	0.028592
NM_007806	Cyba	cytochrome b-245 light chain isoform 1	chr8:122424770-122432940	42.8638	102.583	0.378985	0.0266
NM_030711	Erap1	endoplasmic reticulum aminopeptidase 1 precursor	chr13:74639871-74691875	8.43373	20.1684	0.378651	0.014951
NM_007616	Cav1	caveolin-1 isoform 1	chr6:17306334-17341328	100.735	238.967	0.375159	0.016073
NM_010299	Gm2a	ganglioside GM2 activator precursor	chr11:55097984-55113028	8.29956	19.636	0.374	0.013662
NM_010577	Itga5	integrin alpha-5 preproprotein	chr15:103344285-103366748	17.2125	40.6641	0.373364	0.004567
NM_011803	Klf6	Krueppel-like factor 6	chr13:5861488-5870393	19.4516	45.6584	0.370565	0.002524
NM_019571	Tspan5	tetraspanin-5	chr3:138742207-138904433	15.6896	36.5727	0.367546	0.045466
NM_011026	P2rx4	P2X purinoceptor 4	chr5:122707556-122729042	15.9929	37.2613	0.367329	0.018718

NM_144925	Tnrc6a	trinucleotide repeat-containing gene 6A protein	chr7:123123884-123195296	6.81592	15.8358	0.366116	0.002524
NM_001081241	Fam65a	protein FAM65A	chr8:105605228-105622218	4.56041	10.5693	0.365041	0.040429
NM_153525	Tmem41b	transmembrane protein 41B	chr7:109972186-109986230	14.0695	32.5329	0.364045	0.018718
NM_009551	Zfand5	AN1-type zinc finger protein 5	chr19:21272277-21286840	10.8783	25.1013	0.363135	0.006356
NM_134012	Mbtd1	MBT domain-containing protein 1	chr11:93886218-93946984	4.53558	10.4634	0.363042	0.029348
NM_007798	Ctsb	cathepsin B preproprotein	chr14:63122461-63179578	94.4964	217.986	0.363015	0.014951
NM_145360	Idi1	isopentenyl-diphosphate Delta-isomerase 1	chr13:8885605-8892396	27.568	63.2768	0.360839	0.002524
NM_178060	Thra	thyroid hormone receptor alpha	chr11:98741872-98765111	15.5356	35.5457	0.359457	0.004567
NM_001111044	Serpinh1	serpin H1 precursor	chr7:99345374-99353239	168.619	384.835	0.358367	0.011083
NM_175121	Slc38a2	sodium-coupled neutral amino acid transporter 2	chr15:96687391-96699698	83.4112	189.902	0.357305	0.009547
NM_177586	Eif5a2	eukaryotic translation initiation factor 5A-2	chr3:28781310-28798846	5.55137	12.616	0.356525	0.030873
NM_008321	Id3	DNA-binding protein inhibitor ID-3	chr4:136143821-136145392	92.1023	208.327	0.354475	0.004567
NM_001097644	Ccnyl1	cyclin-Y-like protein 1	chr1:64691344-64725642	12.2833	27.6209	0.351925	0.018718
NM_007901	S1pr1	sphingosine 1-phosphate receptor 1	chr3:115710432-115715055	14.0159	31.4628	0.351176	0.012409
NM_001029982	Sec23ip	SEC23-interacting protein	chr7:128744869-128784835	9.82258	22.0269	0.350727	0.007984
NM_016900	Cav2	caveolin-2 isoform 1	chr6:17281184-17289130	42.7794	95.6627	0.349508	0.006356

NM_011083	Pik3c2a	phosphatidylinositol 4-phosphate 3-kinase C2 domain-containing subunit alpha	chr7:116337275-116443458	12.1444	27.1052	0.348674	0.006356
NM_001177871	Filip1l	filamin A-interacting protein 1-like isoform 2	chr16:57301999-57606867	19.5699	43.5833	0.347732	0.007984
NR_003517	Pisd-ps1	N/A	chr11:3124020-3193463	30.2035	67.1599	0.347054	0.004567
NM_019835	B4galt5	beta-1,4-galactosyltransferase 5	chr2:167298444-167349178	25.1589	55.8592	0.346401	0.011083
NM_007554	Bmp4	bone morphogenetic protein 4 precursor	chr14:46379523-46390599	21.6802	48.0913	0.346004	0.014951
NM_025593	Polr2l	DNA-directed RNA polymerases I, II, and III subunit RPABC5	chr7:141471859-141475153	18.324	40.5715	0.3452	0.043282
NM_008788	Pcolce	procollagen C-endopeptidase enhancer 1 precursor	chr5:137605106-137611404	217.281	480.884	0.345016	0.007984
NM_001033276	Kmt2d	histone-lysine N-methyltransferase 2D	chr15:98831668-98871205	2.22276	4.89498	0.342858	0.021334
NM_183020	Atxn2l	ataxin-2-like protein	chr7:126491707-126503302	6.32214	13.9225	0.342852	0.041104
NM_010270	Mrps33	28S ribosomal protein S33, mitochondrial isoform 1	chr6:39801806-39810936	33.0398	72.2988	0.340095	0.025561
NM_008871	Serpine1	plasminogen activator inhibitor 1 precursor	chr5:137061505-137072272	38.6044	84.2368	0.338866	0.0266
NM_009951	Igf2bp1	insulin-like growth factor 2 mRNA-binding protein 1	chr11:95957163-96005944	7.12938	15.533	0.338204	0.012409

NM_001033380	Itpripl2	inositol 1,4,5-trisphosphate receptor-interacting protein-like 2 precursor	chr7:118485111-118491975	13.9526	30.3705	0.337795	0.013662
NM_177151	Vps13b	vacuolar protein sorting-associated protein 13B	chr15:35371545-35931229	6.71226	14.5978	0.337419	0.007984
NM_001081267	Rsf1	remodeling and spacing factor 1	chr7:97579895-97692782	4.20345	9.14052	0.337364	0.006356
NM_025813	Mfsd1	major facilitator superfamily domain-containing protein 1	chr3:67582767-67604231	69.4032	150.831	0.337111	0.006356
NM_175174	Klhl5	kelch-like protein 5	chr5:65131230-65168142	12.7408	27.6618	0.336684	0.027658
NM_013825	Ly75	lymphocyte antigen 75 precursor	chr2:60293759-60383231	18.2997	39.71	0.336455	0.016073
NM_010763	Man1a2	mannosyl-oligosaccharide 1,2-alpha-mannosidase IB	chr3:100562204-100685473	17.2263	37.337	0.335946	0.007984
NM_144886	Exosc2	exosome complex component RRP4	chr2:31670736-31681307	13.5505	29.2883	0.334739	0.024582
NM_146019	Chd3	chromodomain-helicase-DNA-binding protein 3	chr11:69343272-69369426	9.87847	21.2669	0.333014	0.018718
NM_178764	Fam168a	protein FAM168A	chr7:100706701-100841630	5.47319	11.7304	0.331073	0.025561
NM_001302957	Ttll7	tubulin polyglutamylase TTLL7 isoform Ttll7	chr3:146852366-146984008	7.27272	15.5824	0.330937	0.018718
NM_175266	Epm2aip1	EPM2A-interacting protein 1	chr9:111271844-111279091	6.45499	13.8167	0.330507	0.030873
NM_026667	Fam114a1	protein Noxp20	chr5:64970074-65041901	15.7014	33.5388	0.32961	0.024582
NM_146234	Mmgt1	membrane magnesium transporter 1 precursor	chrX:56585511-56597919	16.3163	34.8515	0.329598	0.011083
NM_175675	Slc35f6	solute carrier family 35 member F6 precursor	chr5:30647935-30659729	16.5389	35.3107	0.329399	0.02012

NM_013790	Abcc5	multidrug resistance-associated protein 5 isoform 1	chr16:20331303-20426394	9.6871	20.6816	0.32939	0.028592
NM_023409	Npc2	epididymal secretory protein E1 precursor	chr12:84754558-84773112	63.9037	136.389	0.329255	0.013662
NM_001085492	Rere	arginine-glutamic acid dipeptide repeats protein	chr4:150281915-150621966	4.48501	9.55833	0.328619	0.035191
NM_030250	Nus1	dehydrodolichyl diphosphate syntase complex subunit Nus1	chr10:52417546-52440192	12.3091	26.1123	0.326618	0.030873
NM_025863	Trim59	tripartite motif-containing protein 59	chr3:69035293-69044742	18.4375	38.9554	0.324866	0.014951
NM_008037	Fosl2	fos-related antigen 2	chr5:32136471-32157839	25.1863	53.1667	0.324474	0.016073
NM_178606	Reep3	receptor expression-enhancing protein 3 isoform 2	chr10:67005074-67096988	20.077	42.3623	0.324282	0.006356
NM_029730	Mospd2	motile sperm domain-containing protein 2 isoform 1	chrX:164936170-164980375	9.81019	20.6347	0.322921	0.046295
NM_148952	E2f4	transcription factor E2F4	chr8:105297662-105305370	10.5651	22.1638	0.321771	0.040429
NM_001042752	Neo1	neogenin isoform 2 precursor	chr9:58874679-59036441	17.8174	37.3697	0.321678	0.013662
NM_001081043	Ptpn23	tyrosine-protein phosphatase non-receptor type 23	chr9:110385088-110408210	5.60641	11.7024	0.319592	0.047481
NM_010436	H2afx	histone H2AX	chr9:44334714-44336073	92.2782	191.509	0.31709	0.030274
NM_028298	Zfp655	zinc finger protein 655 isoform a	chr5:145231714-145247306	10.2732	21.1954	0.314534	0.027658

NM_016783	Pgrmc1	membrane-associated progesterone receptor component 1	chrX:36598224-36606079	76.1868	155.741	0.310524	0.016073
NM_029250	Etnk1	ethanolamine kinase 1	chr6:143167229-143208547	13.7984	28.0095	0.307475	0.034399
NM_022995	Pmepa1	protein TMEPAI	chr2:173224464-173276533	14.6079	29.6168	0.306951	0.023435
NM_009179	St3gal2	CMP-N-acetylneuraminate-beta-galactosamide- alpha-2,3-sialyltransferase 2	chr8:110919864-110972497	20.9776	42.3461	0.305058	0.016073
NM_011101	Prkca	protein kinase C alpha type	chr11:107933386-108343888	7.00614	14.1315	0.304712	0.028592
NM_213659	Stat3	signal transducer and activator of transcription 3 isoform 1	chr11:100886809-100939511	20.0011	40.1637	0.302779	0.04259
NM_145619	Parp3	poly [ADP-ribose] polymerase 3	chr9:106470352-106476651	29.2913	58.7636	0.30237	0.022426
NM_181070	Rab18	ras-related protein Rab-18 isoform 2 precursor	chr18:6765166-6791606	35.5411	71.1606	0.301509	0.017468
NM_001033273	5031439G07	uncharacterized protein KIAA0930 homolog	chr15:84945719-84987971	14.3873	28.5379	0.297441	0.028592
NM_173443	Vcpip1	deubiquitinating protein VCIP135	chr1:9718621-9771256	6.53049	12.9294	0.296634	0.035191
NM_026582	Wls	protein wntless homolog precursor	chr3:159839694-159935175	109.331	215.956	0.295621	0.039029
NM_028266	Col16a1	collagen alpha-1(XVI) chain precursor	chr4:130047839-130099277	12.7746	25.215	0.29531	0.030873
NM_023598	Arid5b	AT-rich interactive domain-containing protein 5B	chr10:68095592-68278726	11.2096	22.1122	0.295043	0.035191

NM_011952	Mapk3	mitogen-activated protein kinase 3	chr7:126759625-126765816	36.7793	72.3862	0.294052	0.045466
NM_201369	N4bp2l2	NEDD4-binding protein 2-like 2	chr5:150635972-150665612	10.5794	20.8043	0.293692	0.040429
NM_172712	Uba6	ubiquitin-like modifier-activating enzyme 6	chr5:86110729-86172743	12.1798	23.9448	0.293571	0.046989
NM_011163	Eif2ak2	interferon-induced, double-stranded RNA-activated protein kinase	chr17:78852549-78882572	15.5305	30.4896	0.292968	0.049461
NM_013813	Epb4.1l3	band 4.1-like protein 3	chr17:69156809-69289987	36.1506	70.9322	0.292728	0.030873
NM_011594	Timp2	metalloproteinase inhibitor 2 precursor	chr11:118301060-118355411	124.114	243.429	0.292552	0.033482
NM_013654	Ccl7	C-C motif chemokine 7 precursor	chr11:82045711-82047523	119.403	234.024	0.292246	0.044936
NM_172689	Ddx58	probable ATP-dependent RNA helicase DDX58	chr4:40203776-40239825	12.7383	24.9122	0.2913	0.035191
NM_020606	Parva	alpha-parvin	chr7:112427705-112591688	20.4373	39.4618	0.285753	0.03705
NM_009994	Cyp1b1	cytochrome P450 1B1	chr17:79706952-79715041	23.4901	44.9449	0.281794	0.045466
NM_146145	Jak1	tyrosine-protein kinase JAK1	chr4:101069037-101265282	32.8911	62.9146	0.281673	0.042004
NM_009320	Slc6a6	sodium- and chloride-dependent taurine transporter	chr6:91684066-91759063	31.39	59.8568	0.280322	0.032599
NM_001031814	Smg1	serine/threonine-protein kinase SMG1	chr7:118131311-118243637	9.1604	17.4571	0.280057	0.048747
NM_025283	Mob4	MOB-like protein phocein	chr1:55131244-55154899	24.0467	45.6642	0.278521	0.049461
NM_001142655	Arpp19	cAMP-regulated phosphoprotein 19 isoform 2	chr9:75037613-75060313	34.1039	64.0769	0.273897	0.045466

NM_001024952	Rc3h1	roquin-1	chr1:160906410-160974976	7.89806	14.7898	0.272442	0.048747
NM_028288	Cul4b	cullin-4B	chrX:38531620-38576196	29.1334	53.8488	0.266785	0.045466
NM_009127	Scd1	acyl-CoA desaturase 1	chr19:44394449-44407709	73.3939	39.798	-0.2658	0.049461
NM_025587	Rps21	40S ribosomal protein S21	chr2:180257378-180258444	2503.26	1353.61	-0.26701	0.043282
NM_011546	Zeb1	zinc finger E-box-binding homeobox 1	chr18:5491500-5775468	35.1824	18.8685	-0.27059	0.046989
NM_001293622	Ppp3ca	serine/threonine-protein phosphatase 2B catalytic subunit alpha isoform isoform 2	chr3:136670065-136937727	73.8933	39.552	-0.27144	0.046989
NM_008947	Psmc1	26S protease regulatory subunit 4	chr12:100112330-100123364	116.614	62.0619	-0.27393	0.047481
NM_028233	Lrpprc	leucine-rich PPR motif-containing protein, mitochondrial precursor	chr17:84705246-84790786	64.6482	34.3296	-0.27489	0.038016
NM_010925	Rrp1	ribosomal RNA processing protein 1 homolog A	chr10:78400361-78413043	140.452	74.3809	-0.27607	0.038016
NM_026147	Rps20	40S ribosomal protein S20	chr4:3834472-3835600	2701.23	1428.47	-0.27669	0.04259
NM_025974	Rpl14	60S ribosomal protein L14	chr9:120571516-120574653	602.599	317.42	-0.27839	0.040429
NM_010145	Ephx1	epoxide hydrolase 1 precursor	chr1:180989555-181017495	88.4272	46.344	-0.28059	0.048747
NM_009366	Tsc22d1	TSC22 domain family protein 1 isoform 2	chr14:76415820-76507766	165.057	86.4738	-0.28075	0.043961
NM_011874	Psmc4	26S protease regulatory subunit 6B	chr7:28041701-28050092	164.13	85.735	-0.28203	0.048088
NM_028782	Lonp1	lon protease homolog, mitochondrial precursor	chr17:56614297-56626903	123.903	64.7076	-0.28213	0.031798

NM_007951	Erh	enhancer of rudimentary homolog	chr12:80634022-80643861	346.235	180.64	-0.28256	0.048088
NM_026319	Ift74	intraflagellar transport protein 74 homolog isoform 1	chr4:94614490-94693233	44.3362	23.0719	-0.28368	0.048747
NM_026027	Pfdn1	prefoldin subunit 1	chr18:36403678-36454495	174.374	90.33	-0.28565	0.04259
NM_024175	Rps23	40S ribosomal protein S23	chr13:90923121-90924732	1375.31	712.234	-0.28578	0.048747
NM_133666	Ndufv1	NADH dehydrogenase [ubiquinone] flavoprotein 1, mitochondrial precursor	chr19:4007498-4012755	154.867	79.9062	-0.28738	0.033482
NM_011739	Ywhaq	14-3-3 protein theta	chr12:21390328-21417436	233.949	119.594	-0.29141	0.030873
NM_024176	Drap1	dr1-associated corepressor isoform 2	chr19:5406814-5424979	168.603	85.6536	-0.29412	0.039854
NM_001081242	Tln2	talin-2	chr9:67217084-67559703	9.02442	4.57214	-0.2953	0.047481
NM_028044	Cnn3	calponin-3	chr3:121426540-121458205	440.141	222.201	-0.29685	0.039854
NM_025338	Aurkaip1	aurora kinase A-interacting protein	chr4:155831268-155833098	132.481	66.8685	-0.29693	0.043961
NM_172406	Trak2	trafficking kinesin-binding protein 2	chr1:58900449-58973482	27.2684	13.7301	-0.29799	0.031798
NM_009077	Rpl18	60S ribosomal protein L18	chr7:45718070-45720835	1361.05	684.537	-0.29848	0.027658
NM_023133	Rps19	40S ribosomal protein S19	chr7:24884713-24889802	841.716	421.368	-0.3005	0.033482
NM_175666	Hist2h2bb	histone H2B type 2-B	chr3:96269699-96270192	3344.36	1669.26	-0.30179	0.02012
NM_026943	Snrpd2	small nuclear ribonucleoprotein Sm D2	chr7:19149837-19152726	448.155	223.489	-0.30217	0.031798
NM_026885	Chmp2a	charged multivesicular body protein 2a	chr7:13032005-13034777	135.749	67.6849	-0.30225	0.03705

NM_008948	Psmc3	26S protease regulatory subunit 6A	chr2:91054015-91059438	284.225	141.567	-0.3027	0.022426
NM_010948	Nudc	nuclear migration protein nudC	chr4:133532541-133546027	172.848	86.0408	-0.30296	0.030873
NM_146239	Cdk17	cyclin-dependent kinase 17	chr10:93160875-93241342	34.9655	17.4018	-0.30304	0.025561
NM_009510	Ezr	ezrin	chr17:6738130-6782780	68.2779	33.9623	-0.30328	0.0266
NM_025548	Tbcb	tubulin-folding cofactor B	chr7:30224130-30232029	83.4936	41.4534	-0.30409	0.047481
NM_008634	Map1b	microtubule-associated protein 1B	chr13:99421463-99516602	15.7659	7.82165	-0.30442	0.031798
NM_153065	Ddx27	probable ATP-dependent RNA helicase DDX27	chr2:167015312-167034945	59.6261	29.5607	-0.30472	0.032599
NM_001013777	Zfp488	zinc finger protein 488	chr14:33967069-33978764	54.3825	26.9551	-0.30482	0.028592
NM_019816	Aatf	protein AATF	chr11:84422855-84513501	35.9588	17.7463	-0.3067	0.04259
NM_028283	Uaca	uveal autoantigen with coiled-coil domains and ankyrin repeats	chr9:60794547-60880370	16.3925	8.06672	-0.30795	0.04259
NM_009081	Rpl28	60S ribosomal protein L28	chr7:4792964-4794547	1541.65	756.272	-0.30931	0.023435
NM_054102	Ivns1abp	influenza virus NS1A-binding protein homolog isoform 2	chr1:151344497-151364445	78.3444	38.4232	-0.30941	0.030274
NM_011239	Ranbp1	ran-specific GTPase-activating protein	chr16:18239978-18248694	465.165	226.89	-0.31179	0.022426
NM_016696	Gpc1	glypican-1 precursor	chr1:92831685-92860196	152.623	74.3543	-0.31231	0.0266
NM_178598	Tagln2	transgelin-2	chr1:172500245-172507375	137.42	66.8753	-0.31279	0.029348
NM_138745	Mthfd1	C-1-tetrahydrofolate synthase, cytoplasmic	chr12:76255231-76319820	52.7933	25.63	-0.31383	0.027658

NM_016774	Atp5b	ATP synthase subunit beta, mitochondrial precursor	chr10:128083306-128090388	1251.32	606.086	-0.31484	0.030873
NM_177470	Acaa2	3-ketoacyl-CoA thiolase, mitochondrial	chr18:74779211-74806207	84.8798	41.1057	-0.3149	0.035191
NM_153098	Cd109	CD109 antigen precursor	chr9:78615545-78716260	56.7726	27.4779	-0.31515	0.012409
NM_012021	Prdx5	peroxiredoxin-5, mitochondrial precursor	chr19:6906818-6909645	449.237	217.008	-0.316	0.030873
NM_016846	Rgl1	ral guanine nucleotide dissociation stimulator-like 1	chr1:152517529-152625111	31.1393	14.9819	-0.31774	0.030873
NM_207237	Man1c1	mannosyl-oligosaccharide 1,2-alpha-mannosidase IC	chr4:134561689-134704290	17.7544	8.53731	-0.31799	0.043282
NR_033535	Gm10845	N/A	chr14:79860520-79869176	42.6285	20.4318	-0.31939	0.022426
NM_024413	Plekhf1	pleckstrin homology domain-containing family F member 1	chr7:38220653-38227994	70.6436	33.8079	-0.32006	0.032599
NM_172903	Man2a2	alpha-mannosidase 2x	chr7:80349096-80371375	30.2005	14.4465	-0.32025	0.017468
NM_007898	Ebp	3-beta-hydroxysteroid-Delta(8), Delta(7)-isomerase	chrX:8185330-8193512	93.4843	44.7115	-0.32032	0.0266
NM_025441	Nemf	nuclear export mediator factor Nemf	chr12:69311542-69357176	31.8754	15.1994	-0.32163	0.023435
NM_013470	Anxa3	annexin A3	chr5:96793384-96845968	332.929	158.141	-0.32331	0.022426
NM_153153	Svil	supervillin	chr18:5046588-5119293	11.363	5.38768	-0.32409	0.030274
NM_026483	Mphosph10	U3 small nucleolar ribonucleoprotein protein MPP10	chr7:64376540-64392236	22.2353	10.4167	-0.32931	0.035191
NM_178215	Hist2h3b	histone H3.2	chr3:96268653-96269155	446.13	208.998	-0.32932	0.046989

NM_025840	Bzw2	basic leucine zipper and W2 domain-containing protein 2	chr12:36091834-36156825	52.9358	24.7882	-0.3295	0.0266
NM_145475	Cerk	ceramide kinase	chr15:86139100-86186141	27.1956	12.7312	-0.32963	0.028592
NM_030113	Arhgap10	rho GTPase-activating protein 10	chr8:77250365-77518578	35.5449	16.6222	-0.33009	0.016073
NM_019746	Pdcd5	programmed cell death protein 5	chr7:35641984-35647482	232.658	108.579	-0.33097	0.025561
NM_197982	Ddx39	ATP-dependent RNA helicase DDX39A	chr8:83715176-83741311	139.672	65.1082	-0.33147	0.048088
NM_001305286	Mpdz	multiple PDZ domain protein isoform 3	chr4:81278498-81442815	32.0145	14.8475	-0.33369	0.016073
NM_011412	Slit3	slit homolog 3 protein precursor	chr11:35121455-35708507	15.4277	7.12304	-0.33564	0.04259
NM_008292	Hsd17b4	peroxisomal multifunctional enzyme type 2	chr18:50128200-50196270	74.1655	34.199	-0.33619	0.014951
NM_027130	Afg3l2	AFG3-like protein 2	chr18:67404763-67449136	40.0155	18.3232	-0.33923	0.014951
NM_024197	Ndufa10	NADH dehydrogenase [ubiquinone] 1 alpha subcomplex subunit 10, mitochondrial precursor	chr1:92439718-92473758	120.691	55.238	-0.33944	0.009547
NM_008398	Itga7	integrin alpha-7	chr10:128933812-128960988	17.7998	8.14303	-0.33963	0.043961
NM_027706	Nbas	neuroblastoma-amplified sequence	chr12:13269126-13583811	10.6692	4.8801	-0.3397	0.033482
NM_011794	Bpnt1	3'(2'),5'-bisphosphate nucleotidase 1	chr1:185332158-185357769	35.7028	16.2591	-0.34161	0.039029
NM_020569	Park7	protein DJ-1	chr4:150897132-150909921	251.866	114.526	-0.34226	0.009547
NM_001080743	Grk4	G protein-coupled receptor kinase 4 isoform 2	chr5:34660378-34755303	23.6981	10.7704	-0.34248	0.014951

NM_175318	Spty2d1	protein SPT2 homolog	chr7:46990395-47008414	12.9152	5.86981	-0.34248	0.032599
NM_176849	Arglu1	arginine and glutamate-rich protein 1	chr8:8666575-8690537	83.8896	37.919	-0.34485	0.013662
NM_007739	Col8a1	collagen alpha-1(VIII) chain precursor	chr16:57624255-57754737	103.044	46.4523	-0.34601	0.009547
NM_008136	Gnl1	guanine nucleotide-binding protein-like 1	chr17:35979954-35989462	52.6258	23.7096	-0.34627	0.013662
NR_033146	Gm17821	N/A	chr12:67656741-67669271	19.1515	8.62101	-0.34665	0.011083
NM_026964	Ccdc124	coiled-coil domain-containing protein 124	chr8:70868226-70873490	118.816	53.4772	-0.34671	0.02012
NM_023284	Nuf2	kinetochore protein Nuf2	chr1:169497933-169531464	52.1419	23.4085	-0.34781	0.012409
NM_011304	Ruvbl2	ruvB-like 2	chr7:45421897-45434464	98.3752	43.9418	-0.35001	0.012409
NM_134469	Fdps	farnesyl pyrophosphate synthase isoform 2	chr3:89093587-89101967	189.128	84.4336	-0.35024	0.009547
NM_008343	Igfbp3	insulin-like growth factor-binding protein 3 precursor	chr11:7206090-7213923	27.5389	12.2528	-0.35171	0.029348
NM_179203	Atad3a	ATPase family AAA domain-containing protein 3	chr4:155740639-155761098	51.805	23.0077	-0.3525	0.013662
NM_133900	Psph	phosphoserine phosphatase	chr5:129765557-129787253	51.2419	22.5816	-0.35587	0.036128
NM_172920	Dpy19l1	probable C-mannosyltransferase DPY19L1	chr9:24411778-24503140	93.1472	40.9445	-0.35697	0.004567
NM_001293559	Cox4i1	cytochrome c oxidase subunit 4 isoform 1, mitochondrial isoform 2	chr8:120668224-120674209	1146.1	500.754	-0.3596	0.025561
NM_020600	Rps14	40S ribosomal protein S14	chr18:60774595-60778546	1542.56	670.011	-0.36216	0.002524

NM_030609	Hist1h1a	histone H1.1	chr13:23763667-23764412	949.543	410.411	-0.3643	0.006356
NM_008910	Ppm1a	protein phosphatase 1A	chr12:72761210-72794940	48.8395	20.9714	-0.36715	0.009547
NM_010324	Got1	aspartate aminotransferase, cytoplasmic	chr19:43499752-43524605	31.6778	13.4018	-0.37359	0.036128
NM_027117	Klhdc2	kelch domain-containing protein 2	chr12:69296680-69310687	70.4456	29.7071	-0.37499	0.002524
NM_021436	Tmeff1	tomoregulin-1 precursor	chr4:48585192-48663131	31.0473	13.0226	-0.37733	0.030873
NM_001081300	Tshz1	teashirt homolog 1	chr18:84011626-84086562	16.4808	6.90552	-0.37778	0.006356
NM_023374	Sdhb	succinate dehydrogenase [ubiquinone] iron-sulfur subunit, mitochondrial precursor	chr4:140961270-140979192	192.449	80.5703	-0.37814	0.007984
NM_010879	Nck2	cytoplasmic protein NCK2	chr1:43445750-43570518	28.4162	11.852	-0.37978	0.011083
NM_010295	Gclc	glutamate--cysteine ligase catalytic subunit	chr9:77754534-77794489	27.2642	11.3119	-0.38206	0.007984
NM_153507	Cpne2	copine-2	chr8:94533027-94570529	44.4416	18.4176	-0.38255	0.006356
NM_172575	Zfp277	zinc finger protein 277 isoform 1	chr12:40315045-40445790	50.5149	20.9125	-0.38301	0.004567
NM_007631	Ccnd1	G1/S-specific cyclin-D1	chr7:144929930-144939925	77.5989	32.0996	-0.38336	0.002524
NM_024434	Lap3	cytosol aminopeptidase	chr5:45493373-45512691	136.603	56.4009	-0.38417	0.002524
NM_021366	Klf13	Krueppel-like factor 13	chr7:63886350-63938915	13.1237	5.37338	-0.38781	0.014951
NM_026960	Gsdmd	gasdermin-D	chr15:75862338-75867404	30.4536	12.4542	-0.38832	0.043282
NM_026232	Slc25a30	kidney mitochondrial carrier protein 1	chr14:75761998-75787037	30.0747	12.2668	-0.38947	0.047481
NM_001290308	Col12a1	collagen alpha-1(XII) chain precursor	chr9:79598986-79718722	84.622	34.4287	-0.39056	0.002524

NM_026821	Lurap1l	leucine rich adaptor protein 1-like	chr4:80910685-80954301	59.7867	24.2732	-0.39148	0.016073
NM_013614	Odc1	ornithine decarboxylase	chr12:17544872-17551502	157.321	62.9245	-0.39797	0.002524
NM_008303	Hspe1	10 kDa heat shock protein, mitochondrial	chr1:55088147-55091317	561.684	224.35	-0.39857	0.002524
NM_007408	Plin2	perilipin-2	chr4:86656564-86670059	174.145	69.1695	-0.401	0.002524
NM_033524	Spred1	sprouty-related, EVH1 domain-containing protein 1 isoform 1	chr2:117121070-117182277	51.2524	20.3358	-0.40145	0.002524
NM_134065	Epdr1	mammalian ependymin-related protein 1 precursor	chr13:19591707-19619830	37.9239	14.9876	-0.40318	0.021334
NM_010730	Anxa1	annexin A1	chr19:20373433-20390671	743.801	293.905	-0.40325	0.002524
NM_053162	Mrpl34	39S ribosomal protein L34, mitochondrial	chr8:71464925-71465753	130.749	51.6484	-0.40338	0.040429
NR_045297	Gm17644	N/A	chr1:12667562-12673090	45.6848	18.0188	-0.40405	0.013662
NM_178679	Zfp365	protein ZNF365	chr10:67886104-67912662	11.6114	4.55672	-0.40623	0.047481
NM_025551	Ndufa12	NADH dehydrogenase [ubiquinone] 1 alpha subcomplex subunit 12	chr10:94199008-94220948	464.236	181.309	-0.40832	0.002524
NM_001135559	Sos2	son of sevenless homolog 2	chr12:69583760-69681852	8.79834	3.3882	-0.41443	0.045466
NM_145456	Zswim6	zinc finger SWIM domain-containing protein 6	chr13:107724616-107890064	9.72457	3.71238	-0.41822	0.032599
NM_025831	Pxdc1	PX domain-containing protein 1	chr13:34627840-34652681	28.254	10.7739	-0.41871	0.047481
NM_011581	Thbs2	thrombospondin-2 precursor	chr17:14665499-14694262	81.8212	31.0476	-0.42084	0.002524
NM_028419	Glrx5	glutaredoxin-related protein 5, mitochondrial	chr12:105032688-105040910	78.5186	29.5813	-0.42396	0.043961

NM_001033445	Garem	GRB2-associated and regulator of MAPK protein	chr18:21127341-21300139	8.86628	3.33723	-0.42436	0.043282
NM_030565	Fam20c	extracellular serine/threonine protein kinase FAM20C precursor	chr5:138755080-138810063	14.0896	5.28402	-0.42593	0.041104
NM_013602	Mt1	metallothionein-1	chr8:94179088-94180327	2207.34	823.131	-0.4284	0.002524
NM_013454	Abca1	ATP-binding cassette sub-family A member 1	chr4:53030788-53159895	31.1438	11.4423	-0.43486	0.002524
NM_009929	Col18a1	collagen alpha-1(XVIII) chain isoform 2 precursor	chr10:77052178-77166530	21.1599	7.74468	-0.43651	0.002524
NM_025968	Ptgr1	prostaglandin reductase 1	chr4:58965589-58987078	24.0159	8.76481	-0.43775	0.032599
NM_016701	Nes	nestin	chr3:87971092-87980451	43.2816	15.7486	-0.43906	0.002524
NM_009728	Atp10a	probable phospholipid-transporting ATPase VA	chr7:58658201-58829426	20.4815	7.41758	-0.4411	0.002524
NM_144880	Ppp2r5a	serine/threonine-protein phosphatase 2A 56 kDa regulatory subunit alpha isoform	chr1:191325964-191397041	82.4397	29.5843	-0.44508	0.002524
NM_013834	Sfrp1	secreted frizzled-related protein 1 precursor	chr8:23411501-23449632	17.1488	6.14851	-0.44546	0.023435
NM_011693	Vcam1	vascular cell adhesion protein 1 precursor	chr3:116110019-116129688	48.4851	17.3638	-0.44596	0.002524
NM_011633	Traf5	TNF receptor-associated factor 5	chr1:191997202-192092599	15.0704	5.39397	-0.44622	0.044936
NM_181348	Prune2	protein prune homolog 2	chr19:16956117-17223932	6.01052	2.14994	-0.44648	0.002524
NM_021278	Tmsb4x	thymosin beta-4	chrX:167207093-167209218	1068.08	379.493	-0.4494	0.002524

NM_013626	Pam	peptidyl-glycine alpha-amidating monooxygenase precursor	chr1:97821093-98095632	93.9809	33.2383	-0.4514	0.002524
NM_027491	Rragd	ras-related GTP-binding protein D	chr4:32982997-33022180	10.435	3.68173	-0.45244	0.027658
NM_010415	Hbegf	proheparin-binding EGF-like growth factor precursor	chr18:36504926-36515805	73.3986	25.8423	-0.45335	0.002524
NM_021788	Sap30	histone deacetylase complex subunit SAP30	chr8:57482701-57487860	51.4449	18.0826	-0.45408	0.007984
NM_001085500	Cisd3	CDGSH iron-sulfur domain-containing protein 3, mitochondrial precursor	chr11:97685951-97688625	60.9102	20.9517	-0.46347	0.047481
NM_015787	Hist1h1e	histone H1.4	chr13:23621776-23622558	971.559	330.113	-0.46881	0.002524
NM_016919	Col5a3	collagen alpha-3(V) chain precursor	chr9:20770049-20815034	6.03387	2.03344	-0.47236	0.016073
NM_007664	Cdh2	cadherin-2 precursor	chr18:16588876-16809049	98.1023	32.9236	-0.47417	0.002524
NM_001048058	Rpl38	60S ribosomal protein L38	chr11:114344525-114757886	4720.73	1572	-0.47756	0.024582
NM_007414	Adprh	ADP-ribosylarginine hydrolase	chr16:38445398-38452689	56.4805	18.7781	-0.47825	0.006356
NM_172668	Lrp4	low-density lipoprotein receptor-related protein 4 isoform 1 precursor	chr2:91457530-91513901	4.64982	1.54238	-0.47925	0.042004
NM_001277149	Chd7	chromodomain-helicase-DNA-binding protein 7	chr4:8690405-8868449	2.73939	0.900243	-0.48329	0.03705

NM_027455	Qpct	glutaminyl-peptide cyclotransferase precursor	chr17:79051905-79090243	25.0053	8.19901	-0.48427	0.029348
NM_008969	Ptgs1	prostaglandin G/H synthase 1 precursor	chr2:36230425-36252271	136.434	44.634	-0.48526	0.002524
NM_019641	Stmn1	stathmin	chr4:134468319-134473843	462.791	151.071	-0.48621	0.002524
NM_011382	Six4	homeobox protein SIX4	chr12:73100258-73113245	10.2616	3.32386	-0.48957	0.030873
NM_009932	Col4a2	collagen alpha-2(IV) chain preproprotein	chr8:11312828-11449287	67.7551	21.8973	-0.49055	0.002524
NM_008982	Ptprj	receptor-type tyrosine-protein phosphatase eta isoform 1	chr2:90429755-90580647	10.9373	3.51094	-0.49348	0.007984
NM_013725	Rps11	40S ribosomal protein S11	chr7:45122387-45124389	968.486	308.098	-0.4974	0.002524
NM_024236	Qdpr	dihydropteridine reductase	chr5:45434031-45450229	92.6858	29.4237	-0.49832	0.002524
NM_007494	Ass1	argininosuccinate synthase	chr2:31470269-31520670	164.628	52.2529	-0.49839	0.002524
NR_045764	4930470H14	N/A	chr17:4044657-4082995	153.486	48.5833	-0.49958	0.002524
NM_007836	Gadd45a	growth arrest and DNA damage-inducible protein GADD45 alpha	chr6:67035095-67080652	58.0646	18.1328	-0.50545	0.009547
NM_030749	Sil1	nucleotide exchange factor SIL1 precursor	chr18:35266395-35498925	42.5513	13.1767	-0.50911	0.002524
NM_001040611	Peg10	retrotransposon-derived protein PEG10 isoform 2	chr6:4747305-4760516	72.9238	22.4083	-0.51246	0.002524
NM_008926	Prkg2	cGMP-dependent protein kinase 2	chr5:98929772-99037079	35.7584	10.6926	-0.52429	0.002524
NM_031185	Akap12	A-kinase anchor protein 12	chr10:4266328-4359471	44.7137	13.3177	-0.52601	0.002524

NM_001166065	Gcnt4	beta-1,3-galactosyl-O-glycosyl-glycoprotein beta-1,6-N-acetylglucosaminyltransferase 4	chr13:96924688-96950914	18.2365	5.41546	-0.52731	0.002524
NM_176933	Dusp4	dual specificity protein phosphatase 4	chr8:34807609-34819894	19.2549	5.67441	-0.53062	0.024582
NM_010637	Klf4	Krueppel-like factor 4	chr4:55527136-55532475	19.842	5.81933	-0.53271	0.007984
NM_178184	Hist1h2an	histone H2A type 1	chr13:21786771-21787218	188.747	55.3136	-0.53305	0.0266
NM_026411	1700021F05F	uncharacterized protein C6orf203 homolog	chr10:43525120-43540994	37.4616	10.8408	-0.53853	0.017468
NM_001136062	Eno3	beta-enolase	chr11:70657175-70662513	52.6291	15.1839	-0.53984	0.004567
NM_009450	Tubb2a	tubulin beta-2A chain	chr13:34074279-34078008	103.454	29.7065	-0.5419	0.002524
NM_007792	Csrp2	cysteine and glycine-rich protein 2	chr10:110920175-110939514	155.944	44.6608	-0.54304	0.002524
NM_175751	Zfp608	zinc finger protein 608	chr18:54888044-54990180	7.54033	2.14509	-0.54594	0.002524
NM_015747	Slc20a1	sodium-dependent phosphate transporter 1 isoform 1	chr2:129198772-129211612	98.1434	27.9014	-0.54623	0.002524
NM_173876	Clcn3	H(+)/Cl(-) exchange transporter 3 isoform c	chr8:60910388-60983311	19.6107	5.5717	-0.5465	0.033482
NM_028341	Ttc39c	tetratricopeptide repeat protein 39C	chr18:12643532-12737052	19.3157	5.43467	-0.55074	0.007984
NM_177628	Fam167a	protein FAM167A	chr14:63436393-63465502	10.1243	2.83387	-0.55299	0.012409
NM_146093	Ubxn1	UBX domain-containing protein 1	chr19:8871558-8875656	99.8456	27.9467	-0.553	0.002524
NM_001287800	Fhl1	four and a half LIM domains protein 1 isoform 3	chrX:56731760-56793346	55.4267	15.4192	-0.55566	0.006356
NM_008241	Foxg1	forkhead box protein G1	chr12:49382882-49386867	27.2724	7.49099	-0.56118	0.002524

NM_010329	Pdpn	podoplanin isoform 1 precursor	chr4:143267408-143299564	124.116	33.3687	-0.57049	0.002524
NM_199449	Zhx2	zinc fingers and homeoboxes protein 2	chr15:57694666-57839832	8.15335	2.16948	-0.57498	0.013662
NM_028778	Nuak2	NUAK family SNF1-like kinase 2 isoform B	chr1:132316124-132333488	10.0679	2.67823	-0.57509	0.034399
NM_011607	Tnc	tenascin precursor	chr4:63959784-64047015	255.879	67.762	-0.57705	0.002524
NM_001033270	Slc4a7	sodium bicarbonate cotransporter 3	chr14:14703024-14799943	47.8608	12.6581	-0.57761	0.002524
NM_139300	Mylk	myosin light chain kinase, smooth muscle	chr16:34784949-35002434	7.52944	1.98064	-0.57996	0.002524
NM_009263	Spp1	osteopontin isoform 4 precursor	chr5:104435110-104441053	2303.76	602.463	-0.58251	0.002524
NM_026268	Dusp6	dual specificity protein phosphatase 6	chr10:99263230-99267489	16.0448	4.11421	-0.59105	0.004567
NM_001190374	Adamtsl3	ADAMTS-like protein 3	chr7:82335693-82614448	32.3455	8.29101	-0.59121	0.002524
NM_009931	Col4a1	collagen alpha-1(IV) chain precursor	chr8:11198422-11312826	82.0151	20.9952	-0.59177	0.002524
NM_011055	Pde3b	cGMP-inhibited 3',5'-cyclic phosphodiesterase B	chr7:114415253-114537937	35.4053	9.04253	-0.59278	0.002524
NM_020332	Ank	progressive ankylosis protein	chr15:27466676-27594907	84.5168	21.5592	-0.59331	0.002524
NM_028013	Endod1	endonuclease domain-containing 1 protein precursor	chr9:14353989-14381242	6.87068	1.75198	-0.59347	0.041104
NM_178407	Arap2	arf-GAP with Rho-GAP domain, ANK repeat and PH domain-containing protein 2	chr5:62602445-62766177	7.43582	1.88258	-0.59658	0.004567

NM_008536	Tm4sf1	transmembrane 4 L6 family member 1	chr3:57287063-57301919	89.2286	22.513	-0.59807	0.002524
NM_172867	Zfp462	zinc finger protein 462	chr4:54947944-55083563	5.48957	1.38399	-0.5984	0.002524
NM_009373	Tgm2	protein-glutamine gamma-glutamyltransferase 2	chr2:158116404-158146392	23.2933	5.83337	-0.60131	0.002524
NM_011448	Sox9	transcription factor SOX-9	chr11:112782209-112787757	4.94263	1.22983	-0.60412	0.041104
NM_023794	Etv5	ETS translocation variant 5	chr16:22381312-22439570	20.6034	5.09593	-0.60672	0.002524
NM_032398	Plvap	plasmalemma vesicle-associated protein	chr8:71497752-71511769	13.831	3.30377	-0.62184	0.029348
NR_002865	Rnu11	N/A	chr4:132270078-132270186	22005.1	5210.25	-0.62566	0.006356
NM_172963	Mtcl1	microtubule cross-linking factor 1 isoform 2	chr17:66336981-66449750	4.67606	1.09942	-0.62872	0.007984
NM_029999	Lbh	protein LBH	chr17:72918304-72941946	23.3984	5.43764	-0.63378	0.002524
NM_013743	Pdk4	[Pyruvate dehydrogenase (acetyl-transferring)] kinase isozyme 4, mitochondrial	chr6:5483350-5496278	20.1692	4.57551	-0.64425	0.002524
NM_178695	Prrg4	transmembrane gamma-carboxyglutamic acid protein 4 precursor	chr2:104830740-104849850	11.247	2.55139	-0.64426	0.022426
NM_028448	Cenpv	centromere protein V	chr11:62524943-62539261	51.4654	11.4549	-0.65252	0.002524
NM_007729	Col11a1	collagen alpha-1(XI) chain precursor	chr3:114030539-114220326	10.7126	2.37312	-0.65458	0.002524

NM_001142916	Plod2	procollagen-lysine,2-oxoglutarate 5-dioxygenase 2 isoform 1 precursor	chr9:92538800-92608427	59.7613	13.0971	-0.65924	0.002524
NM_010151	Nr2f1	COUP transcription factor 1	chr13:78188972-78236564	19.4791	4.2548	-0.66069	0.002524
NM_030179	Clip4	CAP-Gly domain-containing linker protein 4 isoform 1	chr17:71769690-71864210	21.1517	4.54679	-0.66764	0.007984
NM_029797	Mnd1	meiotic nuclear division protein 1 homolog	chr3:84087933-84155786	19.1303	4.09874	-0.66907	0.035191
NM_008744	Ntn1	netrin-1 precursor	chr11:68209363-68386826	64.9911	13.8645	-0.67095	0.002524
NM_023716	Tubb2b	tubulin beta-2B chain	chr13:34127007-34130354	94.1351	19.7194	-0.67886	0.002524
NM_017464	Nedd9	enhancer of filamentation 1 isoform 2	chr13:41309915-41487360	43.1316	8.88605	-0.68609	0.002524
NM_009189	Six1	homeobox protein SIX1	chr12:73041826-73046712	11.8206	2.41788	-0.6892	0.021334
NM_001081445	Ncam1	neural cell adhesion molecule 1 isoform 1 precursor	chr9:49502145-49799069	39.8739	8.14195	-0.68996	0.002524
NM_145356	Zbtb7c	zinc finger and BTB domain-containing protein 7C	chr18:75820177-76148564	5.1673	1.0462	-0.69365	0.034399
NM_172118	Myl9	myosin regulatory light polypeptide 9	chr2:156775463-156781657	25.8819	5.2032	-0.69672	0.002524
NM_026481	Tppp3	tubulin polymerization-promoting protein family member 3	chr8:105467491-105471422	16.8427	3.38581	-0.69675	0.023435
NM_021451	Pmaip1	phorbol-12-myristate-13-acetate-induced protein 1	chr18:66458603-66465558	65.7818	13.2018	-0.69747	0.002524

NM_001033606	Acsl3	long-chain-fatty-acid--CoA ligase 3 isoform a	chr1:78657824-78707743	77.9089	15.4892	-0.70156	0.002524
NM_197996	Tspan15	tetraspanin-15	chr10:62185395-62231218	14.5916	2.89039	-0.70315	0.002524
NM_178200	Hist1h2bm	histone H2B type 1-M	chr13:21722043-21722526	386.078	75.3649	-0.70951	0.002524
NM_013737	Pla2g7	platelet-activating factor acetylhydrolase precursor	chr17:43568450-43612201	12.3743	2.39943	-0.71241	0.03705
NM_011348	Sema3e	semaphorin-3E precursor	chr5:14025275-14256689	9.58284	1.83498	-0.71786	0.002524
NM_144551	Trib2	tribbles homolog 2	chr12:15791726-15816785	23.6596	4.49496	-0.72128	0.002524
NM_010553	Il18rap	interleukin-18 receptor accessory protein precursor	chr1:40515361-40551705	22.82	4.31846	-0.72299	0.002524
NM_008630	Mt2	metallothionein-2	chr8:94172617-94173567	877.123	165.701	-0.72373	0.002524
NM_027268	Scrn1	secernin-1	chr6:54508815-54566382	40.3114	7.45912	-0.73274	0.002524
NR_045332	E330023G01	N/A	chr9:98748598-98820087	18.4009	3.36574	-0.73776	0.047481
NM_026931	1810011O10	uncharacterized protein C8orf4 homolog	chr8:24437615-24438946	26.6128	4.85164	-0.7392	0.004567
NM_026163	Pkp2	plakophilin-2	chr16:16213344-16272712	16.9726	3.08608	-0.74034	0.002524
NM_027756	Mfap3l	microfibrillar-associated protein 3-like isoform a precursor	chr8:60632824-60676731	8.11761	1.4635	-0.74403	0.002524
NM_009142	Cx3cl1	fractalkine precursor	chr8:94772179-94782426	56.1417	10.0659	-0.74643	0.002524
NM_008449	Kif5c	kinesin heavy chain isoform 5C	chr2:49619313-49774778	5.94996	1.03022	-0.76158	0.002524
NM_024223	Crip2	cysteine-rich protein 2	chr12:113140235-113145506	81.8977	14.1662	-0.76202	0.002524
NM_010357	Gsta4	glutathione S-transferase A4	chr9:78191965-78209349	81.8286	13.672	-0.77707	0.002524
NM_027924	Pdgfd	platelet-derived growth factor D precursor	chr9:6168611-6377519	12.7821	2.08495	-0.78751	0.032599

NM_178754	Arhgap6	rho GTPase-activating protein 6 isoform b	chrX:168795098-169304440	6.625	1.01689	-0.81391	0.030274
NM_183171	Fez1	fasciculation and elongation protein zeta-1	chr9:36843658-36878640	19.3859	2.91598	-0.8227	0.002524
NM_153122	Oplah	5-oxoprolinase	chr15:76294433-76307245	16.4528	2.43845	-0.82912	0.021334
NM_008344	Igfbp6	insulin-like growth factor-binding protein 6 precursor	chr15:102144185-102149512	36.3402	5.34524	-0.83242	0.007984
NM_011858	Tenm4	teneurin-4	chr7:96210636-96908554	5.3184	0.77212	-0.8381	0.002524
NM_175256	Heg1	protein HEG homolog 1 precursor	chr16:33684465-33768195	12.4091	1.74395	-0.85221	0.002524
NM_010158	Khdrbs3	KH domain-containing, RNA-binding, signal transduction-associated protein 3	chr15:68928419-69093518	17.3469	2.3219	-0.87338	0.012409
NM_001033167	Slc22a23	solute carrier family 22 member 23	chr13:34179157-34345182	8.17768	1.05146	-0.89084	0.004567
NM_172775	Plxnb1	plexin-B1 precursor	chr9:109095435-109119915	6.86198	0.881523	-0.89121	0.002524
NM_025446	Aig1	androgen-induced gene 1 protein	chr10:13652708-13868830	41.9277	5.36029	-0.89331	0.014951
NM_030889	Sorcs2	VPS10 domain-containing receptor SorCS2 precursor	chr5:36017180-36398139	11.7245	1.49756	-0.89371	0.002524
NM_029525	Prex2	phosphatidylinositol 3,4,5-trisphosphate-dependent Rac exchanger 2 protein isoform 1	chr1:10993464-11303682	6.50902	0.790213	-0.91577	0.002524
NM_008242	Foxd1	forkhead box protein D1	chr13:98354244-98356705	19.6114	2.29694	-0.93136	0.018718

NM_001111096	Lyn	tyrosine-protein kinase Lyn isoform A	chr4:3678120-3791612	20.8765	2.42563	-0.93483	0.002524
NM_009701	Aqp5	aquaporin-5	chr15:99591027-99594829	284.737	32.5648	-0.94169	0.002524
NM_008342	Igfbp2	insulin-like growth factor-binding protein 2 precursor	chr1:72824479-72852471	1303.34	134.567	-0.98612	0.002524
NM_028623	Cst6	cystatin-M precursor	chr19:5344704-5349574	3.9323	0.401577	-0.99088	0.046989
NM_011267	Rgs16	regulator of G-protein signaling 16	chr1:153740352-153745468	9.77833	0.994772	-0.99254	0.030274
NM_008597	Mgp	matrix Gla protein precursor	chr6:136872434-136875805	327.548	32.4363	-1.00425	0.002524
NM_010050	Dio2	type II iodothyronine deiodinase	chr12:90724551-90738438	9.45887	0.899066	-1.02205	0.004567
NM_172285	Plcg2	1-phosphatidylinositol 4,5-bisphosphate phosphodiesterase gamma-2	chr8:117498290-117635142	8.24819	0.772054	-1.02871	0.014951
NM_011057	Pdgfb	platelet-derived growth factor subunit B precursor	chr15:79995875-80014808	14.6014	1.36108	-1.03051	0.012409
NM_028474	Ptchd4	patched domain-containing protein 4	chr17:42315946-42507741	3.83291	0.34848	-1.04135	0.023435
NM_010228	Flt1	vascular endothelial growth factor receptor 1 precursor	chr5:147562195-147725988	11.1333	0.980385	-1.05523	0.002524
NM_172604	Scara3	scavenger receptor class A member 3	chr14:65919394-65953744	6.53407	0.568386	-1.06054	0.048088
NM_009504	Vdr	vitamin D3 receptor	chr15:97854426-97908296	15.1528	1.31599	-1.06124	0.002524
NM_001162926	Fam84b	protein FAM84B	chr15:60818995-60831400	14.5297	1.22884	-1.07276	0.009547
NM_008216	Has2	hyaluronan synthase 2	chr15:56665626-56694546	33.7748	2.64613	-1.10598	0.002524

NM_010736	Ltbr	tumor necrosis factor receptor superfamily member 3 precursor	chr6:125306570-125313870	41.6472	3.17411	-1.11797	0.006356
NM_018884	Pdzrn3	E3 ubiquitin-protein ligase PDZRN3	chr6:101149606-101377897	32.4978	2.43304	-1.1257	0.002524
NM_001001883	Hecw2	E3 ubiquitin-protein ligase HECW2 isoform 1	chr1:53806873-54195034	2.36392	0.173053	-1.13546	0.028592
NM_001303423	Aldoc	fructose-bisphosphate aldolase C	chr11:78323072-78327778	9.40185	0.675197	-1.14378	0.04259
NM_029620	Pcolce2	procollagen C-endopeptidase enhancer 2 precursor	chr9:95637627-95695551	16.6642	1.15055	-1.16088	0.030274
NM_031257	Plekha2	pleckstrin homology domain-containing family A member 2	chr8:25039143-25101811	18.1943	1.14061	-1.2028	0.002524
NM_021273	Ckb	creatine kinase B-type	chr12:111669354-111672338	293.966	18.2988	-1.20587	0.002524
NM_019645	Pkp1	plakophilin-1	chr1:135871394-135919024	20.5342	1.2765	-1.20646	0.002524
NM_001145937	Tenm3	teneurin-3 isoform 2	chr8:48225664-48674690	21.2404	1.30195	-1.21257	0.002524
NM_011254	Rbp1	retinol-binding protein 1	chr9:98422960-98446550	11.9059	0.718059	-1.2196	0.024582
NM_001285817	Dtna	dystrobrevin alpha isoform d	chr18:23310019-23659719	14.6089	0.857015	-1.23163	0.006356
NM_001111059	Cd34	hematopoietic progenitor cell antigen CD34 isoform 1 precursor	chr1:194938820-194976959	72.2597	4.21553	-1.23405	0.002524
NM_152803	Hpse	heparanase precursor	chr5:100679485-100719683	19.9799	1.14107	-1.24328	0.004567
NM_020486	Bcam	basal cell adhesion molecule precursor	chr7:19756137-19770532	30.163	1.6062	-1.27368	0.006356
NM_198702	Adgrl3	latrophilin-3 precursor	chr5:81021592-81795730	11.8182	0.622373	-1.2785	0.009547

NM_011352	Sema7a	semaphorin-7A precursor	chr9:57940134-57962865	15.8266	0.74301	-1.32839	0.021334
NM_001177797	Afap1l2	actin filament-associated protein 1-like 2 isoform 2	chr19:56912353-57008575	12.8155	0.599985	-1.3296	0.011083
NM_144810	Klhdc8a	kelch domain-containing protein 8A	chr1:132298625-132307357	71.5102	3.13348	-1.35834	0.002524
NM_023844	Jam2	junctional adhesion molecule B precursor	chr16:84774122-84826375	9.42675	0.36322	-1.41419	0.004567
NM_007974	F2rl1	proteinase-activated receptor 2 precursor	chr13:95511729-95525240	50.1071	1.61729	-1.49111	0.004567
NM_172471	Itih5	inter-alpha-trypsin inhibitor heavy chain H5 precursor	chr2:10153542-10256529	7.63122	0.158981	-1.68125	0.030274
NM_009704	Areg	amphiregulin preproprotein	chr5:91139614-91148432	71.8481	1.08656	-1.82036	0.029348
NM_001305844	Apoe	apolipoprotein E precursor	chr7:19696243-19699188	139.152	1.96816	-1.84943	0.002524
NM_001001979	Megf10	multiple epidermal growth factor-like domains protein 10 precursor	chr18:57133089-57297467	76.4997	0.646193	-2.0733	0.002524
NM_001163027	Hcrtr1	orexin receptor type 1 isoform 1	chr4:130130216-130139162	1.33256	0	-5000	0.002524
NM_001163516	Pex5l	PEX5-related protein isoform 2	chr3:32949633-33143191	1.92951	0	-5000	0.002524
NM_001177652	Gm5886	uncharacterized protein LOC545886 precursor	chr6:133763997-133767408	3.34024	0	-5000	0.002524
NM_001272033	AA414768	ubiquitin-conjugating enzyme E2 Q2-like	chrX:12936872-12938541	8.45929	0	-5000	0.002524

NM_001291189	Cdh26	cadherin-like protein 26 isoform b precursor	chr2:178430514-178487366	1.19477	0	-5000	0.002524
NM_001291292	2210011C24R	uncharacterized protein LOC70134	chr8:84010227-84011720	8.82022	0	-5000	0.002524
NM_001302206	Lmo1	rhombotin-1 isoform 3	chr7:109138564-109175207	1.37577	0	-5000	0.002524
NM_007843	Defb1	beta-defensin 1 precursor	chr8:21776554-21795185	1.25272	0	-5000	0.002524
NM_009181	St8sia2	alpha-2,8-sialyltransferase 8B precursor	chr7:73939119-74013682	6.03387	0	-5000	0.002524
NM_010090	Dusp2	dual specificity protein phosphatase 2	chr2:127336158-127338377	1.36513	0	-5000	0.002524
NM_010259	Gbp2b	interferon-induced guanylate-binding protein 1	chr3:142594846-142619176	1.85559	0	-5000	0.002524
NM_013603	Mt3	metallothionein-3	chr8:94152606-94154148	78.8609	0	-5000	0.002524
NM_021316	Cend1	cell cycle exit and neuronal differentiation protein 1	chr7:141426450-141429420	1.30435	0	-5000	0.002524
NM_021456	Ces1g	liver carboxylesterase 1 precursor	chr8:93302368-93337209	1.17525	0	-5000	0.002524
NM_024198	Gpx7	glutathione peroxidase 7 precursor	chr4:108400216-108406713	2.98493	0	-5000	0.002524
NM_026058	Cers4	ceramide synthase 4	chr8:4493404-4526079	6.02229	0	-5000	0.002524
NM_027650	Speer3	spermatogenesis associated glutamate (E)-rich protein 3	chr5:13791618-13796819	3.37321	0	-5000	0.002524
NM_029555	Gstk1	glutathione S-transferase kappa 1	chr6:42245934-42250441	1.13892	0	-5000	0.002524
NM_054084	Calcb	calcitonin gene-related peptide 2 precursor	chr7:114718642-114723365	7.06467	0	-5000	0.002524

NM_145132	Mchr1	melanin-concentrating hormone receptor 1	chr15:81235498-81238964	1.46881	0	-5000	0.002524
NM_177346	Gpr149	probable G-protein coupled receptor 149	chr3:62529962-62605140	1.29816	0	-5000	0.002524
NM_177375	Rab26	ras-related protein Rab-26	chr17:24529053-24533747	1.26123	0	-5000	0.002524
NR_003492	2210409E12F	N/A	chr11:88972637-88973014	19.7578	0	-5000	0.002524
NR_027826	Pantr1	N/A	chr1:42648199-42694825	16.2988	0	-5000	0.002524
NR_028299	1700084E18F	N/A	chr2:30237197-30237631	1.86507	0	-5000	0.016073
NR_033602	Gm5779	N/A	chr10:75352054-75352961	19.4556	0	-5000	0.002524
NR_045162	A330048O09	N/A	chr13:48272417-48273884	1.99497	0	-5000	0.002524
NR_104339	Adamtsl5	N/A	chr10:79697304-80369637	1.60196	0	-5000	0.011083

Footnotes: [1]Values represent FKPM from RNA Seq of Total RNA from both Control and UBXN1 KO MEFs; [2]Ratio of FKPM for KO over control, sorted from highest to lowest; in cases where one value was 0 fold-change arbitrarily set at +/-5000; [3]Corrected for multiple comparisons. It is likely that UBXN1 itself is not at -5000 because both the 5' and 3' ends of the mRNA would still be synthesized.
