# Supplementary material for: Multiple UBXN family members inhibit retrovirus and lentivirus production and canonical NFκΒ signaling by stabilizing IκBα
Source: PLoS Pathog. 2017 Feb 2;13(2):e1006187. doi: 10.1371/journal.ppat.1006187 (PMC5308826; doi:10.1371/journal.ppat.1006187)
Supplement: S4 Table — (PDF) [file ppat.1006187.s013.pdf]

Supplementary Table 4: KEGG Pathway Analysis of RNA-Seq UBXN1 KO vs. MEF control[1]

Pathway Names	Reference Genes in Pathway	Expected Genes In Pathway	Observed Genes In Pathway	Ratio of Enrichment	Raw p-value	Adjusted p-value[2]	EntrezGene IDs
Focal adhesion	199	4.81	32	6.65	2.04E-17	3.88E-16	12832 16775 57342 14205 16404 18750 98932 12827 53867 12833 16421 71785 12826 12443 12389 70549 18591 12843 16402 12814 107589 170736 12834 12390 20750 14254 12825 216148 26417 19211 12842 21826
Chemokine signaling pathway	183	4.42	15	3.39	4.18E-05	2.18E-09	20304 14772 15945 20306 20848 17096 216148 20847 14825 20315 20312 20846 26417 14696 14701
ECM-receptor interaction	86	2.08	16	7.7	2.29E-10	2.83E-07	12832 12843 16402 12814 16775 12834 16404 20750 12825 53867 12827 12833 16421 12842 12826 21826
Protein digestion and absorption	78	1.88	13	6.9	4.47E-08	6.33E-07	12832 12843 12814 12834 72472 67760 72461 12825 53867 12827 12833 12842 12826
Axon guidance	130	3.14	15	4.77	6.24E-07	2.41E-06	223881 19876 20356 18208 20361 107449 20564 20349 20315 22253 268902 26417 108151 235611 17974

Cytokine-cytokine receptor interaction	245	5.92	21	5.92	6.33E-07	4.08E-06	18414 12156 16177 16324 20306 14205 14825 16164 20315 14102 20312 71785 14600 18591 20304 15945 17000 14254 16174 16880 17311
Pathways in cancer	322	7.78	24	3.08	1.29E-06	4.08E-06	22410 12156 15245 16775 20848 14205 16451 18750 12827 14102 20846 14013 17390 12826 12443 18591 22033 234779 14367 12159 26417 19211 14183 17311
Amoebiasis	116	2.8	13	4.64	4.08E-06	1.13E-05	12832 12843 12814 20708 16177 16775 12825 18750 53867 12827 20706 12842 12826
Metabolic pathways	1175	28.39	53	1.87E+00	1.13E-05	8.82E-05	319554 17158 108156 12709 14595 67680 11898 12091 14629 15452 23827 52538 20444 15931 66988 66491 140481 11676 230815 13808 110391 234779 100678 19224 16891 27226 15442 18704 17156 23972 68342 18263 20917 17995 269823 11881 22169 74241 11947 67273 218476 20020 110196 11669 66445 13595 15488 98711 241452 14718 18605 12857 74205
Lysosome	123	2.97	11	3.7	0.0002	0.0004	11883 14667 11881 16889 12091 229445 12751 74105 13030 67963 15931

Footnotes: [1]Anaylsis perfomed by submitting list of differentially expressed genes to WebGestalt server and selecting KEGG Analysis and entire mouse genome as reference set; [2]Multiple correction testing performed using Benjamini & Hochberg test
